# Conformational cycling of the Wntless transporter drives trafficking and secretion of Wnt morphogens

**DOI:** 10.1038/s41467-026-74521-y

**Published:** 2026-06-19

**Authors:** Yunhui Ge, Taciani de Almeida Magalhaes, Hongjiang Wu, Dick J. H. van den Boomen, Thu Uyen Nguyen, Tongyi Dou, Gaya P. Yadav, Sukyeong Lee, Zhao Wang, Andrew Lemoff, Xuemei Luo, Sumitha S. Menon, Min Zhang, Jin Wang, Zhicheng Jin, Jiansen Jiang, Adrian Salic, Pengxiang Huang

**Affiliations:** 1 Department of Molecular and Cellular Biology, Baylor College of Medicine, Houston, TX USA; 2 Department of Cell Biology, Blavatnik Institute, Harvard Medical School, Boston, MA USA; 3 Verna and Marrs McLean Department of Biochemistry and Molecular Pharmacology, Baylor College of Medicine, Houston, TX USA; 4 Laboratory of Membrane Proteins and Structural Biology, Biochemistry and Biophysics Center, National Heart, Lung, and Blood Institute, National Institutes of Health, Bethesda, MD USA; 5 Laboratory for Biomolecular Structure and Dynamics (LBSD), Department of Biochemistry and Biophysics, Texas A&M University, College Station, TX USA; 6 Advanced Technology Core for Macromolecular X-ray Crystallography, Baylor College of Medicine, Houston, TX USA; 7 Department of Biochemistry, University of Texas Southwestern Medical Center, Dallas, TX USA; 8 Massachusetts College of Pharmacy and Health Sciences, Boston, MA USA; 9 University of Wisconsin Hospital and Clinics, Madison, WI USA; 10 Department of Pathology and Laboratory Medicine, University of Wisconsin-Madison, Madison, WI USA

**Keywords:** Cryoelectron microscopy, Morphogen signalling, Membrane proteins, Molecular conformation

## Abstract

Wnt proteins are lipid-modified morphogens fundamental in development and disease. During Wnt biogenesis, the G-protein-coupled receptor (GPCR)-like transporter Wntless (WLS) escorts lipidated Wnts from the endoplasmic reticulum to the plasma membrane, then transfers them to extracellular carriers, forming active and soluble morphogen-carrier complexes. To dissect the mechanisms involved, we solve cryo-EM structures of Wnt-bound WLS and unliganded WLS, and perform structure-guided functional experiments. Wnts engage WLS via three conserved hairpins, which are all required for Wnt trafficking to the cell surface and carrier-mediated secretion. Wnt release from cells is driven by dramatic conformational changes in the WLS transmembrane domain, reminiscent of GPCR activation, together with WLS extracellular rearrangements. Unexpectedly, we find that Wnt5a bound to WLS forms dimers, with implications for Wnt signaling. These findings define the mechanism of WLS conformational cycling that governs the intracellular transport and extracellular release of Wnt morphogens, essential steps in the Wnt pathway.

## Abstract (web summary)

Wnt morphogens are released from signaling cells in complex with carrier proteins, to offset insolubility due to lipidation. Here, the authors dissect the mechanism of Wnt release and show it is governed by conformational cycling of the G-protein-coupled receptor-like transporter Wntless.

## Introduction

Wnt morphogens are a family of secreted signaling molecules, comprising 19 members in humans and mice, that play fundamental roles in embryogenesis, tissue regeneration and cancer^1–4^. Wnts activate signaling by simultaneously binding on the surface of target cells to a GPCR-related Frizzled (FZD) receptor and to a co-receptor^2^. Based on the co-receptor they employ, which dictates the downstream signaling events that are triggered, Wnts are classified into canonical and non-canonical. Canonical Wnts, such as Wnt3a, recruit low-density lipoprotein receptor-related proteins LRP5 or LRP6 as co-receptors and activate signaling by stabilizing the transcriptional coactivator β-catenin, thereby driving target gene expression^2,3,5^. In contrast, non-canonical Wnts, such as Wnt5a, employ tyrosine kinase-like orphan receptors ROR1 or ROR2 as co-receptors, and lead to the activation of β-catenin-independent, non-transcriptional downstream events^2,6–10^. Non-canonical Wnt signaling governs critical aspects of tissue morphogenesis, and its dysregulation causes birth defects and is linked to cancer metastasis^7,11–13^; however, it remains poorly understood mechanistically compared to canonical Wnt signaling.

All Wnts undergo covalent modification with palmitoleic acid (PAM), which forms an ester linkage with the side chain of a conserved serine residue, a reaction that occurs in the endoplasmic reticulum (ER)^14^ and is catalyzed by Porcupine (PORCN)^15–17^, a member of the membrane-bound O-acyltransferase (MBOAT) family. PAM-modified Wnts bind the GPCR-like membrane protein Wntless (WLS, also known as GPR177)^18,19^, which then transports them from the ER to the plasma membrane, for secretion. Mutating the PAM acceptor serine in Wnt, pharmacological inhibition of PORCN, or WLS knockout causes Wnt retention in the ER^20–22^ and thus abolishes secretion, indicating that both Wnt PAM modification and WLS are absolutely required for Wnts to reach the plasma membrane of producing cells.

PAM modification is critical for the signaling activity of Wnts, being essential for their interaction with FZD receptors, which includes a key contact between the PAM moiety and the extracellular cysteine-rich domain (CRD) of FZDs^23–25^. At the same time, PAM modification makes Wnts highly hydrophobic and thus strongly membrane-tethered, posing a challenge to their secretion and spreading to distant target cells. The serum albumin family protein, afamin (AFM), was identified as an extracellular carrier that can solubilize lipidated Wnts, leading to their release from cells^26,27^. In recent work, we demonstrated that two additional classes of extracellular proteins, secreted FZD-related proteins (SFRPs)^28,29^ and Wnt inhibitory factor 1 (WIF1)^30^, are Wnt-specific carriers, accepting Wnts from WLS through a direct handoff mechanism^22^. All these different carriers form active and soluble complexes by shielding the Wnt PAM moiety from the aqueous environment, thus enabling Wnt morphogen diffusion through extracellular space^22,31,32^. On the surface of target cells, glypicans (GPCs) catalyze the lipid-dependent Wnt transfer from carriers to FZDs^22,33^, leading to Wnt pathway activation. Thus, PAM modification is pivotal to every aspect of Wnt biology, beginning with biogenesis and intracellular trafficking in producing cells, secretion and extracellular distribution, and ultimately reception and Wnt pathway activation in target cells. These processes rely on a dedicated network of lipid-dependent Wnt interactors, among which Wnt ligands are exchanged via rapid handoff.

Recent structural studies of canonical Wnts in complex with WLS have provided valuable insight into how Wnts engage WLS through three conserved hairpins (“fingers”), one of which includes the PAM-modified serine residue^34–36^. However, it is unclear if and how the hairpins and their interacting sites on WLS are required for Wnt transport to the cell surface. Furthermore, the role of these interactions in the critical transfer of Wnts from WLS to extracellular carriers is unknown, given that previous studies of WLS were performed in the absence of Wnt-specific carriers^34–36^. Additionally, the modest resolution of the structure of WLS in apo form precludes a mechanistic understanding of how Wnts are released from WLS during secretion^36^. Finally, non-canonical Wnts have not yet been investigated structurally, leaving open the question of what confers their specific activity.

In this work, to dissect the mechanisms of Wnt trafficking and secretion, we determine the cryo-EM structure of WLS in complex with the prototypical non-canonical Wnt, Wnt5a, at 2.64 Å resolution. Structure-guided functional assays reveal that all three Wnt hairpins are essential for Wnt surface localization and carrier-mediated secretion. We also determined the 2.63 Å cryo-EM structure of WLS by itself, reconstituted in native membrane nanodiscs, thus showing the conformation that WLS adopts after Wnt release. Comparison of these high-resolution structures reveals dramatic structural rearrangements within the WLS transmembrane domain, topologically similar to those that occur in GPCRs upon activation, along with previously unrecognized conformational changes in WLS extracellular regions, all of which we show to be critical for Wnt release from cells by extracellular carriers. Notably, our work provides the structure of a non-canonical Wnt and suggests explanations for why Wnt5a does not interact with LRP5/6 or the Wnt7-specific coreceptor, RECK. Unexpectedly, we also discover and solve the structure of a 2:2 Wnt5a-WLS complex, suggesting that Wnt5a can form an asymmetric homodimer upon secretion, with important implications for its downstream signaling activity.

## RESULTS

### Structure of human WLS in complex with non-canonical Wnt5a

**Fig. 1:**
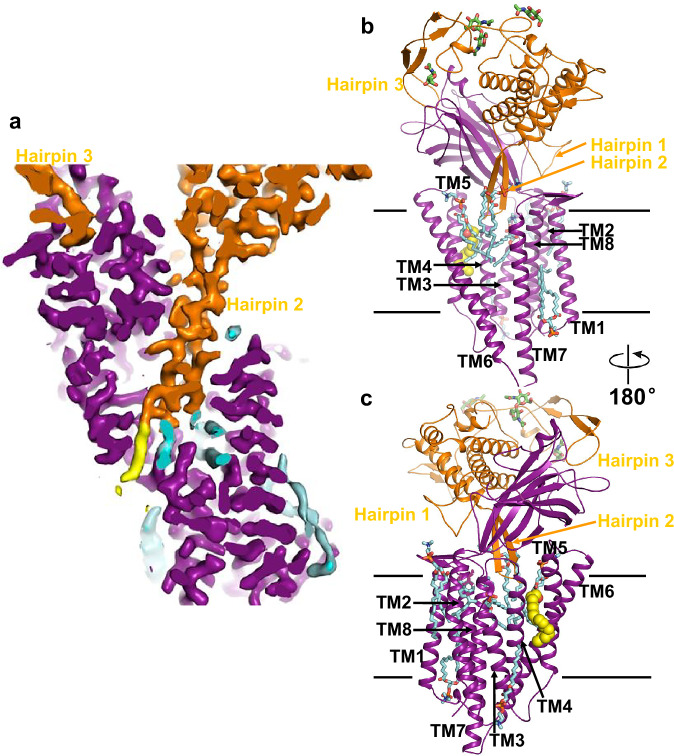
Structure of Wnt5a bound to WLS. **a** Cryo-EM density map of the Wnt5a-WLS complex at 2.64 Å resolution. Densities for Wnt5a, WLS and the PAM moiety are colored in orange, deep purple and yellow, respectively. Membrane lipid densities are shown in cyan. The same color scheme is used in (**b–c**). **b** Overall structure of the Wnt5a-WLS complex. The PAM moiety is shown as space-filling spheres. The glycans (green) and membrane lipids (cyan) are displayed as sticks. Transmembrane (TM) helices are numbered (TM1-TM8). The membrane bilayer is indicated by solid lines. Wnt5a interacts with WLS through three conserved hairpin fingers (hairpin 1-3). **c** As in (**b**), but rotated by 180°.

Structural characterization of canonical Wnts has so far been achieved only as part of complexes with binding partners, such as the FZD CRD or WLS, which shield the hydrophobic PAM modification^23–25,34–36^. To gain mechanistic insight into non-canonical Wnt trafficking and secretion, we co-expressed and purified human Wnt5a bound to human WLS (Supplementary Fig. 1). Using cryo-EM, we solved the structure of the 1:1 Wnt5a-WLS complex at 2.64 Å resolution, in the detergent glyco-diosgenin (GDN) (Fig. 1; see also Supplementary Figs. 2–7 for the cryo-EM workflows of all reported structures). The resulting model accounts for nearly the entire WLS and Wnt5a sequences, with the exception of the disordered C-terminal tail of WLS (residues 497–541), an N-terminal segment of Wnt5a immediately following the signal peptide (residues 36–43), and a loop in Wnt5a (residues 52–67). Additionally, the density map (Supplementary Fig. 8) reveals several lipid molecules associated with the WLS transmembrane domain (TMD) as well as three of the four predicted N-linked glycans on Wnt5a (on residues N114, N312, and N326, but not N120).

The overall architecture of the Wnt5a-WLS complex is nearly identical to the structures of WLS bound to canonical Wnts, including Wnt8a, Wnt3a, and Wnt7a^34–36^ (Supplementary Fig. 9a, b). WLS consists of a TMD with eight transmembrane helices (TMs), with TM2-TM8 in the Wnt-bound conformation resembling the seven-transmembrane (7TM) bundle of the class B GPCR glucagon receptor^37,38^, as identified by a structural similarity search using the DALI server^34,35,39^ (Supplementary Fig. 9e–g). Additionally, a luminal WLS domain inserts between TM1 and TM2, containing eight antiparallel β-sheets and adopting the globular fold of seipin, an ER protein involved in lipid droplet formation^40,41^ (Fig. 1b, c, Supplementary Fig. 10a). Interestingly, this domain arrangement of WLS is similar to that of members of the recently identified Golgi-dynamics (GOLD) domain 7TM helix (GOST) protein family, exemplified by TMEM87A^42,43^ (Supplementary Fig. 9c). The luminal domain of WLS shares some similarity with the GOLD domain, and WLS TM2-TM8 are homologous to the 7TM bundle of GOST proteins (Supplementary Fig. 9c, d). The cellular functions of GOST proteins, however, remain unknown.

Structurally, all Wnts consist of two distinct regions: an N-terminal domain (NTD), which features a saposin-like fold, a conserved structural module present in many lipid-interacting proteins, with two hairpin loops stemming from the core helical bundle (hairpin 1 and 2); and a C-terminal domain (CTD) characterized by a cystine-knot cytokine-like fold that forms the third hairpin loop (hairpin 3)^44^ (Supplementary Fig. 11). These two domains are connected by a linker that varies in length and sequence, which harbors the LRP5/6 co-receptor-binding site in canonical Wnts^25^. Thus, both WLS and Wnts contain domains structurally analogous to those involved in lipid interactions (the WLS luminal domain and the Wnt NTD), as well as those linked to signaling functions (the WLS transmembrane domain and the Wnt CTD).

**Fig. 2:**
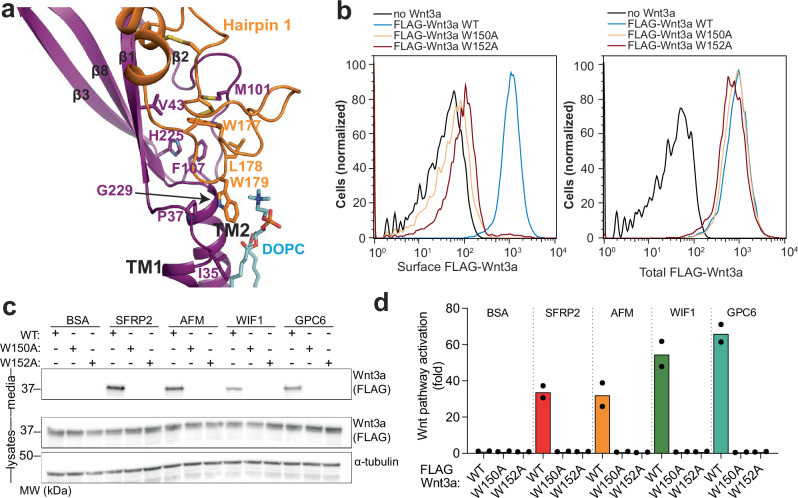
Interface between Wnt5a hairpin 1 and WLS. **a** Close-up view of the interaction between Wnt5a (orange) hairpin 1 and WLS (deep purple). The DOPC molecule is in cyan. **b** Surface (left, without membrane permeabilization) and total (right, with membrane permeabilization) levels of FLAG-mWnt3a (WT and indicated mutants) were measured by staining cells with anti-FLAG-M1-AlexaFluor647 and FACS analysis. Cells not expressing Wnt3a served as a negative control. **c** Purified SFRP2, AFM, WIF1, or GPC6 ectodomain (1 μM) were added to HEK293 cells expressing FLAG-mWnt3a constructs, and mWnt3a released into serum-free medium was analyzed by immunoblotting. Incubation with bovine serum albumin (BSA, 1 μM) served as a negative control. **d** As in (**c**), but the conditioned media were applied to Wnt reporter cells, and Wnt pathway activity was measured using the Dual-Glo luciferase assay. Data represent the average activation from two technical replicates (*n* = 2), normalized to the negative control, for a representative experiment. Each experiment has been performed at least twice using distinct biological samples.

Wnt5a grasps WLS from the luminal side, extensively engaging through its hairpins, to form three interfaces conserved for all Wnts (Fig. 1b, c). Hairpin 1, though less well-structured and more divergent among Wnt sequences, contains two tryptophan residues, W177 and W179, within a conserved WXWGG motif (Supplementary Fig. 11). In our structure, W177 inserts into a hydrophobic cavity at the base of WLS luminal domain, while W179 packs against the hydrophobic surface at the luminal end of TM2 (Fig. 2a). Strong density, likely corresponding to a phospholipid head group, is observed perpendicular to the indole ring of W179 (Supplementary Fig. 8a). Based on its chemical environment, we assign this lipid as phosphatidylcholine (DOPC), with its positively charged quaternary amine head group forming a π-cation interaction with W179 (Fig. 2a). Thus, the interaction between Wnt5a hairpin 1 and WLS is membrane-dependent, similar to peripheral membrane proteins^45^.

**Fig. 3:**
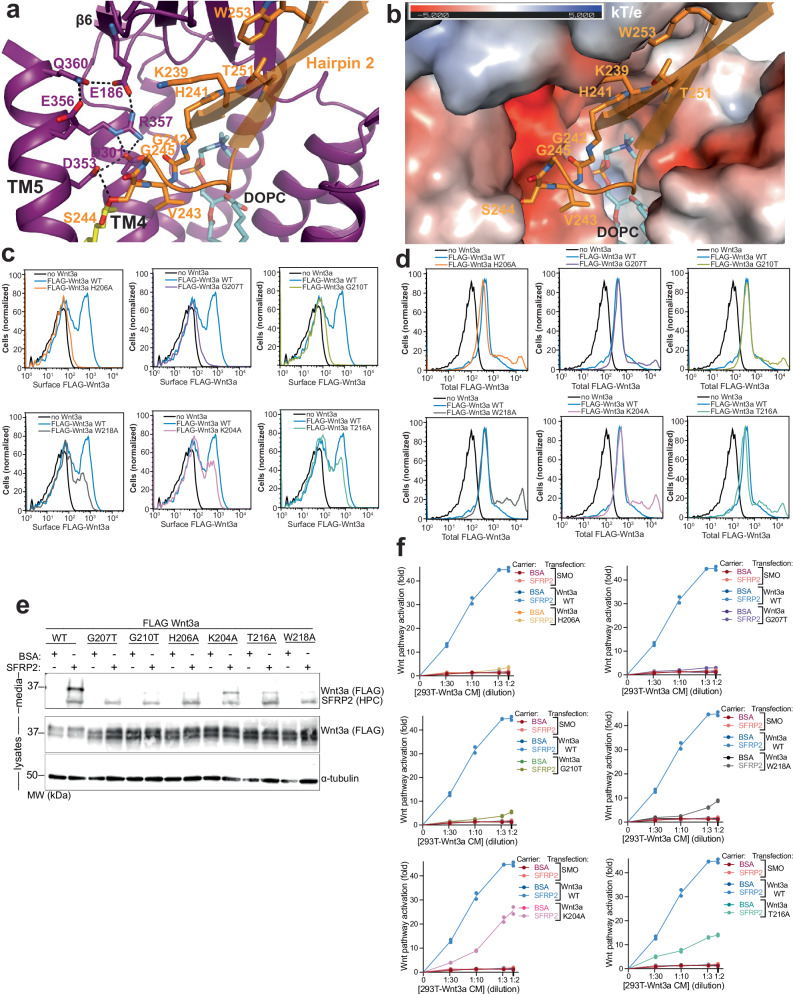
Interface between Wnt5a hairpin 2 and WLS. **a** Close-up view of the interaction between hairpin 2 of Wnt5a (orange) and WLS (deep purple), highlighting the hydrogen bond network (dashed lines) that coordinates the acylated S244 residue. The PAM moiety is shown in yellow and the DOPC molecule in cyan. **b** As in (**a**), but showing the electrostatic surface of the WLS cavity. **c** Cells stably expressing FLAG-mWnt3a constructs were stained with anti-FLAG-M1-AlexaFluor647 without membrane permeabilization and were analyzed by FACS to determine cell surface localization. **d** As in (**c**), but with membrane permeabilization, to determine total expression of FLAG-mWnt3a constructs. **e** Purified human protein C epitope (HPC)-tagged SFRP2 (1 μM) was added to HEK293 cells expressing FLAG-mWnt3a constructs, and release into serum-free medium was analyzed by anti-FLAG immunoblotting. Incubation with BSA (1 μM) served as a negative control. **f** As in (**e**), but serial dilutions of conditioned media were applied to Wnt reporter cells, and pathway activity was measured using Dual-Glo luciferase assay. Cells expressing mouse Smoothened (SMO) were used as a negative control. Data represent the average activation from two technical replicates (*n* = 2), normalized to the negative control, for a representative experiment. Each experiment has been performed at least twice using distinct biological samples.

In the Wnt-bound state, the WLS TMD features a large hydrophobic central cavity with a wide V-shaped opening facing the membrane between TM6 and TM7 (Fig. 1b). The extended β-strand hairpin 2 of Wnt5a, which bears the PAM-modified S244 at its tip, is embedded within this cavity, with the PAM moiety traversing between TM4 and TM5 (Figs. 1c, 3a). The esterified oxygen of the S244 side chain is coordinated by D353 from TM5 and stabilized through a network of hydrogen bonds and ionic interactions involving D301 from TM4, E356, R357, and Q360 from TM5, as well as E186 from the luminal domain. Together, these residues create an electronegative surface inside the hydrophobic cavity (Fig. 3a, b). In addition to several lipid molecules lining the cavity entrance, one resides within the cavity, with its head group buried deeply (Fig. 3a, b, Supplementary Fig. 8d), likely stabilizing the Wnt-bound open conformation of the WLS TMD; we assign it as DOPC, consistent with previous structures^35,36^.

**Fig. 4:**
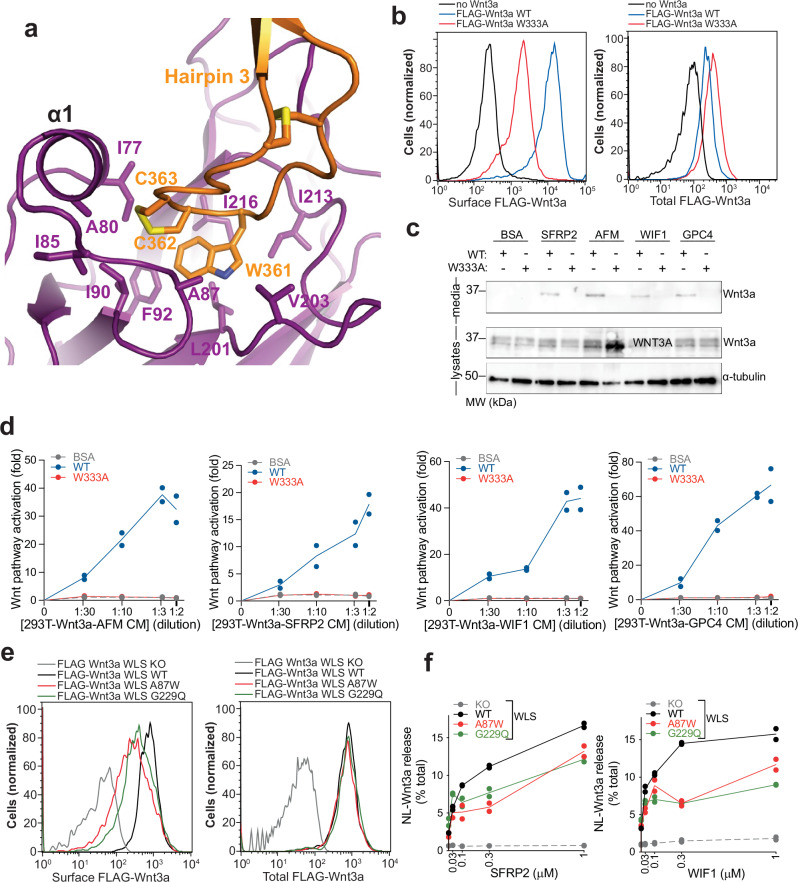
Interface between Wnt5a hairpin 3 and WLS. **a** Close-up view of the interaction between hairpin 3 of Wnt5a (orange) and WLS (deep purple). **b** Surface (left, without membrane permeabilization) and total (right, membrane permeabilization) levels of FLAG-mWnt3a (WT or W333A), stained with anti-FLAG-M1-AlexaFluor647 and analyzed by FACS. **c** Purified carriers (1 μM) were added to HEK293 cells stably expressing mWnt3a (WT or W333A), and release into serum-free medium was analyzed by anti-FLAG immunoblotting. Incubation with BSA (1 μM) served as a negative control. **d** As in (**c**), but serial dilutions of conditioned media were applied to MEF pBAR reporter cells, and Wnt pathway activity was measured by Dual-Glo luciferase assay. Data represent average activation from two technical replicates (*n* = 2), normalized to the negative control, for a representative experiment. Each experiment has been performed at least twice using distinct biological samples. **e** Surface and total FLAG-mWnt3a was measured as in (**b**) in WLS-KO cells stably expressing the indicated WLS constructs. **f** NanoLuc luciferase (NL)-mWnt3a release assay. WLS-KO cells stably expressing NL–mWnt3a together with the indicated WLS constructs were pre-incubated with cycloheximide (CHX, 50 μg/mL) to inhibit protein synthesis. NL-mWnt3a release was then initiated by adding varying concentrations of purified SFRP2 or WIF1 in serum-free medium, and was measured by NL luminescence. Data represent the average of two technical replicates (*n* = 2), normalized to total NL–mWnt3a in cell lysates, for a representative experiment. Each experiment has been performed at least twice using distinct biological samples.

The β-hairpin 3 of Wnt5a contains a conserved WCC motif at the distal tip, where C362 and C363 form an unusual tandem disulfide bond following W361 (Supplementary Fig. 11). These three residues bind to a hydrophobic cavity on the top of the WLS luminal domain, primarily formed by hydrophobic residues from connecting loops, including a short helix (denoted α1) located between the β1 and β2 strands (Fig. 4a). Notably, when binding to FZD CRD, Wnt hairpin 1 and hairpin 2 are packed together, while hairpin 2 and hairpin 3 engage in lipid-protein and protein-protein interactions, respectively, forming two distinct contact sites on FZD CRD^23–25^. In contrast, when binding to WLS, hairpin 1 is separated from hairpin 2, and all three fingers undergo conformational changes, as described^34,35^.

### Essential role of Wnt hairpins in Wnt surface localization and carrier-mediated secretion

Previous functional studies utilized co-immunoprecipitation (co-IP) to test Wnt-WLS binding^34–36^. However, co-IPs cannot reliably distinguish between specific and non-specific binding, given the highly hydrophobic nature of lipidated Wnts and of the WLS TMD. Furthermore, Wnt secretion assays were previously conducted without physiological Wnt carriers^34–36^. Finally, it is unclear whether the reported phenotypes of Wnt mutants are caused by impaired PAM modification rather than the intended structural alteration. We thus designed mutations targeting all three distinct Wnt hairpin-WLS contacts individually, and systematically assessed their functional impact. Given the high degree of conservation of these interfaces across all Wnts (Supplementary Fig. 11), we first focused on Wnt3a (Supplementary Fig. 10b), the best characterized Wnt, for a comprehensive functional analysis. We employed a series of quantitative assays for mutant analysis: 1) fluorescence-activated cell sorting (FACS), to assess cell surface and total levels of Wnt3a; 2) a robust and sensitive NanoLuc luciferase (NL)-based assay, to measure carrier-mediated Wnt3a secretion^22^; 3) measurement of Wnt pathway activation by conditioned media generated from Wnt3a-expressing cells in presence of purified carriers; and 4) mass spectrometry, to determine whether the introduced mutations affect Wnt3a PAM modification.

We first introduced point mutations into hairpin 1 of mouse Wnt3a (mWnt3a). The W150A and W152A mutations (corresponding to W177A and W179A in human Wnt5a, Fig. 2a), which disrupt the interaction of hairpin 1 with WLS, drastically reduced mWnt3a surface localization and abolished its release by the carriers SFRP2, AFM and WIF1, and by the ectodomain of the glypican GPC6 (Fig. 2b, c). Likewise, conditioned media from cells expressing mWnt3a W150A or W152A in the presence of carriers did not activate Wnt signaling in reporter cells (Fig. 2d). This contrasts with a previous report suggesting that W150A has minimal impact on Wnt secretion and autocrine signaling^35^. We speculate that a possible explanation for this discrepancy is that an unknown serum component might mediate Wnt secretion, or that autocrine signaling is the result of direct transfer of Wnt from WLS to Frizzled receptors.

We next examined multiple conserved residues within lipidated hairpin 2, which are positioned in the WLS central binding cavity (Fig. 3a, b). Mutations G207T, G210T and H206A completely abolished mWnt3a surface localization and secretion, while K204A, T216A and W218A markedly reduced but didn’t completely eliminate these readouts (Fig. 3c, e, f). Importantly, all mWnt3a mutants tested had similar total expression levels (Figs. 2b, 3d). Finally, LC-MS/MS analysis of immunopurified mWnt3a confirmed that PAM modification of S209 was present in all mutants except G207T (Supplementary Fig. 12), in which lipidation was completely abolished, likely due to interference with PORCN binding^17^.

The most striking discrepancy was observed with mutations affecting Wnt hairpin 3. Previous studies claimed that hairpin 3 is dispensable for WLS interaction and Wnt secretion, but is required for engaging FZD CRD^34,35^. In contrast, we find that the mutant mWnt3a W333A (corresponding to W361 in the WCC motif of human Wnt5a hairpin 3, Fig. 4a), showed drastically reduced cell surface localization, despite being expressed at a level comparable to that of the wild-type (WT) protein (Fig. 4b) and being properly PAM-modified (Supplementary Fig. 12). The W333A mutation also completely abolished mWnt3a secretion mediated by all tested carriers, including AFM, SFRP2, WIF1, and GPC4 (Fig. 4c, Supplementary Fig. 13a). Consistent with this result, conditioned media from cells expressing mWnt3a W333A in the presence of carriers, failed to activate Wnt/β-catenin signaling (Fig. 4d).

To further validate our findings, we designed WLS mutations that disrupt the observed interfaces with Wnt (Supplementary Fig. 10a) and tested them functionally by stable expression in WLS-knockout (KO) HEK293 cells that also express mWnt3a. Consistent with previous reports^34^, the mWLS G229Q mutation, which introduces a steric clash with W179 in hairpin 1 of human Wnt5a (Fig. 2a, corresponding to W152 in mWnt3a), impaired mWnt3a surface localization and carrier-mediated secretion (Fig. 4e, f). Next, we tested mWLS A87W, which adds a bulky side chain, to block the binding site for the Wnt hairpin 3 WCC-motif (Fig. 4a); this mWLS mutant significantly reduced surface localization and carrier-mediated secretion of Wnt3a, while mWnt3a total expression levels were unaffected (Fig. 4e, f). Importantly, the tested mWLS mutants exhibited expression levels comparable to those of WT mWLS (Supplementary Fig. 13b).

We performed similar assays to investigate the role of Wnt hairpins in trafficking and secretion of the non-canonical Wnt, Wnt5a. We focused on hairpins 1 and 3, which show the most pronounced discrepancies with previous reports^34,35^, whereas the lipidated hairpin 2 is known to be essential for all Wnts. Wnt5a W177A and W179A (hairpin 1), as well as Wnt5a W361A (hairpin 3), showed markedly reduced surface localization and carrier-mediated secretion (Supplementary Fig. 14). Accordingly, the mWLS G229Q and A87W mutations, which disrupt interactions with hairpins 1 and 3, had similar effects on Wnt5a as on Wnt3a (Supplementary Fig. 15, Fig. 4e, f). Together, these results demonstrate that interactions between all three Wnt hairpins and WLS are critical for transport to the cell surface and secretion of both canonical and non-canonical Wnts.

### Conformational WLS transitions driving Wnt release

**Fig. 5:**
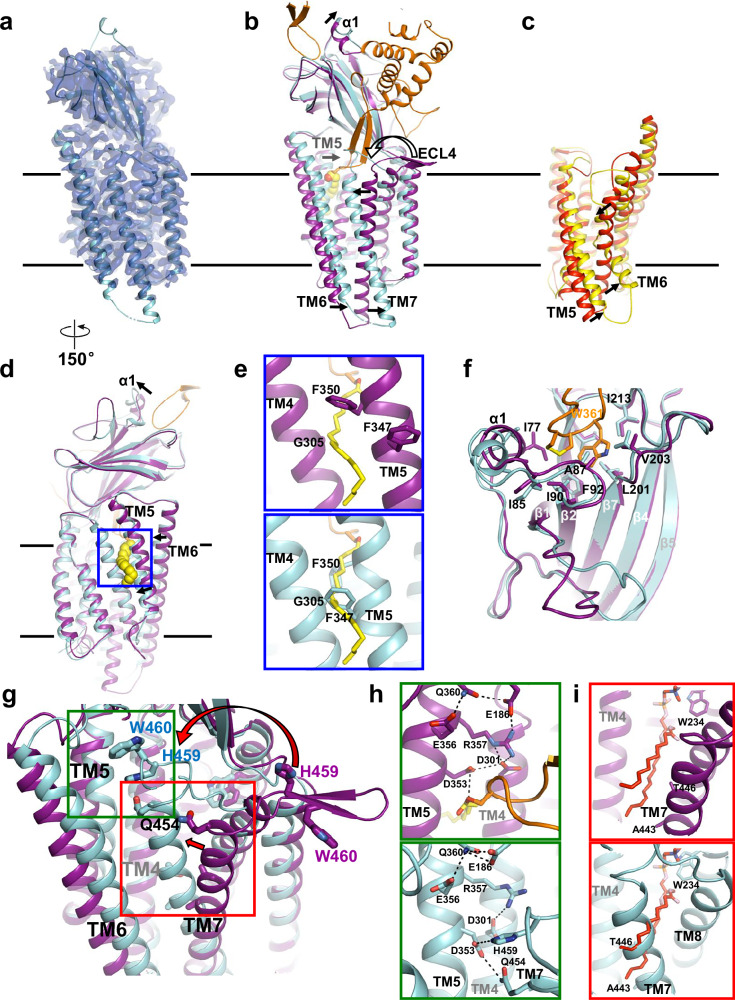
WLS conformational transitions upon Wnt5a release. **a** Fitting of the apo WLS structure into the 2.63 Å-resolution cryo-EM density map, contoured at 10σ. **b** Structural comparison of Wnt5a-bound WLS (deep purple) and apo WLS (cyan). Wnt5a is shown in orange, with the PAM moiety as yellow spheres. The same color scheme is used in panels (**d–i**). The membrane bilayer is indicated by solid lines, and arrows highlight the movement of indicated structural elements. **c** Same view as (**b**), but comparing the conformational changes of the glucagon receptor between G protein-free state (red, PDB ID: 5YQZ) and G protein-engaged fully activated state (yellow, PDB ID: 6WPW). **d** As in (**b**), but rotated by 150°, highlighting TM5 movement. **e** Close-up view of the blue square region in (**d**), comparing TM5 conformation between Wnt5a-bound state (top) and apo state (bottom). In apo WLS, F347 and F350 would sterically clash with the Wnt lipid moiety (yellow). **f** Comparison of the WLS luminal domain in Wnt5a-bound and apo states, highlighting the outward movement of the α1 helix, to facilitate release of Wnt hairpin 3. **g** Conformational changes in the TM region and ECL4 of WLS upon Wnt release. Key residues are shown as sticks. **h** Close-up view of the green square region in (**g**), comparing the hydrogen bond network (dashed lines) between Wnt5a-bound state (top) and apo state (bottom). **i** Close-up view of the red square region in **g**. In the Wnt5a-bound state (top), a DOPC molecule (red) is deeply buried within the WLS central cavity. In the apo state, the movement of TM7 closes the central cavity, causing a steric clash with DOPC.

Given that WLS adopts a GPCR-like fold, we hypothesized that it undergoes conformational cycling analogous to those of classical GPCRs, to facilitate Wnt release. To test this idea, we determined the cryo-EM structure of WLS in its apo state, embedded in lipid bilayer nanodiscs, at 2.63 Å resolution (Fig. 5a, Supplementary Figs. 4, 5, 16). The structure reveals that TM7 in WLS undergoes a dramatic counterclockwise rotation, accompanied by a subtler movement of TM6, leading to the closure of the large cavity that accommodates the PAM-modified Wnt hairpin 2 (Fig. 5b). These two helices are equivalent to TM6 and TM5 in GPCRs, with the hallmark movement of TM6 being a defining feature of GPCR activation^37,38,46^ (see Fig. 5c, Supplementary Fig. 9e–g and Supplementary Video 1, 2 for a comparison of WLS with the glucagon receptor).

Because of WLS TM7 rotation, the extracellular β-hairpin loop 4 (ECL4), which connects TM7 and TM8 in the Wnt-bound conformation, completely unfolds, undergoing a dramatic conformational change to fill the cavity vacated by Wnt (Fig. 5b, g); this structural transition was not resolved in the reported low-resolution apo WLS structure^36^. In the Wnt-bound state, the Wnt-binding cavity harbors a polar interaction network to coordinate the PAM-modified serine (Fig. 3a). Upon Wnt release, these residues reorganize to maintain interactions (Fig. 5h), suggesting their dual role in stabilizing both the open and closed conformations of WLS, a feature seen in many GPCRs^46^. Notably, in the Wnt-bound state, D353 interacts with the PAM-modified serine, whereas in the apo state, following the movement of TM5, TM7 and the rearrangement of ECL4, D353 is repositioned to form hydrogen bonds with Q454 from TM7 and H459 from ECL4 (Fig. 5h), thus stabilizing the closed conformation of WLS. Additionally, within the transmembrane PAM-binding site, the rotation of TM5 repositions the aromatic rings of F347 and F350 from lining the PAM moiety to sealing the PAM-binding tunnel between TM5 and TM4 (Fig. 5d, e). These structural rearrangements result in the closure of the V-shaped lateral entrance to the central cavity, significantly reducing its volume. Consequently, the DOPC molecule observed in the Wnt-bound open conformation is absent in the apo WLS structure (Fig. 5g, i). While the overall structure of the WLS luminal domain remains largely unchanged between the Wnt-bound and apo states, its Wnt hairpin 3-binding site exhibits weak density in apo WLS (Supplementary Fig. 16d), likely reflecting its dynamic nature in the absence of Wnt binding. Notably, the WLS α1 helix, which forms the binding site for the Wnt hairpin 3 WCC motif, moves outward, adopting an open conformation, to facilitate Wnt release (Fig. 5f).

**Fig. 6:**
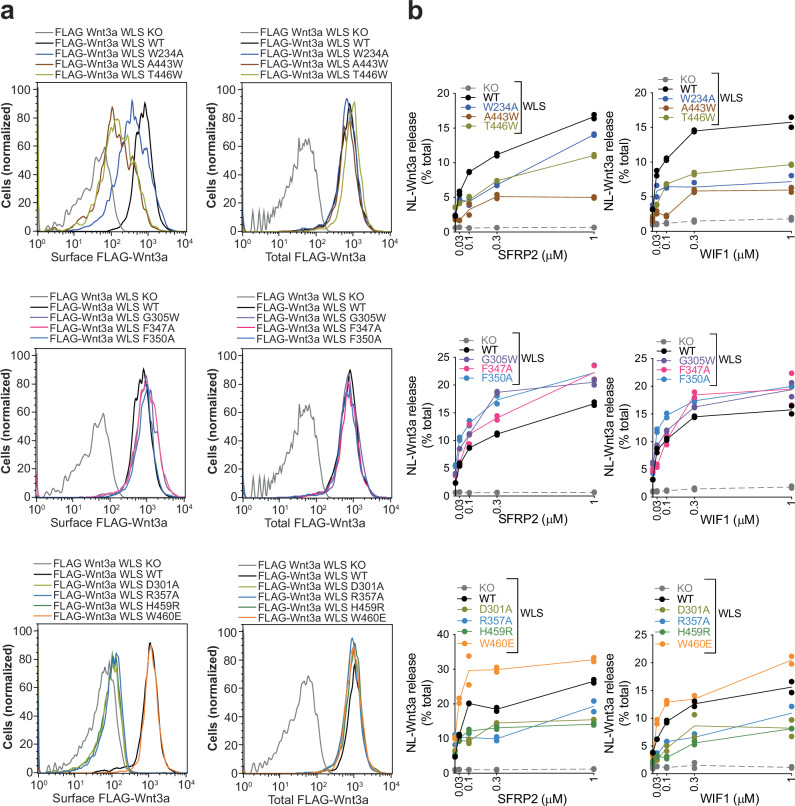
WLS conformational changes are required for Wnt trafficking and secretion. **a** Surface (left, without membrane permeabilization) and total (right, membrane permeabilization) levels of FLAG-mWnt3a in WLS-KO cells stably expressing the indicated WLS constructs. Cells were stained with anti-FLAG-M1-AlexaFluor647 and were analyzed by FACS. **b** WLS-KO sells stably expressing NL–mWnt3a together with the indicated WLS constructs were pre-incubated with cycloheximide. NL-mWnt3a release was initiated by adding varying concentrations of purified SFRP2 or WIF1 in serum-free medium, and was measured by NL luminescence. Data represent the average of two technical replicates (*n* = 2), normalized to total NL–mWnt3a in cell lysates, for a representative experiment. Each experiment has been performed at least twice using distinct biological samples.

Given that TM7 of WLS undergoes the most striking conformational change upon Wnt release, we targeted two residues in this helix, by the mutations A443W and T446W. These substitutions are compatible with the Wnt-bound open conformation of WLS, as they are exposed to the central cavity, but would sterically clash in the closed conformation (Fig. 5i). Both mutations significantly reduced mWnt3a surface localization and carrier-mediated secretion (Fig. 6, top), suggesting that TM7 conformational changes are essential not only for Wnt release but also for loading Wnt onto WLS in the ER, for transport to the cell surface. An alternative explanation is that these mutations may interfere with DOPC binding in the Wnt-bound state (Fig. 5i), which is required to stabilize the open conformation of WLS. Consistent with this idea, mutating W234, which forms a π-cation interaction with the choline head group of DOPC (Fig. 5i), to alanine, also markedly reduced Wnt3a surface localization and secretion (Fig. 6, top). These findings point to the importance of DOPC in stabilizing the Wnt-bound conformation of WLS.

Next, we investigated WLS residues surrounding the Wnt PAM-binding site. Strikingly, the G305W mutation, reported to reduce Wnt secretion due to a predicted steric clash with PAM^34^ (Fig. 5e), did not alter Wnt3a surface localization and actually enhanced its carrier-mediated secretion (Fig. 6, middle). Similarly, the F347A and F350A mutations, which likely facilitate TM5 movement (Fig. 5e), also potentiated Wnt3a secretion (Fig. 6, middle), suggesting that WLS TMD conformational change is critical for Wnt release.

We also examined the role of WLS ECL4 in Wnt transport and release. H459 is fully exposed when ECL4 forms a β-hairpin in the Wnt-bound conformation, but is buried in the cavity upon Wnt release (Fig. 5g, h). The WLS H459R mutation significantly reduced Wnt3a surface localization and carrier-mediated secretion (Fig. 6, bottom), suggesting that arginine may stabilize the closed conformation of WLS through electrostatic interactions with negatively charged residues in the Wnt-binding cavity (Fig. 5h), thus preventing Wnt binding in the ER. In contrast, the W460E mutation did not affect Wnt3a surface localization and instead enhanced its secretion (Fig. 6, bottom); we speculate this is due to destabilizing the structured ECL4 β-strand and facilitating its rearrangement (Fig. 5g), thereby promoting TM7 movement and Wnt release. As control, we tested two reported WLS mutations, D301A and R357A, which disrupt ionic interactions critical for recognition of the PAM-modified serine in Wnt hairpin 2 (Fig. 5h). These mutations markedly reduced Wnt3a surface localization and secretion (Fig. 6, bottom), but they didn’t completely abolish function, as previously reported^34^; this discrepancy suggests that our assays have greater sensitivity compared to those performed without purified Wnt carriers. Collectively, these results point to a previously unrecognized but critical role of ECL4 in regulating WLS conformational changes associated with Wnt binding, transport and release.

Finally, we tested the mWLS mutants above for their role in Wnt5a transport and release by carriers. As shown in Supplementary Fig. 15, all the mWLS mutants had effects on Wnt5a similar to those observed for Wnt3a, indicating that release of both canonical and non-canonical Wnts is driven by the same WLS conformational mechanism.

### Structural implications for the non-canonical Wnt pathway

**Fig. 7:**
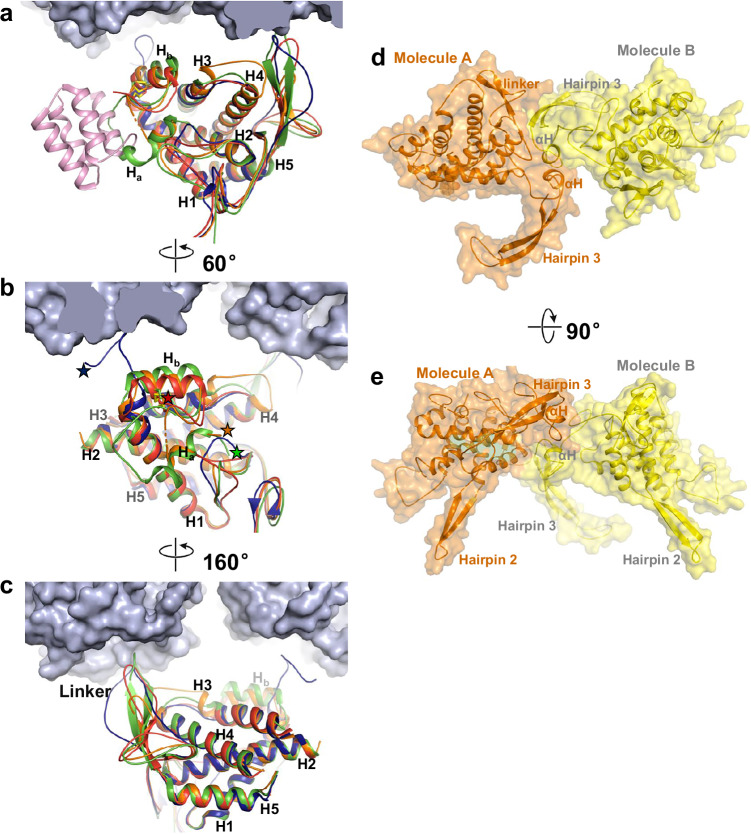
Structural implications for Wnt5a non-canonical function. **a** Superimposition of Wnt5a (orange, our structure), Wnt3a (green, from Wnt3a-WLS complex, PDB ID: 7DRT), Wnt7a-RECK (from Wnt7a-WLS-RECK complex, PDB ID: 8TZP) and engineered Xenopus Wnt8-mFZD8-hLRP6 complex (PDB ID: 8CTG) on the core helical bundle of Wnt NTDs. Wnt7a, RECK, and Wnt8 are shown in red, pink, and blue, respectively. The Wnt main helices are numbered, and the first disulfide bonds are shown as sticks. LRP6 is displayed as a molecular surface (light blue). The Ha helix and subsequent loop of Wnt3a would sterically clash with RECK. The same color scheme is used in (**b**, **c**). **b** As in (**a**), but rotated by 60°. Stars indicate the N-termini of the individual Wnts. While the N-terminus of Wnt8 interacts with LRP6, the N-terminus of Wnt3a is directed away from LRP6 due to the presence of the additional Ha helix and the first disulfide bond, both of which are absent in Wnt8. **c** As in (**b**), but rotated by 160°. The linker domain of Wnts appears to play a crucial role in engaging LRP6. This region in Wnt5a is shorter compared to canonical Wnts, seemingly unable to reach LRP6. **d** Structure of the Wnt5a-WLS 2:2 complex. WLS molecules are omitted for clarity, and the molecular surface is displayed for the Wnt5a homodimer. **e** As in (**d**), but rotated by 90°.

Our Wnt5a–WLS structure sheds light on the molecular basis of Wnt ligand specificity in triggering canonical versus non-canonical signaling, a key unanswered question. The Wnt5a we used for assembling the Wnt5a–WLS complex corresponds to the long isoform, which contains an additional short N-terminal helix (H_a_)^47^. This helix harbors two conserved tandem tryptophan residues found in many Wnts (Supplementary Fig. 11) and packs against a concave hydrophobic site on the surface of the Wnt core structure (Fig. 7a, b). Canonical Wnt3a also possesses this H_a_ helix, which would sterically clash with the Wnt7-specific coreceptor RECK^35,36,48,49^ (Fig. 7a), suggesting that Wnt3a does not bind RECK due to steric hindrance. The H_a_ helix points the N-terminus of Wnts in an orientation opposite to that observed for canonical Wnt8 (Fig. 7b), which lacks both H_a_ and the first disulfide bond (Supplementary Fig. 11). Although the N-terminal fragment of *Xenopus* Wnt8 interacts with LRP6, it has been suggested that the linker region between Wnt NTD and CTD plays the major role in LRP6 engagement^25^ (Fig. 7c). This linker forms a long loop in Wnt8 and Wnt7, and an extended β-hairpin in Wnt3a. In contrast, the corresponding loop in our Wnt5a structure appears too short to reach LRP6 (Fig. 7c). Introducing the Wnt1 linker into Wnt5a converts it into a canonical Wnt, capable of activating β-catenin signaling^25^, supporting a key role for the linker in distinguishing between canonical and non-canonical Wnts. Nevertheless, previous work has shown that the Wnt inhibitory protease Tiki cleaves the N-terminus of Wnt3a^50^. Although the resulting truncated Wnt3a remains capable of binding FZD-CRD, it fails to recruit the LRP6 co-receptor^51^. One possible explanation is that, while the N-terminus of canonical Wnts can adopt two opposite orientations, both may still permit binding to LRP5/6, an interaction required for full activation of the Wnt signaling pathway.

Closer examination of the 2D classification for the 1:1 Wnt5a–WLS particles revealed the occasional presence of an adjacent, blurred particle associated with this complex, suggesting the possible formation of higher-order assemblies. We thus determined the structure of a 2:2 Wnt5a-WLS dimeric complex from an independent dataset collected from a sample prepared by co-expressing N-terminal FLAG-tagged human Wnt5a with WLS. Following affinity purification, the FLAG tag was removed using enterokinase, yielding native Wnt5a, to avoid any artifacts that might be caused by the tag (for the 1:1 complex, the FLAG epitope was attached at the C-terminus of Wnt5a). (Fig. 7d, e, Supplementary Figs. 6, 7, 17). This dimer consists of two nearly identical Wnt5a-WLS monomers, assembled through the asymmetric dimerization of Wnt5a. The dimerization interface is primarily formed by the linker and the adjacent region of the CTD in molecule A, which interacts with the CTD of molecule B, particularly the upper arm region of hairpin 3 (Fig. 7d, e). The total buried accessible surface area at the interface is 1242.1 Å², with contributions of 621.2 Å² from molecule A and 620.9 Å² from molecule B. This dimerization mode has not been observed in canonical Wnt-WLS structures solved to date. Although the current Wnt5a–WLS dimer is unlikely to have physiological relevance, as the TMDs are not in the same membrane plane (Supplementary Fig. 17a, b), it suggests that Wnt5a can homodimerize upon release from WLS; this feature may play an important role in signaling, possibly by inducing FZD dimerization (see Discussion).

## DISCUSSION

Canonical and non-canonical Wnt signaling both play critical roles in a multitude of processes during normal development and physiology, and in disease. Each of the two arms of the Wnt pathway is triggered by specific Wnt ligands. Canonical and non-canonical Wnts share many conserved features, including PAM modification in the ER, WLS-mediated transport to the cell surface, carrier-dependent secretion from producing cells, and PAM-dependent interaction with GPC and FZD proteins on the surface of target cells. However, the mechanisms underlying Wnt ligand specificity remain poorly understood, due at least in part to the lack of structures of non-canonical Wnt, in contrast to several structures available for canonical Wnts. In this study, we present the high-resolution cryo-EM structures of the WLS transporter in complex with the non-canonical ligand, Wnt5a. Similar to canonical Wnts, Wnt5a engages WLS through three conserved hairpin fingers, all of which we demonstrate to be essential for Wnt trafficking to the plasma membrane and for carrier-mediated release from cells; this contrasts with prior reports suggesting that Wnt hairpin 3 is dispensable^34,35^. To elucidate the molecular basis of Wnt release, we also solved the high-resolution cryo-EM structure of WLS in its apo state. A comparison of the Wnt-bound and apo WLS structures revealed that, upon Wnt release, the WLS TMD undergoes conformational transitions that are topologically reminiscent of those that occur during GPCR activation, albeit distinct in detail. These TMD changes are accompanied by previously uncharacterized rearrangements of the WLS extracellular domain, which are essential for coordinating Wnt release. Following Wnt dissociation, WLS adopts a closed conformation, which presumably enables recycling from the plasma membrane and subsequent utilization in another Wnt transport cycle^21,52,53^. Notably, WLS exhibits striking structural homology to GOST family membrane proteins, whose functions remain poorly understood^42,43^. Based on conserved structural features, we speculate that GOST family members may mediate the transport of lipid-modified or membrane-associated cargo within intracellular compartments by cycling between cargo-loaded and apo states, similar to the conformation cycle described here for WLS.

The conformational change in WLS associated with Wnt release is accompanied by dissociation of a phosphatidylcholine (PC) molecule (Fig. 5g, i). Our structure-guided functional studies suggest that the PC molecule stabilizes the Wnt-bound WLS conformation by occupying a hydrophobic cavity that would otherwise collapse. We thus propose that PC plays a structural, rather than a regulatory, role in WLS. This interpretation is supported by several lines of evidence. First, Wnts are not released from cells in the absence of dedicated extracellular carriers, indicating that membrane lipids alone cannot trigger release^22^. Second, while a PC concentration gradient between the ER and plasma membrane has been hypothesized to control Wnt release^36^, it remains unclear if the magnitude of this gradient would suffice to drive PC dissociation from WLS. Furthermore, based on our structure, other positively charged phospholipids, such as sphingomyelin (SM), which is most abundant in the outer leaflet of the plasma membrane^54^, could similarly occupy the cavity in WLS and compensate for the decrease in PC concentration (Supplementary Fig. 8f). Consistently, molecular docking and free energy calculations suggest that WLS binds these two phospholipids similarly. (Supplementary Fig. 8d–h). Finally, Wnt release is robustly triggered only upon addition of extracellular carrier proteins^22^, suggesting that WLS conformational changes must synergize with transient carrier binding to facilitate Wnt handoff.

Unexpectedly, we found that Wnt5a bound to WLS can homodimerize. This finding raises the intriguing possibility that, on the surface of target cells, dimeric Wnt5a may simultaneously engage two copies of its preferred receptors, FZD3 or FZD6^22,55^, and recruit two copies of ROR1/2 co-receptors, to assemble a signaling complex for non-canonical Wnt pathway activation. This model is similarto that of homodimeric Norrin, which binds two copies of FZD4 while recruiting the LRP5/6 co-receptor, to activate canonical β-catenin signaling^56–58^. Notably, prior studies have shown that Wnt5a induces ROR2 homodimerization or ROR1-ROR2 hetero-oligomerization to activate downstream signaling, a mechanism analogous to classical receptor tyrosine kinase (RTK) activation^59–61^. After the final revision of this paper, it was reported that Wnt3a can also dimerize within Wnt3a-FZD8-LRP6 extracellular complexes^62^. Notably, the Wnt3a dimerization mode is similar to that of Wnt5a in our 2:2 Wnt5a-WLS complex (Supplementary Fig. 17g). Future experiments will be required to dissect the role of Wnt dimerization in both canonical and non-canonical Wnt signaling.

## METHODS

### Cell lines

Human embryonic kidney (HEK293) cells were purchased from ATCC, while FreeStyle™ 293 F and Expi293 cells were purchased from Thermo Fisher Scientific. All mammalian cell lines were cultured under standard conditions (37 °C, humidified atmosphere with 5% CO_2_) in DMEM (Corning) supplemented with 10% fetal bovine serum (VWR) and penicillin-streptomycin mix (Corning). For protein expression, FreeStyle™ 293 F and Expi293 cells were grown in suspension in FreeStyle medium (Thermo Fisher Scientific) at 37 °C and 8% CO_2_, with agitation at 125 rpm in vented-cap plastic flasks (Corning). *Spodoptera frugiperda* Sf9 cells (Expression Systems) used to generate BacMam viruses were grown in ESF 921 insect cell medium (Expression Systems), in a shaking incubator at 27 °C.

### Protein expression and purification

Human WLS-Wnt5a complexes were expressed and purified as previously described^22^. Briefly, BacMam viruses encoding codon-optimized full-length hWnt5a, tagged with one copy of the FLAG epitope at the C-terminus (for the 1:1 complex) or at the N-terminus (for the 2:2 complex), and hWLS with a C-terminal human Protein C (HPC) epitope, were generated in Sf9 cells and were used to co-infect FreeStyle™ 293 F cells. Complexes were isolated by tandem-affinity purification using anti-HPC and anti-FLAG M2 affinity resins. For structure determination of the 2:2 Wnt5a-WLS complex, the N-terminal FLAG tag was removed by digestion with enterokinase, which generates Wnt5a with a native N-terminus. Samples were further purified by size-exclusion chromatography (SEC) on a Superose 6 column (Increase 10/300 GL, Cytiva), in gel filtration buffer [20 mM HEPES, pH 7.5; 150 mM NaCl; 0.02% (w/v) GDN (Anatrace)].

To purify unliganded WLS, the BacMam virus encoding hWLS was used to infect FreeStyle™ 293 F cells. The protein was purified by anti-HPC affinity^22^, followed by SEC on a Superose 6 Increase 10/300 GL column in DDM buffer [20 mM HEPES, pH 7.5; 150 mM NaCl; 0.03% (w/v) DDM (Anatrace); 0.003% (w/v) cholesteryl hemisuccinate (CHS, Anatrace)]. For nanodisc reconstitution, purified WLS was mixed with purified recombinant MSP1D1 and a cholate-solubilized stock of soybean polar lipid extract (Avanti Research) supplemented with 2.5% (w/w) cholesterol (Sigma) at a molar ratio of 1:3:225. After incubation on ice for 1 h, SM-2 BioBeads (Bio-Rad) were added, and the mixture was rotated overnight at 4 °C to facilitate nanodisc assembly through detergent removal. BioBeads were then removed, and 5 mM CaCl_2_ was added to the sample. To eliminate empty nanodiscs, WLS was recaptured on anti-HPC resin followed by extensive washes with binding buffer (20 mM HEPES, pH 7.5; 150 mM NaCl; and 2 mM CaCl_2_). WLS-HPC-containing nanodiscs were eluted in elution buffer (20 mM HEPES, pH 7.5; 150 mM NaCl; 0.1 mg/mL HPC peptide; 10 mM EDTA), and further purified on a Superose 6 Increase 10/300 GL column in binding buffer without CaCl_2_. The size-exclusion chromatograms and SDS-PAGE analyses of final protein samples are shown in Supplementary Fig. 1. Wnt carrier proteins used in this study were expressed and purified as previously described^22^.

### Cryo-EM sample preparation and data collection

To prepare cryo-EM samples, gel filtration peak fractions containing WLS-Wnt5a complexes or apo WLS were pooled and concentrated to 5–10 mg/mL. A 1 μL aliquot of each sample was applied onto a glow-discharged holey carbon grid (Quantifoil 300 mesh, Cu R1.2/1.3), and blotted for 5 s at 4 °C and 100% relative humidity using a FEI Vitrobot Mark IV plunger before plunge-freezing.

Cryo-EM data for apo WLS and the WLS-Wnt5a 2:2 complex were collected at the Laboratory for Biomolecular Structure and Dynamics (LBSD) of Texas A&M University using a Titan Krios G4 electron microscope (Thermo Fisher Scientific), operated at 300 kV and a nominal magnification of 105,000×. Micrographs were recorded using a BioContinuum imaging energy filter (Gatan) with a slit width of 15 eV and a K3 direct electron detection camera (Gatan) in counted super-resolution mode. The dose rate was set to 13.534 e^−^/pixel/second, resulting in a total accumulated dose of 50.83 e^−^/Å². Each micrograph had a total exposure time of 1.3 s, divided into 40 frames with an individual frame exposure time of 0.0325 s. Data collection was performed using the EPU software (version 3.4.0, Thermo Fisher Scientific) in AFIS mode, with a defocus range of −0.8 μm to −2.4 μm. Similar procedures were used to collect the cryo-EM dataset for the WLS-Wnt5a 1:1 complex at the Cryo-EM Core Facility at UMass Chan Medical School.

### Cryo-EM image processing

The data processing workflows for the cryo-EM datasets of the WLS-Wnt5a 1:1 complex, apo WLS, and WLS-Wnt5a 2:2 complex are summarized in Supplementary Figs. 2–7, respectively. Unless otherwise specified, the data processing method was consistent across all three datasets. Beam-induced image motion in dose-fractionated movies was corrected using MotionCor2^63^, with motion correction performed at 2 × binning, to achieve a target pixel size of 0.83 Å for the WLS-Wnt5a 1:1 complex and 0.82 Å for apo WLS and the WLS-Wnt5a 2:2 complex. Defocus values and astigmatism for each motion-corrected micrograph were determined using CTFFIND4^64^. Micrographs with a resolution better than 4 Å, as indicated by CTFFIND4, were selected for further processing. Particle picking was carried out using Topaz^65^, and data processing was performed in RELION4^66^. The final 3D reconstructions of the WLS-Wnt5a 1:1 complex and apo WLS were refined using non-uniform 3D refinement in cryoSPARC^67^.

For the apo WLS and WLS-Wnt5a 1:1 complex datasets, particles picked using Topaz were extracted from motion-corrected micrographs at 2× binning into 96 × 96 pixels (apo WLS) or 128 × 128 pixels (WLS-Wnt5a 1:1 complex) for multiple rounds of 2D classification and selection. The selected particles were then used to generate ab initio 3D reconstructions in RELION. After an initial round of 3D classification, particles from the best class were re-extracted at 256 × 256 pixels without binning, for further processing. To refine the dataset, iterative rounds of 2D and 3D classification were performed until a highly homogeneous particle subset was obtained for 3D auto-refinement. The composite masking method^68,69^, incorporated in a modified version of RELION4 (see https://github.com/jiangjiansen/relion_composite_masks), was used for 3D classification and auto-refinement. Typically, a composite mask consisted of a standard reference mask (magenta in Supplementary Figs. 2, 4, 6) covering only the protein, and a second mask (gray in Supplementary Figs. 2, 4, 6) covering the entire particle, including the detergent micelle or lipid nanodisc. CTF refinement and particle polishing were performed before the final high-resolution 3D reconstruction. The final particle subsets were transferred to cryoSPARC for further processing using non-uniform 3D refinement, yielding the cryo-EM 3D reconstructions used for atomic model building and refinement.

During the data processing of the WLS-Wnt5a 1:1 complex, an additional 3D classification step focusing on the extracellular region was performed after particle polishing (Supplementary Fig. 2). From this step, 104,884 out of 160,832 particles were selected, resulting in a 3D reconstruction with enhanced map quality in the extracellular region, without compromising the resolution of other regions.

For the WLS-Wnt5a 2:2 complex dataset, particles picked using Topaz were initially extracted at 2× binning into 128 × 128 pixels from motion-corrected micrographs. After multiple rounds of 2D classification and particle selection, 3D refinement was performed using the ab initio 3D reconstruction of the WLS-Wnt5a 1:1 complex as the starting model. Based on the 3D refinement results, the particles were re-centered and re-extracted at 2× binning into 160 × 160 pixels. A subsequent 2D classification was conducted to select WLS-Wnt5a 2:2 dimeric complexes (Supplementary Fig. 6b). A composite mask covering the additional densities beyond the WLS-Wnt5a 1:1 complex was applied during 3D classification, revealing two distinct conformations: complex A and complex B. Particles from these two classes were then re-extracted at full resolution into 400×400 pixels without binning for final 3D refinements. Complex A and complex B were found to be complementary, together forming the WLS-Wnt5a 2:2 dimeric complex. To reconstruct the full WLS-Wnt5a 2:2 dimeric complex (complex A/B), particles from either complex A or B were refined using a composite mask that encompassed both conformations. However, due to the flexibility within the WLS-Wnt5a 2:2 dimeric complex, the resolution of complex A/B was limited. To address this, a composite density map of complex A/B was generated for atomic model building by merging the aligned maps of complex A and complex B using the “*volume maximum*” command in UCSF ChimeraX^70^ (Supplementary Fig. 7d).

### Atomic model building and refinement

For the apo WLS structure, the AlphaFold-predicted model of human WLS^71^ was fitted into the cryo-EM map using the *dock_in_map* function in PHENIX^72^. For the WLS-Wnt5a 1:1 complex, the previously determined human WLS-Wnt3a structure^35^ (PDB ID: 7DRT) served as a template. The resulting models were manually rebuilt in Coot^73^ and further refined in PHENIX using real-space refinement^72^. For the WLS-Wnt5a 2:2 complex, the monomeric WLS-Wnt5a structure was used to search for two copies within the final composite map, followed by rigid body refinement in Coot^73^. All structural figures were generated in PyMOL. Data collection and model refinement statistics are summarized in Supplementary Table 1.

### Molecular docking and binding free energy estimation

The cryo-EM structure of the 1:1 WLS–Wnt5a complex was prepared using the Protein Preparation Wizard in the Schrödinger Suite (Release 2025-1: Maestro, Schrödinger, LLC, New York, NY, USA, 2025). Missing hydrogen atoms were added, bond orders were assigned, and the hydrogen-bonding network was optimized at pH 7.4. The structure was subjected to restrained energy minimization using the OPLS4^74^ force field until the heavy-atom root-mean-square deviation (RMSD) converged to 0.30 Å. The ligands 1,2-dioleoyl-sn-glycero-3-phosphocholine (DOPC) and sphingomyelin (SM) were prepared using LigPrep, and ionization states were assigned using Epik^75^ at pH 7.4 ± 2.0. A receptor grid was defined in Glide as a 10 Å cubic box centered on the centroids of the manually built DOPC and SM molecules from the cryo-EM map, which served as reference ligands. Docking was performed in Glide^76,77^ using Ligand Alignment mode with Maximum Common Substructure (MCS) constraints, to preserve the native binding orientation of the lipid scaffold. Conserved structural cores of the lipid analogs were aligned to the reference ligands, while acyl chains were sampled flexibly in Standard Precision mode. Final poses were evaluated using the “score in place only” option without additional sampling of the aligned core. The binding free energies (ΔG_bind_) were estimated using the Prime MM-GBSA module. To account for induced-fit effects, the ligand and all receptor residues within 5 Å of the binding site were locally optimized. Ligand strain energy was also included to ensure realistic binding conformations.

### Generation of stable cell lines

Stable cell lines were established by lentiviral transduction, as described previously^22^. Briefly, HEK293T cells were transduced with lentiviral vectors for expressing FLAG-tagged mWnt3a and Cas9, followed by selection with Hygromycin B and Blasticidin S, respectively, and single cell cloning. A clone with uniform FLAG-Wnt3a and Cas9 expression was used for subsequent experiments.

### Generation of WLS- and PORCN-knockout cells

To generate WLS or PORCN knockout (KO) cells, clonal FLAG-Wnt3a/Cas9-expressing cells were transiently transfected with sgRNAs targeting human WLS (TGGCCCATGAAAGAGTACCA) or PORCN (GATCCCTACAGAGCTGCCGG). After selection with puromycin, single-cell cloning was performed on day 7. The clones were screened by flow cytometry for the disappearance of cell surface FLAG-mWnt3a expression. Reversal of the KO phenotype was confirmed by lentiviral complementation with the tagged respective gene (WLS or PORCN), followed by FACS analysis. Loss of WLS or PORCN expression in the knockout clones was confirmed by immunoblotting for WLS and PORCN, respectively.

### Antibodies

The following antibodies were obtained commercially: rabbit monoclonals against Wnt3a and Vinculin (Cell Signaling Technology); mouse monoclonal anti-WLS (BioLegend); rabbit polyclonal anti-PORCN (Novus Biologicals); and mouse monoclonal anti-α-tubulin (Santa Cruz Biotechnology). Mouse anti-FLAG-M1 and anti-HPC monoclonal antibodies were kindly provided by Andrew C. Kruse (Harvard Medical School). For immunoblotting, primary antibodies were diluted to 1–2 µg/mL in TBST [10 mM Tris-HCl (pH 7.6), 150 mM NaCl, 0.2% Triton X-100 (v/v)] with 5% (w/v) non-fat dry milk. For immunoblots using anti-FLAG-M1 and anti-HPC antibodies, all buffers were supplemented with 2 mM CaCl_2_. HRP-conjugated secondary antibodies were used at 0.2 µg/mL and were sheep anti-mouse IgG–HRP (Jackson ImmunoResearch) and donkey anti-rabbit IgG–HRP (GE Healthcare). For flow cytometry, AlexaFluor647-conjugated anti-FLAG-M1 antibody was used at 1 µg/mL in PBS supplemented with 2% FBS, 2 mM CaCl_2_ and 10 mM HEPES.

### Western blotting

Proteins were separated on 4–20% gradient SDS-PAGE gels (Mini-PROTEAN TGX, Bio-Rad). Following electrophoresis, the gels were incubated in transfer buffer [48 mM Tris (pH 9.2), 39 mM glycine, 1.3 mM SDS, and 20% (v/v) methanol], then blotted onto nitrocellulose membranes (Millipore) using a semi-dry blotting apparatus (Trans-Blot SD, Bio-Rad). Membranes were blocked in TBST with 5% non-fat dry milk, incubated with primary antibodies overnight at 4 °C, and washed thrice in TBST. Incubation with HRP-conjugated secondary antibody was carried out in TBST with 5% non-fat dry milk for 1 h at room temperature. The blots were washed thrice with TBST and twice with TBS, followed by chemiluminescence imaging.

### Flow cytometry

HEK293T cells expressing FLAG-mWnt3a or FLAG-hWnt5a constructs were dissociated using enzyme-free cell dissociation solution (Sigma Aldrich), washed and incubated for 20 min on ice with AlexaFluor647-conjugated anti-FLAG antibody in staining buffer (PBS with 2% FBS, 2 mM CaCl_2_ and 10 mM HEPES), with or without Triton X-100 permeabilization, for detecting total and cell surface Wnt levels, respectively. After two washes with staining buffer, cells were analyzed using an Attune flow cytometer (Thermo-Fisher). Data was analyzed using the FlowJo software (BD Biosciences). A representative FACS gating strategy used throughout this work is shown in Supplementary Fig. 13c. All FACS experiments were repeated at least twice using distinct biological samples.

### Wnt release assays

HEK293 cells stably expressing mWnt3a or hWnt5a (WT and mutants) were rinsed thrice with serum-free DMEM and were then cultured for 24 h in serum-free DMEM supplemented with various purified carriers. Levels of Wnt in the conditioned media and corresponding cell lysates were analyzed by SDS-PAGE followed by immunoblotting. For NanoLuc (NL)-based Wnt release assays, HEK293 cells stably expressing NL-fused mWnt3a or hWnt5a constructs were maintained overnight in serum-free DMEM before addition of purified test proteins. At specified times, conditioned media were collected in duplicate, cleared by centrifugation, and NL activity in the supernatant was measured using Nano-Glo Luciferase Assay Substrate (Promega), according to the manufacturer’s instructions. NL activity in the media was normalized to the total NL measured in cell lysates harvested at the end of the assay. All Wnt release experiments were repeated at least twice using distinct biological samples.

### Wnt reporter assays

Wnt signaling activity of conditioned media was measured using a MEF cell line stably expressing the pBAR reporter system, which consists of firefly luciferase driven by TCF-responsive elements and constitutively-expressed Renilla luciferase (for normalization). MEF pBAR cells were plated into 96-well plates and were then treated in duplicate for 24 h with conditioned media diluted in serum-free DMEM. Luminescence was measured and firefly/Renilla ratios were calculated as previously described^22^. All Wnt reporter experiments were repeated at least twice using distinct biological samples.

### LC-MS/MS analysis of Wnt lipidation

Human Expi293 cells stably expressing N-terminal FLAG-tagged mouse Wnt3a (WT or mutant) were harvested from 0.5-liter suspension cultures. Cell pellets were solubilized in lysis buffer (20 mM HEPES, pH 7.5; 150 mM NaCl; 2 mM CaCl_2_; 0.2% Triton X-100), supplemented with protease inhibitor cocktail tablets (Roche). The lysates were subjected to centrifugation at 20,000 *g* and 4 °C, after which clarified supernatants were loaded onto 0.1 mL of anti-FLAG M1 antibody beads. The beads were washed extensively with lysis buffer, three times with detergent-free buffer (20 mM HEPES, pH 7.5; 150 mM NaCl; 2 mM CaCl_2_), sucked dry and stored at −80 °C until processing for mass spectrometry.

For LC-MS/MS analysis of Wnt lipidation, FLAG-tagged WT mWnt3a samples immunopurified from WT and PORCN-KO cells were used as positive and negative controls, respectively. One biological replicate was analyzed for each Wnt3a mutant and control sample, with one technical replicate performed for each. For mass spec sample preparation, the beads were resuspended in 100 μL of 50 mM Tris-HCl, 10 mM CaCl_2_ and 2 M urea. To reduce disulfide bonds, 8 μL of 0.5 M tris (2-carboxyethyl) phosphine hydrochloride (TCEP, Sigma-Aldrich) was added, and the beads were incubated at room temperature for 30 min. Cysteine alkylation was subsequently performed by adding 8 μL of 0.5 M iodoacetamide (IAA, Sigma–Aldrich), followed by incubation for 30 min at room temperature in the dark. The supernatant was removed, and 2 μg of trypsin (Pierce) in 100 μL of 50 mM Tris-HCl and 10% acetonitrile (ACN) was added, and the sample was incubated overnight at 37 °C. The digestion was quenched with 10 μL of 10% formic acid, and the supernatant was transferred into a new tube. The beads were further incubated with 200 μL of 0.1% formic acid for 5 min, and the resulting supernatant was combined with the previous one. The sample was then diluted with an additional 200 μL of 0.1% formic acid, and solid phase extraction was performed using a 96-well plate with C8 resin (Bond Elut, Agilent Technologies, A4960325). Samples were eluted with 400 μL of 0.1% formic acid and 80% ACN, and were dried in a SpeedVac (Thermo). The dried peptides were reconstituted in 21 μL of 0.1% formic acid and 2% ACN, and were centrifuged at 20,000 *g* for 3 min. 10 μL from the top of the solution was transferred to a vial for MS analysis.

Samples were analyzed on a Q Exactive HF mass spectrometer (Thermo), coupled to a Vanquish Neo nano liquid chromatography (LC) system (Thermo). Samples were injected onto a 75 μm i.d. and 15 cm-long EasySpray column (Thermo Fisher Scientific), and were eluted with a 0–28% gradient buffer B over 90 min. Buffer A contained 2% (v/v) ACN and 0.1% formic acid in water, and buffer B contained 80% (v/v) ACN, 10% (v/v) trifluoroethanol and 0.1% formic acid in water. The mass spectrometer operated in positive ion mode with a source voltage of 2.5 kV and an ion transfer tube temperature of 300 °C. MS scans were acquired at 120,000 resolution in the Orbitrap, and up to 20 MS/MS spectra were obtained in the ion trap for each full spectrum acquired using higher-energy collisional dissociation (HCD) for ions with charges 2–8. Dynamic exclusion was set for 20 s after an ion was selected for fragmentation.

Raw MS data files were analyzed using Proteome Discoverer v.3.0 SP1 (Thermo Fisher Scientific), with peptide identification performed using a trypsin digest search with Sequest HT (cleavage after Lys and Arg, except when followed by Pro). The human reviewed protein database from UniProt (downloaded Dec. 31, 2025, 20354 sequences) was used along with the mWnt3a WT and mutant sequences. Precursor and fragment tolerances of 10 ppm and 0.02 Da were specified, and up to three missed cleavages were allowed. A minimum peptide length of 6 residues was required, and at least 2 unique peptides were required for positive protein identification. Carbamidomethylation of Cys (+57.0215) was set as a fixed modification, with oxidation of Met (+15.9949) and palmitoleoylation of Ser (+236.2140) set as variable modifications. The false-discovery rate (FDR) cutoff was 1% for all peptides and proteins.

### Quantification and statistical analysis

Details of statistical analyses for quantitative experiments are provided in the corresponding figure legends. Where applicable, Wnt signaling activity was normalized to untreated or BSA-treated controls, as described. All functional assays were independently repeated on at least two separate occasions, using different reagent preparations when possible.

### Reporting summary

Further information on research design is available in the Nature Portfolio Reporting Summary linked to this article.

## Supplementary information

Supplementary Information

Description of Additional Supplementary Files

Supplementary Video 1

Supplementary Video 2

Reporting Summary

Transparent Peer Review file

## Source data

Source Data

## Data Availability

The cryo-EM density maps of apo WLS, the Wnt5a-WLS 1:1 complex, and the composite map of the Wnt5a-WLS 2:2 complex have been deposited in the Electron Microscopy Data Bank (EMDB) under accession codes EMD-73810, EMD-73803 and EMD-73838, respectively. For the Wnt5a-WLS 2:2 complex, additional focused maps for Complex A, Complex B, and the consensus map have been deposited under accession codes EMD-73835, EMD-73836 and EMD-73837, respectively. The corresponding atomic coordinates of these structures have been deposited in the Protein Data Bank (PDB) under accession codes 9Z4O for apo WLS, 9Z4H for the Wnt5a-WLS 1:1 complex, and 9Z67 for the Wnt5a-WLS 2:2 complex, respectively. Mass spectrometry data generated in this study have been deposited in MassIVE under accession number MSV000101445. All other data are available in the main text or the supplementary materials. Source data are provided within the Source Data File. Source data are provided with this paper.
