# Supplementary material for: Conformational cycling of the Wntless transporter drives trafficking and secretion of Wnt morphogens
Source: Nat Commun. 2026 Jun 19;17:7737. doi: 10.1038/s41467-026-74521-y (PMC13434646; doi:10.1038/s41467-026-74521-y)
Supplement: Supplementary file 1 [file 41467_2026_74521_MOESM1_ESM.pdf]

Supplementary Information

Conformational cycling of the Wntless transporter drives trafficking and secretion of Wnt morphogens

Yunhui Ge[1,*,#], Taciani de Almeida Magalhaes[2,*,#], Hongjiang Wu[3,*], Dick J. H. van den Boomen[2], Thu Uyen Nguyen[1], Tongyi Dou[4], Gaya P. Yadav[5], Sukyeong Lee[3,6], Zhao Wang[3], Andrew Lemoff[7], Xuemei Luo[7], Sumitha S. Menon[2,8], Min Zhang[3], Jin Wang[3], Zhicheng Jin[9,10], Jiansen Jiang[4,#], Adrian Salic[2,#], Pengxiang Huang[1,#]

[1] Department of Molecular and Cellular Biology, Baylor College of Medicine, Houston, TX 77030, USA
[2] Department of Cell Biology, Blavatnik Institute, Harvard Medical School, Boston, MA 02115, USA
[3] Verna and Marrs McLean Department of Biochemistry and Molecular Pharmacology, Baylor College of Medicine, Houston, TX 77030, USA
[4] Laboratory of Membrane Proteins and Structural Biology, Biochemistry and Biophysics Center, National Heart, Lung, and Blood Institute, National Institutes of Health, Bethesda, MD 20892, USA
[5] Laboratory for Biomolecular Structure and Dynamics (LBSD), Department of Biochemistry and Biophysics, Texas A&M University, College Station, TX 77843, USA
[6] Advanced Technology Core for Macromolecular X-ray Crystallography, Baylor College of Medicine, Houston, TX 77030, USA
[7] Department of Biochemistry, University of Texas Southwestern Medical Center, Dallas, TX 75390, USA
[8] Massachusetts College of Pharmacy and Health Sciences, Boston, MA 02115, USA
[9] University of Wisconsin Hospital and Clinics, Madison, WI 53792, USA
[10] Department of Pathology and Laboratory Medicine, University of Wisconsin-Madison, Madison, WI 53792, USA

* These authors contributed equally.
[#] Correspondence: yunhui.ge@bcm.edu, taciani_magalhaes@hms.harvard.edu, jiansen.jiang@nih.gov, adrian_salic@hms.harvard.edu, pengxiang.huang@bcm.edu

Supplementary Figures

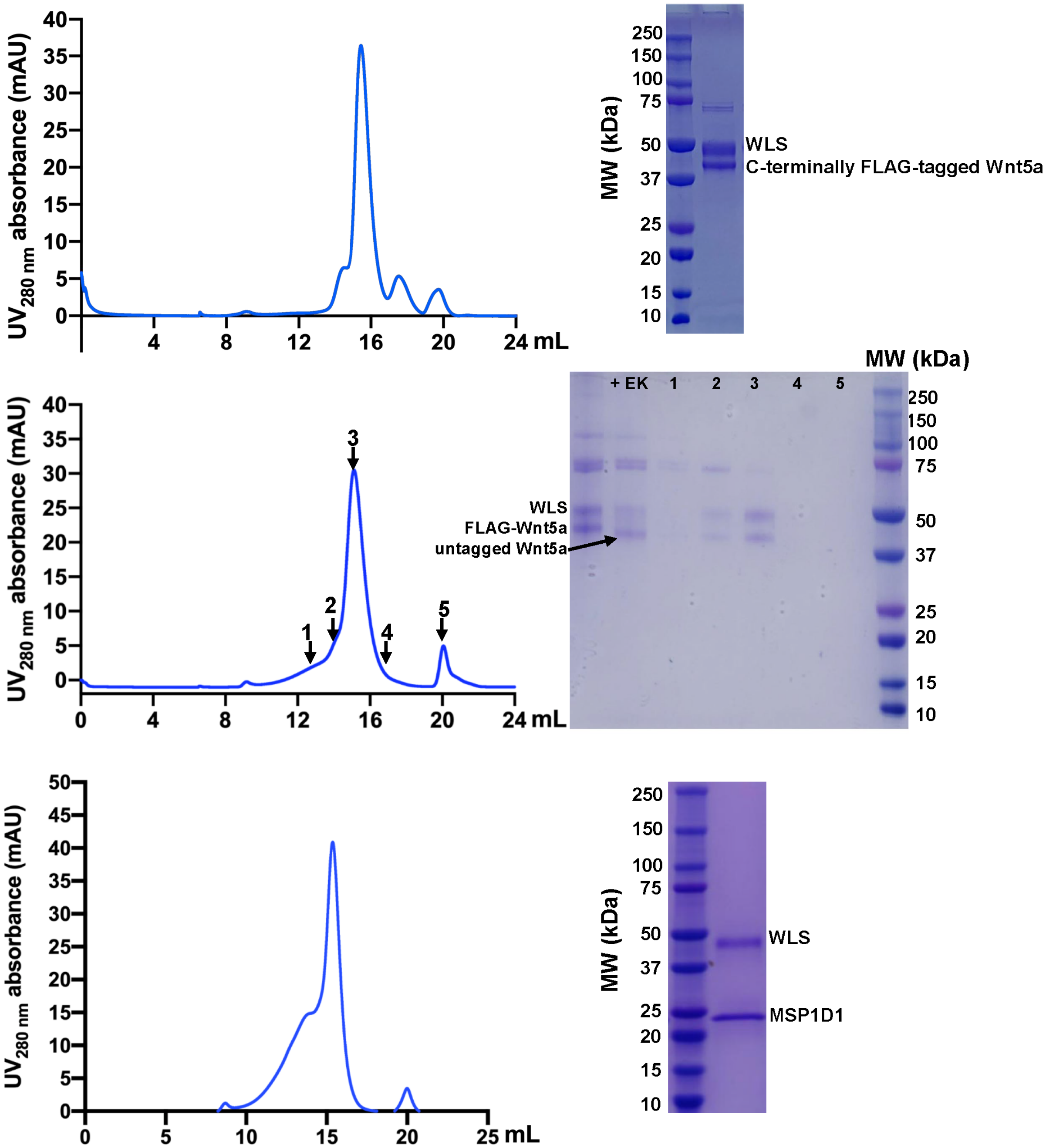

Supplementary Fig. 1: Purified protein samples used for cryo-EM

Size-exclusion chromatograms (Superose® 6 Increase 10/300 GL) of purified human WLS-Wnt5a (C-terminally FLAG-tagged) complex in GDN detergent (top), FLAG tag-removed WLS-Wnt5a in GDN (middle), and apo WLS in nanodiscs (bottom), analyzed by SDS-PAGE and Coomassie staining. Middle SDS–PAGE: lanes from left to right show WLS–Wnt5a (N-terminally FLAG-tagged), FLAG tag removed by enterokinase (EK), gel filtration fractions 1–5 (as indicated in the chromatogram), and a protein molecular weight marker.

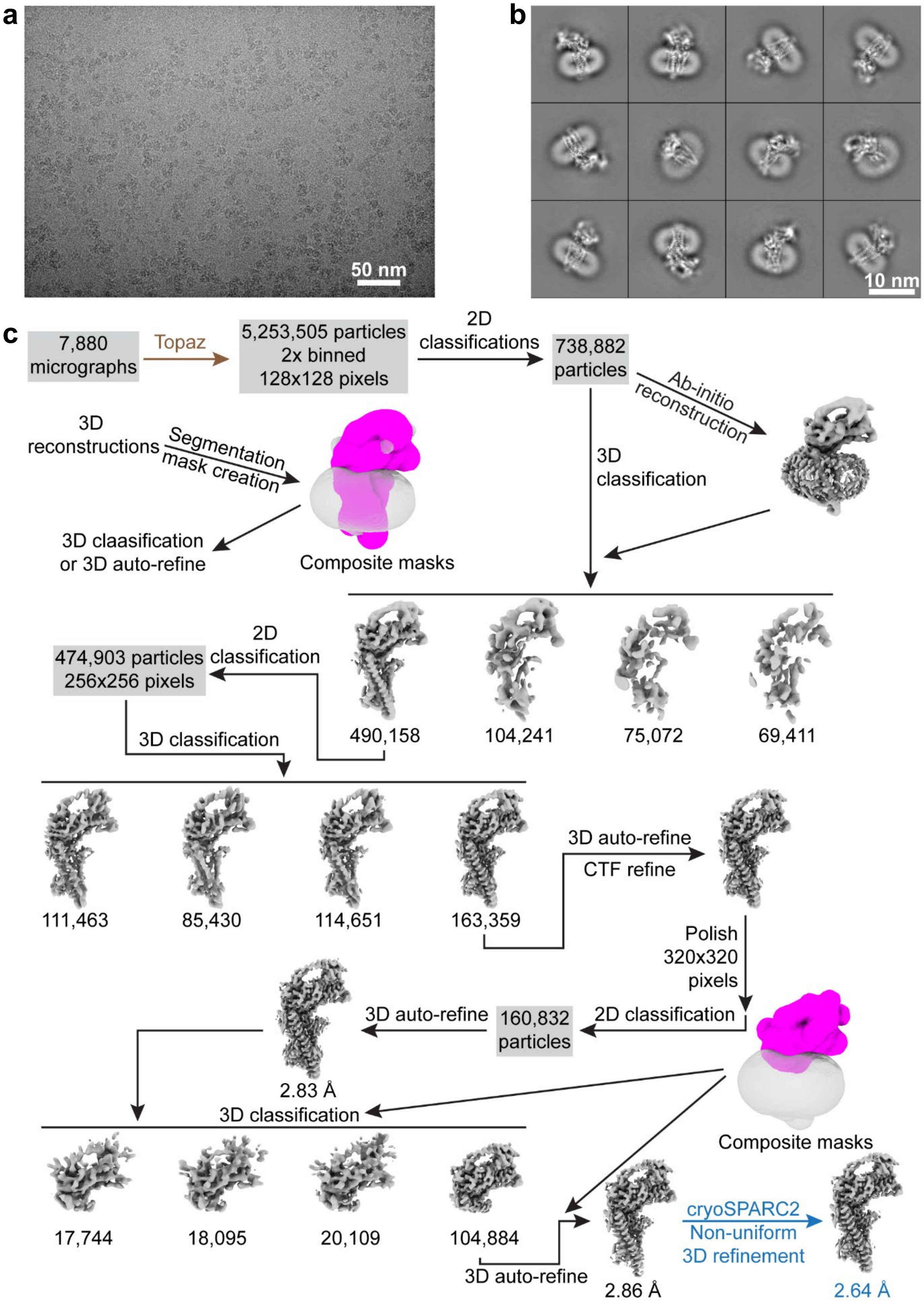

a
50 nm
b
10 nm
c
7,880 micrographs
Topaz
5,253,505 particles
2x binned
128x128 pixels
2D classifications
738,882 particles
Ab-initio reconstruction
3D classification
3D reconstructions
Segmentation mask creation
Composite masks
3D claasification or 3D auto-refine
490,158
104,241
75,072
69,411
2D classification
474,903 particles
256x256 pixels
3D classification
111,463
85,430
114,651
163,359
3D auto-refine
CTF refine
Polish
320x320 pixels
2D classification
160,832 particles
3D auto-refine
2.83 Å
3D classification
Composite masks
17,744
18,095
20,109
104,884
3D auto-refine
2.86 Å
cryoSPARC2
Non-uniform
3D refinement
2.64 Å

Supplementary Fig. 2: Cryo-EM data processing for the human Wnt5a-WLS complex

(a) Representative cryo-EM micrograph for the Wnt5a-WLS complex sample.

(b) Representative 2D class averages of Wnt5a-WLS particles.

(c) Cryo-EM data processing workflow.

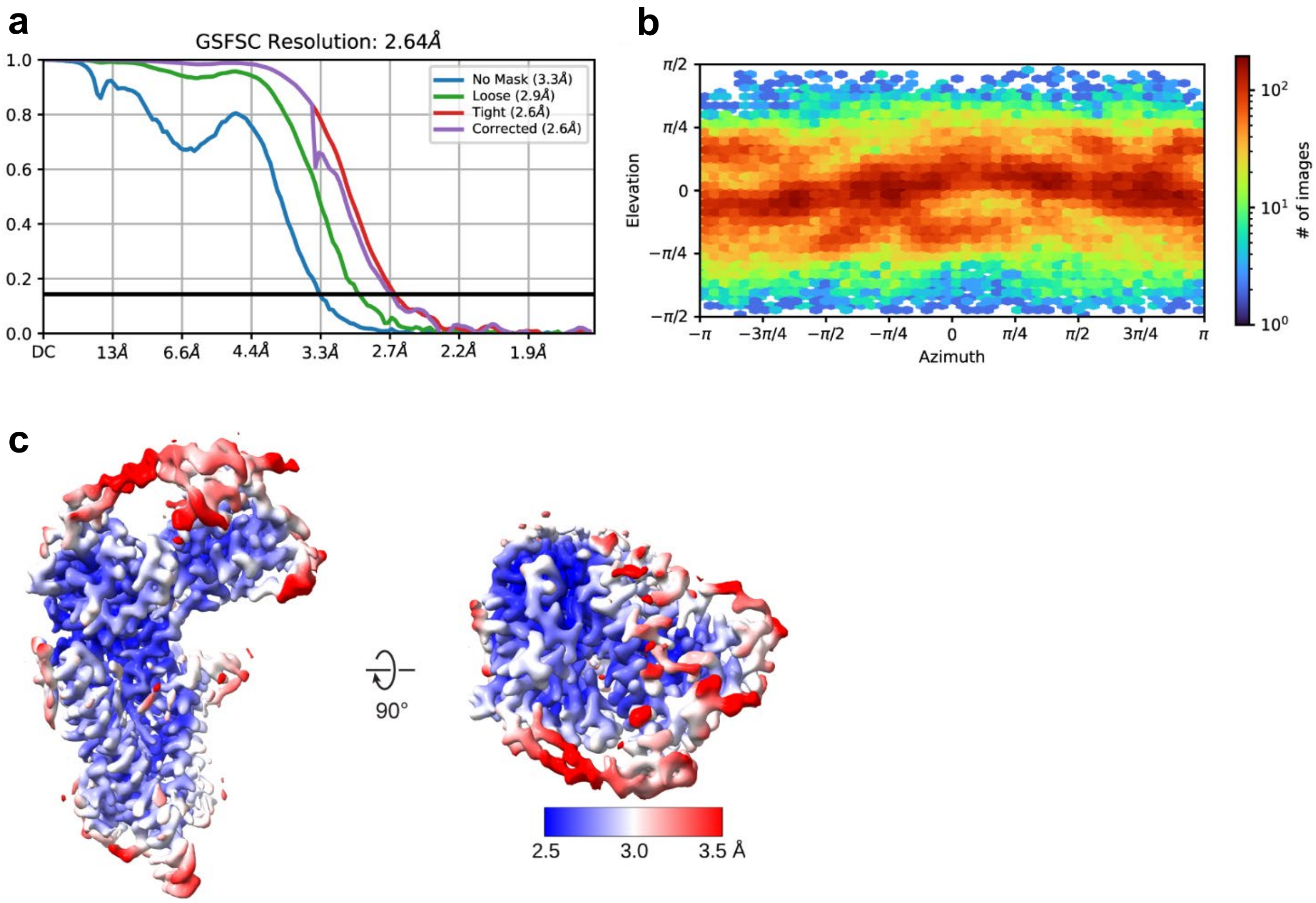

Supplementary Fig. 3: Resolution estimation of cryo-EM 3D reconstruction of the human Wnt5a-WLS complex

(a) Gold-standard FSC curve.

(b) Particle orientation plot.

(c) Local resolution map.

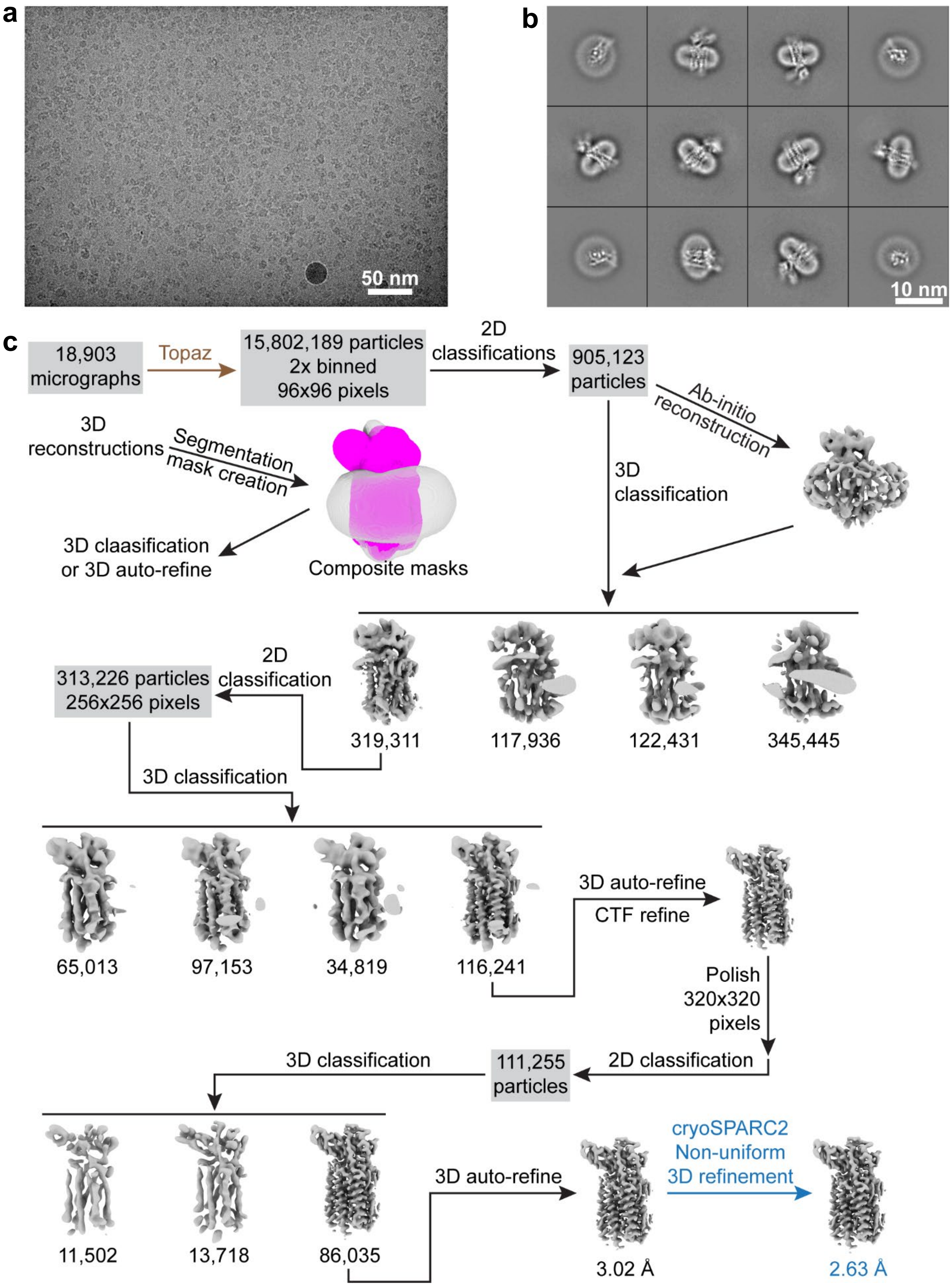

a
50 nm
b
10 nm
c
18,903 micrographs
Topaz
15,802,189 particles
2x binned
96x96 pixels
2D classifications
905,123 particles
Ab-initio reconstruction
3D classification
3D reconstructions
Segmentation mask creation
Composite masks
3D claasification or 3D auto-refine
319,311
117,936
122,431
345,445
2D classification
313,226 particles
256x256 pixels
3D classification
65,013
97,153
34,819
116,241
3D auto-refine
CTF refine
Polish
320x320 pixels
2D classification
111,255 particles
3D classification
11,502
13,718
86,035
3D auto-refine
3.02 Å
cryoSPARC2
Non-uniform
3D refinement
2.63 Å

Supplementary Fig. 4: Cryo-EM data processing for apo human WLS in lipid nanodiscs
(a) Representative cryo-EM micrograph for the apo WLS sample.
(b) Representative 2D class averages for apo WLS particles.
(c) Cryo-EM data processing workflow.

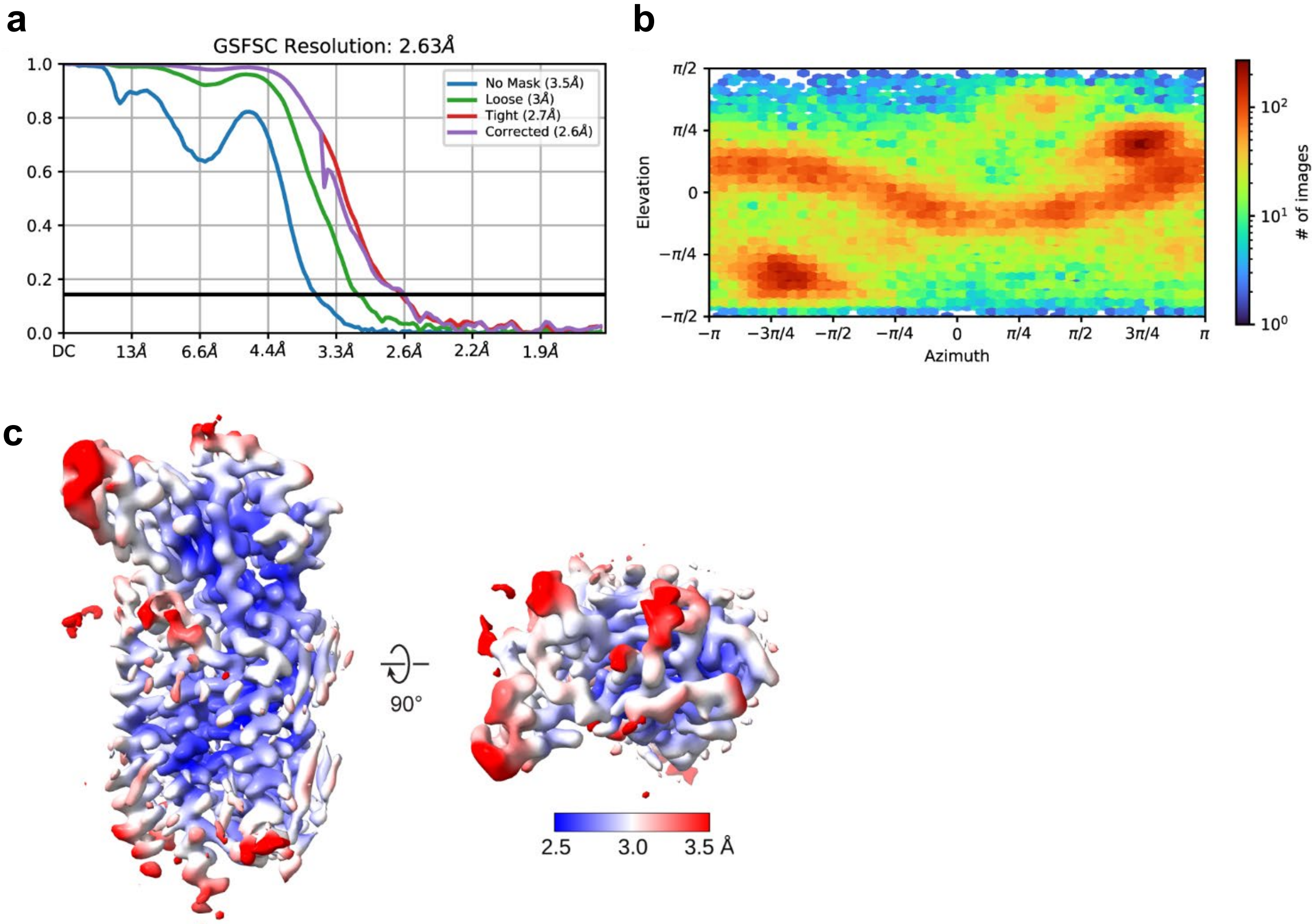

Supplementary Fig. 5: Resolution estimation of cryo-EM 3D reconstruction of apo WLS

(a) Gold-standard FSC curve.

(b) Particle orientation plot.

(c) Local resolution map.

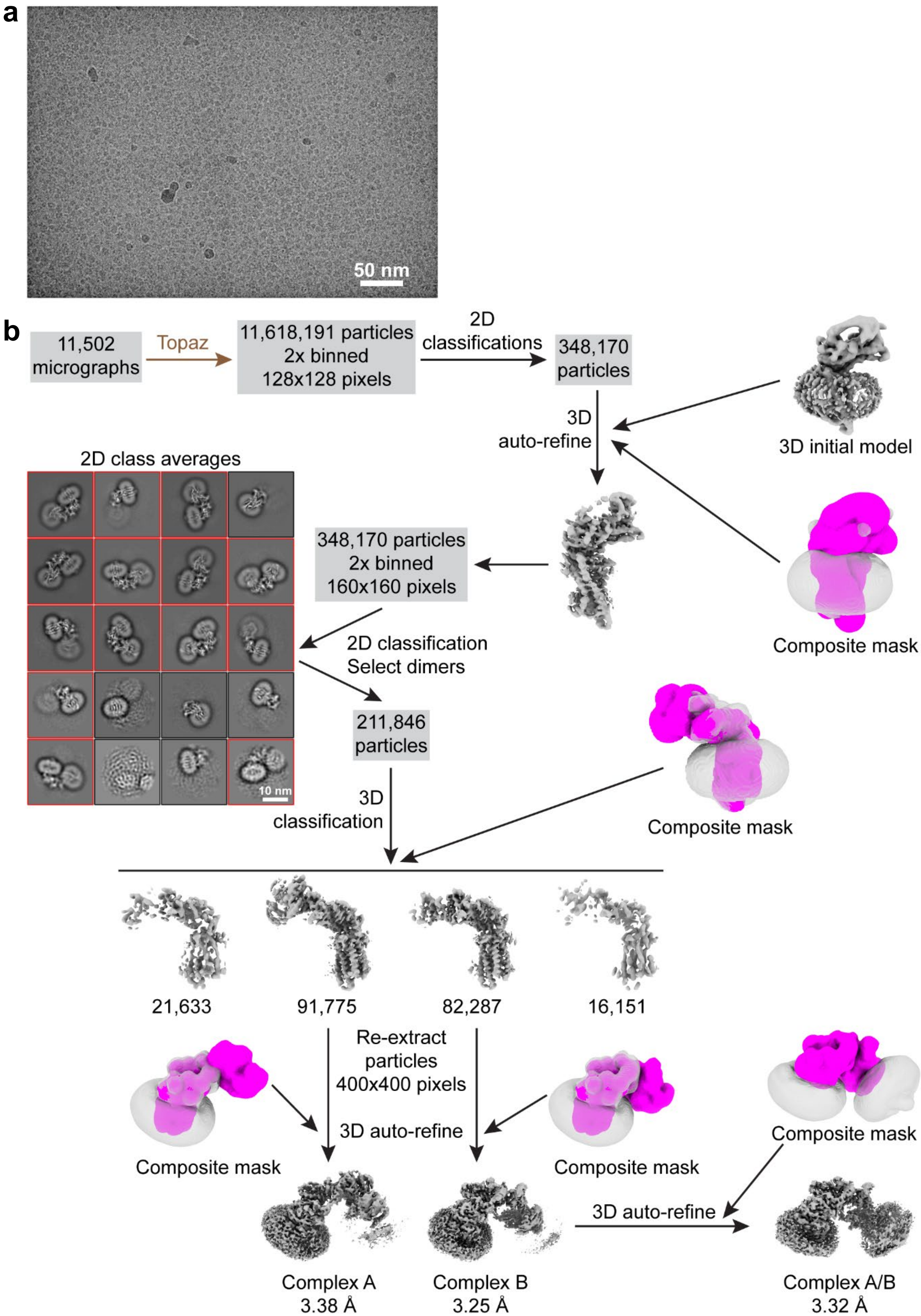
a
50 nm
b
11,502 micrographs
Topaz
11,618,191 particles
2x binned
128x128 pixels
2D classifications
348,170 particles
3D auto-refine
3D initial model
2D class averages
348,170 particles
2x binned
160x160 pixels
Composite mask
2D classification
Select dimers
10 nm
211,846 particles
3D classification
Composite mask
21,633
91,775
82,287
16,151
Re-extract particles
400x400 pixels
3D auto-refine
Composite mask
Composite mask
Composite mask
3D auto-refine
Complex A
3.38 Å
Complex B
3.25 Å
Complex A/B
3.32 Å

Supplementary Fig. 6: Cryo-EM data processing of the 2:2 Wnt5a-WLS dimeric complex

(a) Representative cryo-EM micrograph of the 2:2 Wnt5a-WLS dimeric complex.

(b) Cryo-EM data processing workflow. Representative 2D class averages are shown in the inset, with 2:2 complexes highlighted by red boxes. Distinct composite masks were used in 3D classification and 3D refinement.

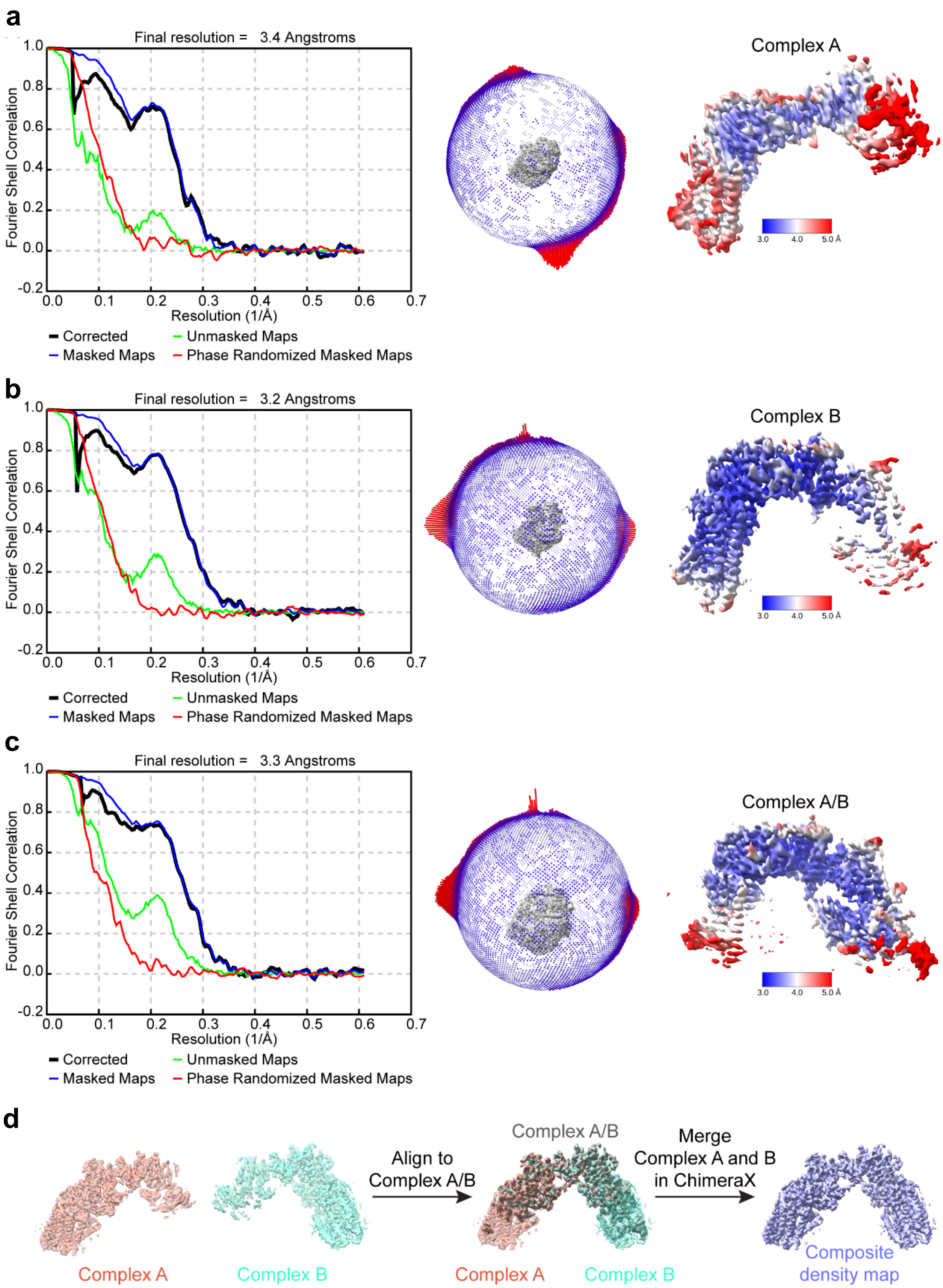

a
Final resolution = 3.4 Angstroms
Fourier Shell Correlation
Resolution (1/Å)
Corrected
Unmasked Maps
Masked Maps
Phase Randomized Masked Maps
Complex A
3.0
4.0
5.0 Å
b
Final resolution = 3.2 Angstroms
Fourier Shell Correlation
Resolution (1/Å)
Corrected
Unmasked Maps
Masked Maps
Phase Randomized Masked Maps
Complex B
3.0
4.0
5.0 Å
c
Final resolution = 3.3 Angstroms
Fourier Shell Correlation
Resolution (1/Å)
Corrected
Unmasked Maps
Masked Maps
Phase Randomized Masked Maps
Complex A/B
3.0
4.0
5.0 Å
d
Complex A
Complex B
Align to Complex A/B
Complex A/B
Complex A
Complex B
Merge Complex A and B in ChimeraX
Composite density map

Supplementary Fig. 7: Resolution estimation of cryo-EM 3D reconstructions of the 2:2 Wnt5a-WLS dimeric complex

(a-c) Gold-standard FSC curve (left), particle orientation plot (middle), and local resolution map (right) of complex A (a), complex B (b), and whole complex (complex A/B) (c) of the 2:2 Wnt5a-WLS dimeric complex.

(d) A composite density map of 2:2 Wnt5a-WLS dimeric complex was produced by aligning the cryo-EM density maps of complex A and B to that of complex A/B and merging two maps together using the "*volume maximum*" command in UCSF ChimeraX.

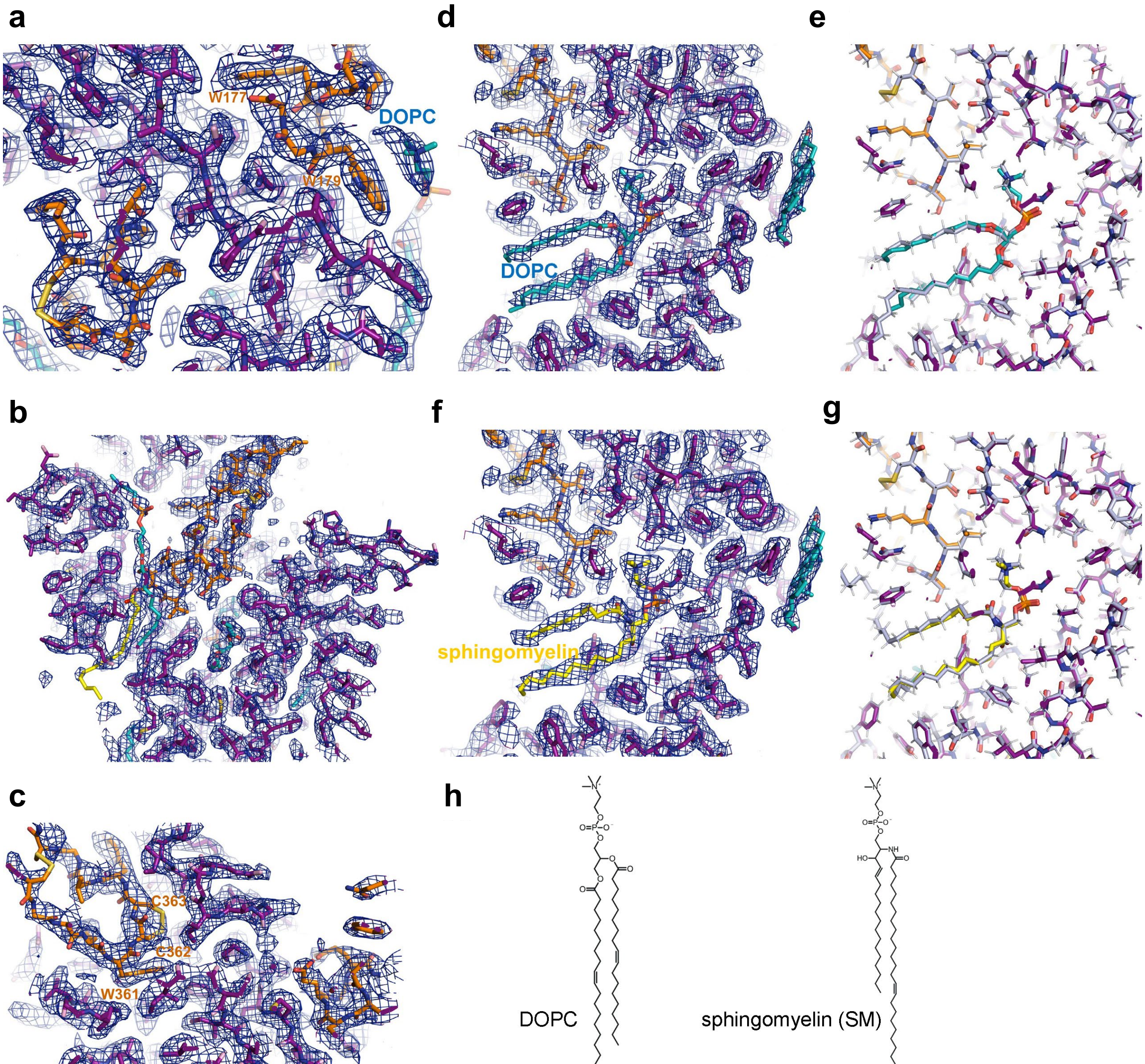

Ligand	Docking score	MM-GBSA ΔG_{bind}
DOPC	-14.714	-125.62
SM	-14.140	-112.10

Supplementary Fig. 8: Density maps (contoured at 10 σ) for representative features in the WLS-Wnt5a 1:1 complex

(a) The interaction site between WLS (deep purple) and hairpin 1 of Wnt5a (orange). DOPC is shown in teal. The same color scheme is used in (b)-(e).

(b) The WLS binding pocket for hairpin 2 of Wnt5a. The PAM moiety is shown in yellow.

(c) The WCC motif of Wnt5a hairpin 3 interacts with the luminal domain of WLS.

(d) Densities surrounding the modeled DOPC (teal) in the central cavity of WLS.

(e) As in (d), but showing comparison of the cryo-EM map–guided model with the computed molecular docking model of DOPC (light blue, with hydrogen atoms displayed).

(f) As in (d), but with sphingomyelin (SM; yellow) built into the density instead of DOPC, demonstrating that SM also fits into the WLS central cavity in the Wnt-bound open conformation.

(g) As in (f), but showing comparison of the cryo-EM map–guided model with the computed molecular docking model of SM (light blue, with hydrogen atoms displayed).

(h) Docking scores and estimated binding free energies for DOPC and SM binding to the WLS central cavity.

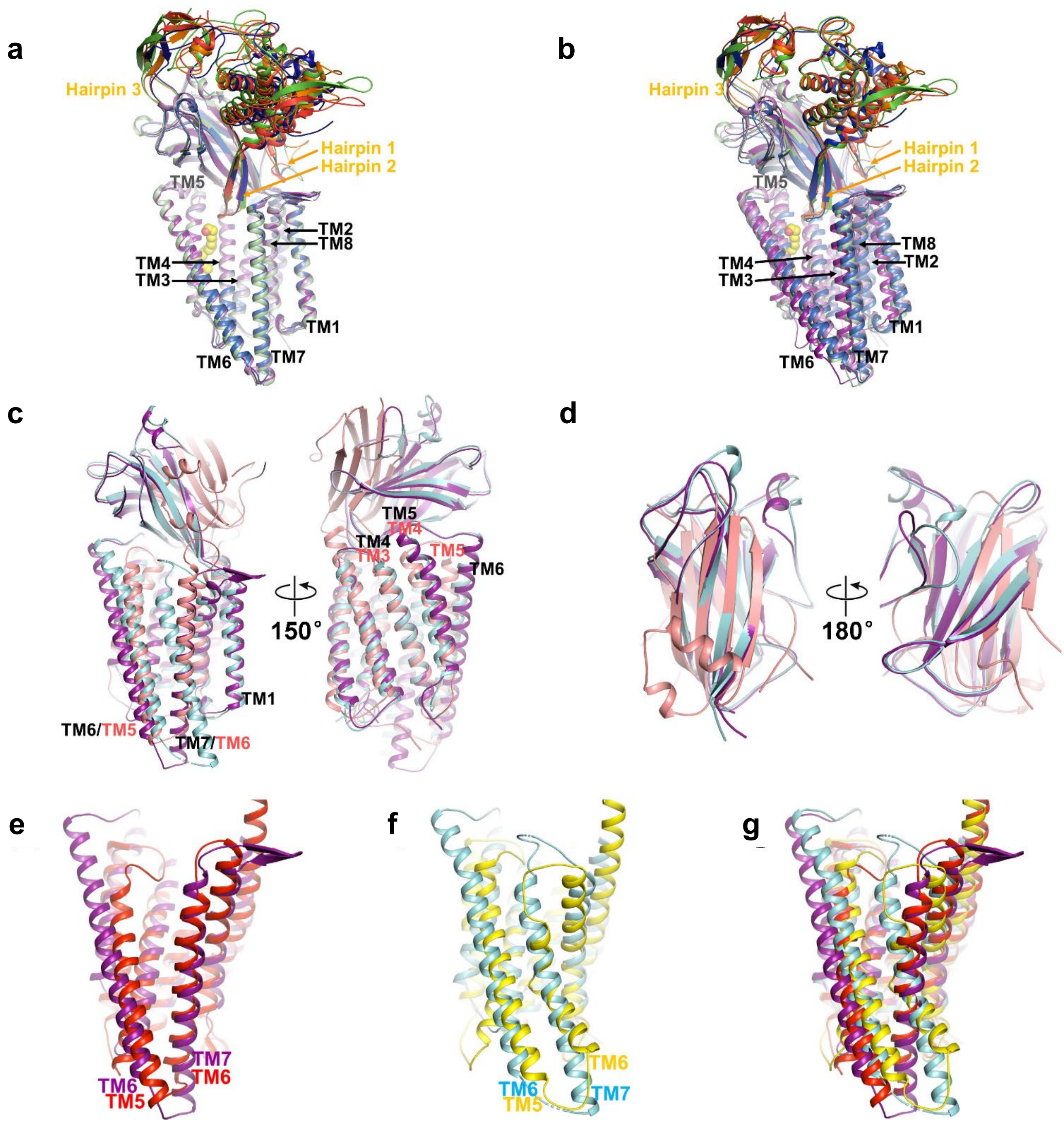
a
Hairpin 3
Hairpin 1
Hairpin 2
TM5
TM2
TM8
TM4
TM3
TM1
TM6
TM7
b
Hairpin 3
Hairpin 1
Hairpin 2
TM5
TM8
TM4
TM2
TM3
TM1
TM6
TM7
c
TM5
TM4
TM4
TM3
TM5
TM6
150°
TM1
TM6/TM5
TM7/TM6
d
180°
e
TM7
TM6
TM6
TM5
f
TM6
TM6
TM5
TM7
g

Supplementary Fig. 9: Comparison between WLS structures in this study and previous structures

(a) Comparison of the WLS-Wnt5a 1:1 complex with WLS-Wnt3a (PDB ID: 7DRT), WLS-Wnt8a (PDB ID: 7KC4) and WLS-Wnt7a (PDB ID: 8TZP) structures, by superimposing on WLS. Wnt5a, Wnt3a, Wnt8a, and Wnt7a are shown in orange, green, blue, and red, respectively. The PAM moiety is displayed as yellow spheres.

(b) As in (a), but superimposing on the core helical bundle of Wnt NTDs.

(c) Structural comparison of WLS in Wnt5a-bound conformation (deep purple), apo WLS (cyan) and TMEM87A (salmon color, PDB ID: 8HSI). The GOLD domain of TMEM87A adopts a different orientation compared to the luminal domain of WLS.

(d) As in (c), but comparing the TMEM87A GOLD domain (salmon) to the WLS luminal domain in Wnt-bound (deep purple) or apo (cyan) conformations.

(e) Overlay of WLS in the Wnt5a-bound conformation (deep purple) with the G protein-free glucagon receptor (red, PDB ID: 5YQZ).

(f) Overlay of apo WLS (cyan) with the G protein-bound, fully activated glucagon receptor (yellow, PDB ID: 6WPW)

(g) Combining (e) and (f).

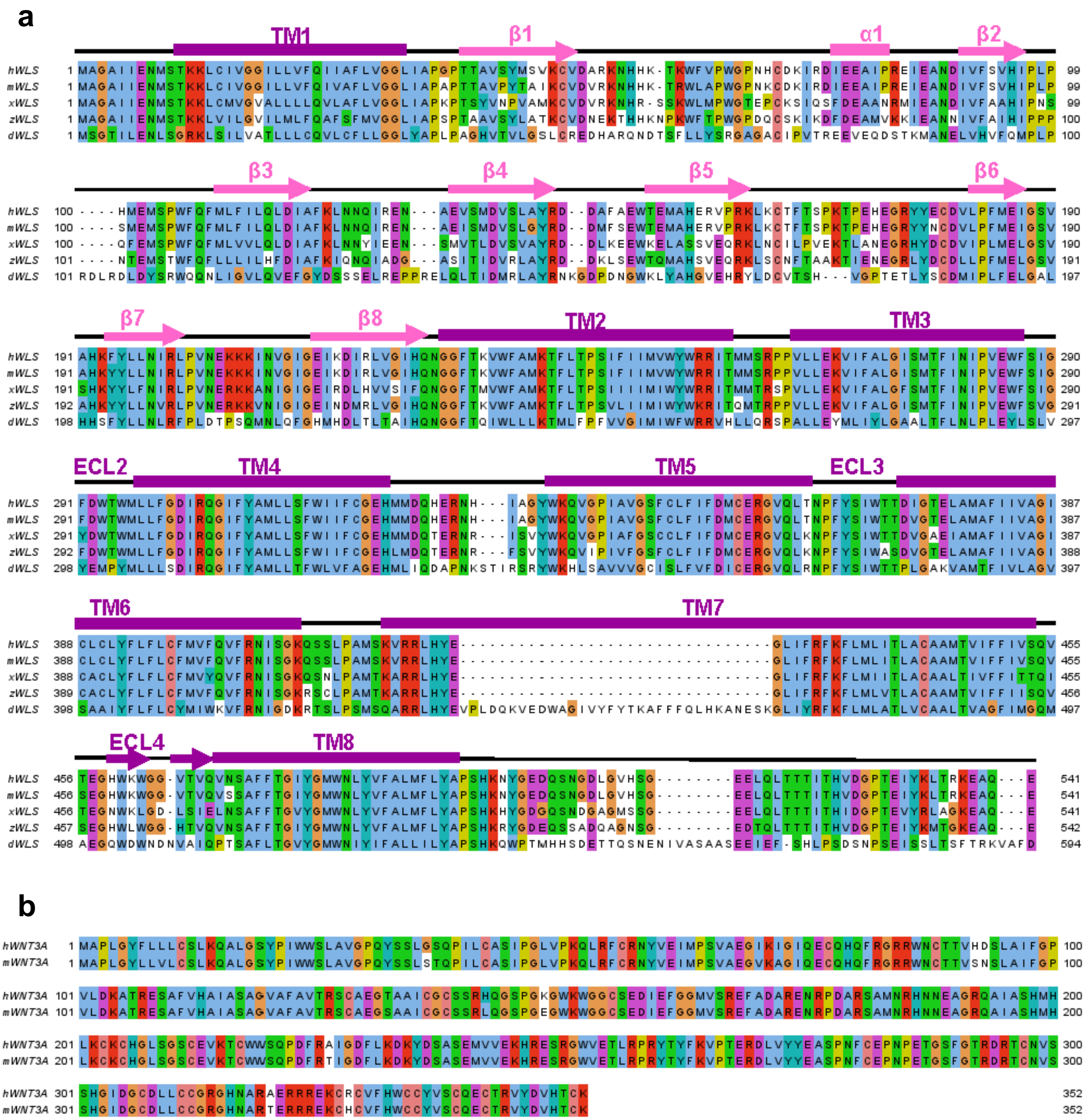

Supplementary Fig. 10: Multiple sequence alignments of WLS and Wnt3a

(a) Multiple sequence alignment and secondary structure of WLS from representative organisms. The transmembrane helices are shown in deep purple, and the luminal domain in pink.

(b) Sequence alignment of human and mouse Wnt3a. Note that their amino acid numbers are identical.

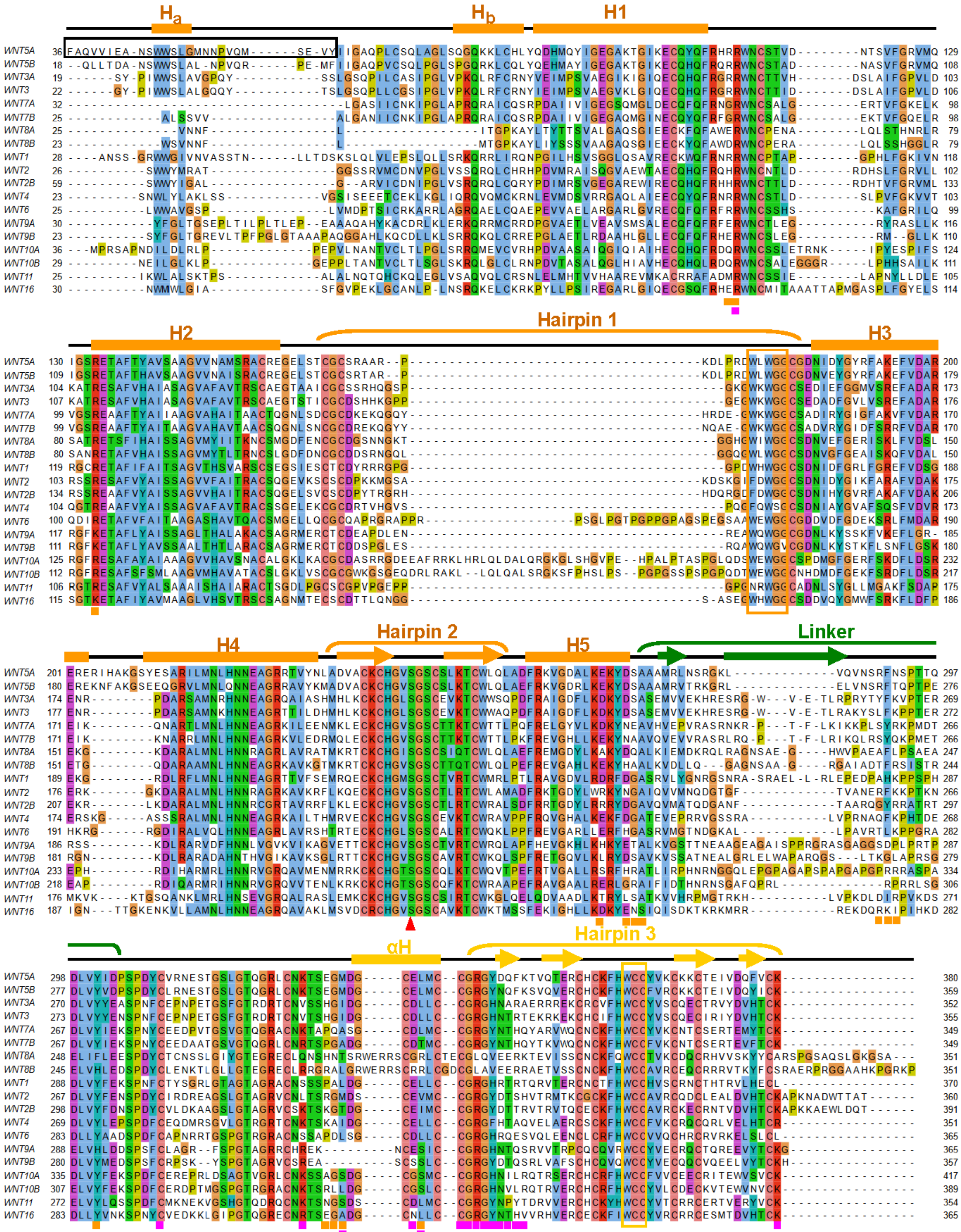

Ha
Hb
H1
H2
Hairpin 1
H3
H4
Hairpin 2
H5
Linker
αH
Hairpin 3
WNT5A
WNT5B
WNT3A
WNT3
WNT7A
WNT7B
WNT8A
WNT8B
WNT1
WNT2
WNT2B
WNT4
WNT6
WNT9A
WNT9B
WNT10A
WNT10B
WNT11
WNT16

Supplementary Fig. 11: Multiple sequence alignment of all 19 human Wnts

The Wnt NTD, linker and CTD are shown in orange, green and yellow, respectively, with secondary structures corresponding to the present WLS-Wnt5a structure. The black box indicates the Wnt5a N-terminal fragment absent in the short isoform. The core helical bundle of Wnt NTD consists of five helices (H1-H5), with additional H_a and H_b present in some Wnts. The orange and yellow boxes indicate the conserved WXWGG motif in hairpin 1 and the WCC motif in hairpin 3, respectively. A red triangle indicates the conserved palmitoleoylated serine. Residues involved in the dimerization interface are indicated by orange squares for Wnt5a Molecule A and by purple squares for Wnt5a Molecule B.

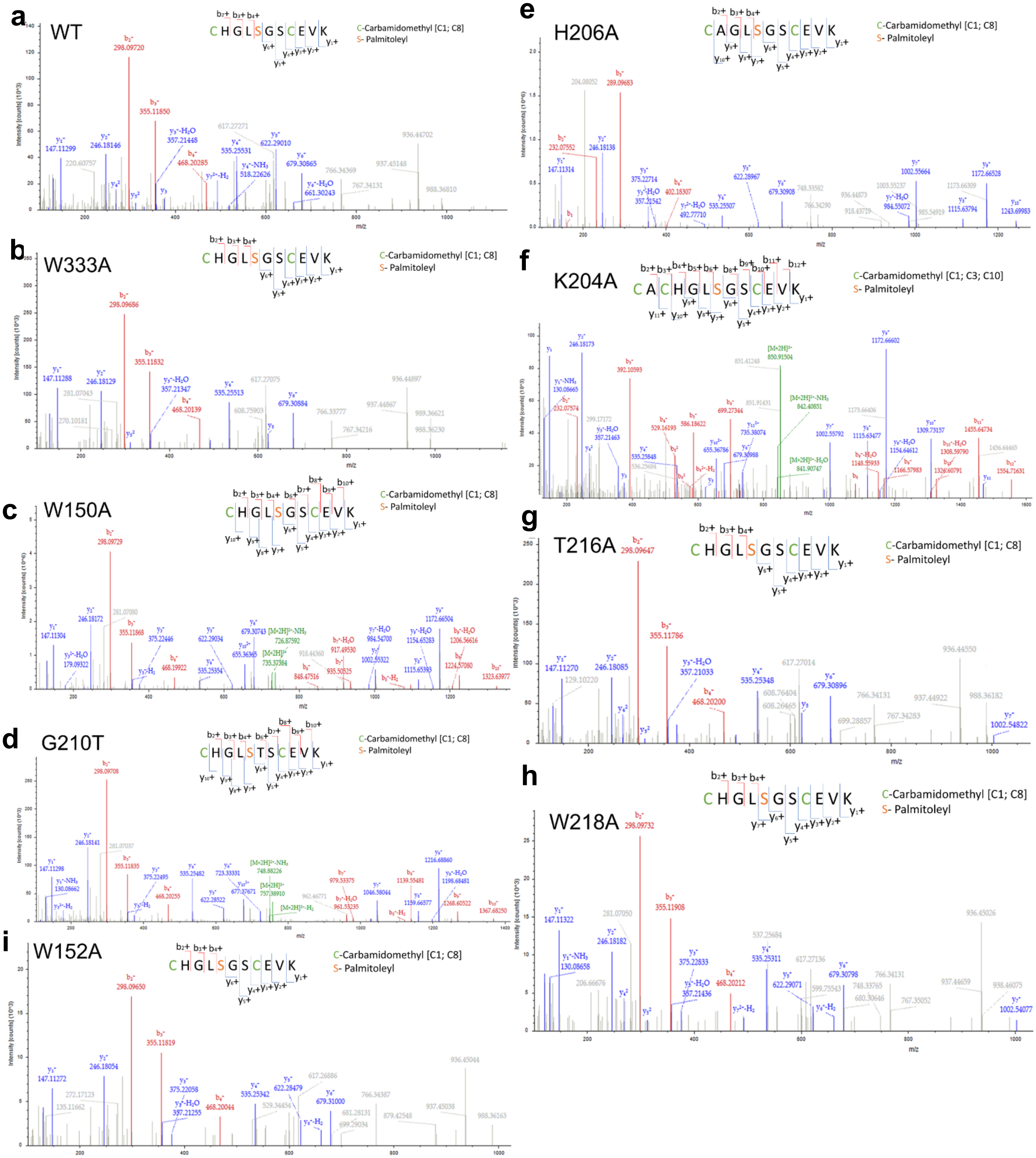
a
WT
CHGLSGSCEVK
C-Carbamidomethyl [C1; C8]
S- Palmitoleyl
b
W333A
CHGLSGSCEVK
C-Carbamidomethyl [C1; C8]
S- Palmitoleyl
c
W150A
CHGLSGSCEVK
C-Carbamidomethyl [C1; C8]
S- Palmitoleyl
d
G210T
CHGLSTSCEVK
C-Carbamidomethyl [C1; C8]
S- Palmitoleyl
e
H206A
CAGLSGSCEVK
C-Carbamidomethyl [C1; C8]
S- Palmitoleyl
f
K204A
CACHGLSGSCEVK
C-Carbamidomethyl [C1; C3; C10]
S- Palmitoleyl
g
T216A
CHGLSGSCEVK
C-Carbamidomethyl [C1; C8]
S- Palmitoleyl
h
W218A
CHGLSGSCEVK
C-Carbamidomethyl [C1; C8]
S- Palmitoleyl
i
W152A
CHGLSGSCEVK
C-Carbamidomethyl [C1; C8]
S- Palmitoleyl

Supplementary Fig. 12: LC-MS/MS analysis of Ser209 palmitoleoylation in mouse Wnt3a mutants used in this study

FLAG-tagged mWnt3a constructs were stably expressed Expi293 cells and were immunopurified from cellular lysates on anti-FLAG beads. Immunoprecipitates were subjected to tryptic digestion and LC-MS/MS analysis, to detect Ser209 palmitoleoylation (see Methods). FLAG-tagged wild type (WT) mWnt3a samples immunopurified from WT and PORCN-KO cells were used as positive and negative controls, respectively.

(a) Palmitoleoylated tryptic peptide in WT mWnt3a.
C(carbamidomethyl)HGLS(palmitoleoyl)GSC(carbamidomethyl)EVK, observed m/z = 490.5878, z = 3, ΔM = -0.14 ppm.

(b) As in (a), but for the mWnt3A-W333A mutant.
C(carbamidomethyl)HGLS(palmitoleoyl)GSC(carbamidomethyl)EVK, observed m/z = 490.5877, z = 3, ΔM = -0.33 ppm.

(c) As in (a), but for the mWnt3A-W150A mutant.
C(carbamidomethyl)HGLS(palmitoleoyl)GSC(carbamidomethyl)EVK, observed m/z = 735.3783, z = 2, ΔM = 0.12 ppm.

(d) As in (a), but for the mWnt3A-G210T mutant.
C(carbamidomethyl)HGLS(palmitoleoyl)TSC(carbamidomethyl)EVK, observed m/z = 757.3910, z = 2, ΔM = -0.41 ppm.

(e) As in (a), but for the mWnt3A- H206A mutant.
C(carbamidomethyl)AGLS(palmitoleoyl)GSC(carbamidomethyl)EVK, observed m/z = 702.3673, z = 2, ΔM = -0.03 ppm.

(f) As in (a), but for the mWnt3A-K204A mutant.
C(carbamidomethyl)AC(carbamidomethyl)HGLS(palmitoleoyl)GSC(carbamidomethyl)EVK, observed m/z = 850.9120, z=2, ΔM = -0.07 ppm.

(g) As in (a), but for the mWnt3A-T216A mutant.
C(carbamidomethyl)HGLS(palmitoleoyl)GSC(carbamidomethyl)EVK, observed m/z = 490.5873, z=3, ΔM = -1.11 ppm.

(h) As in (a), but for the mWnt3A-W218A mutant.
C(carbamidomethyl)HGLS(palmitoleoyl)GSC(carbamidomethyl)EVK, observed m/z = 490.5878, z = 3, ΔM = -0.08 ppm.

(i) As in (a), but for the mWnt3A-W152A mutant.
C(carbamidomethyl)HGLS(palmitoleoyl)GSC(carbamidomethyl)EVK, observed m/z = 490.5875, z = 3, ΔM = -0.84 ppm.

Ser209 palmitoleoylation was not detected in the G207T mutant or in the negative control WT mWnt3a sample purified from PORCN-KO cells.

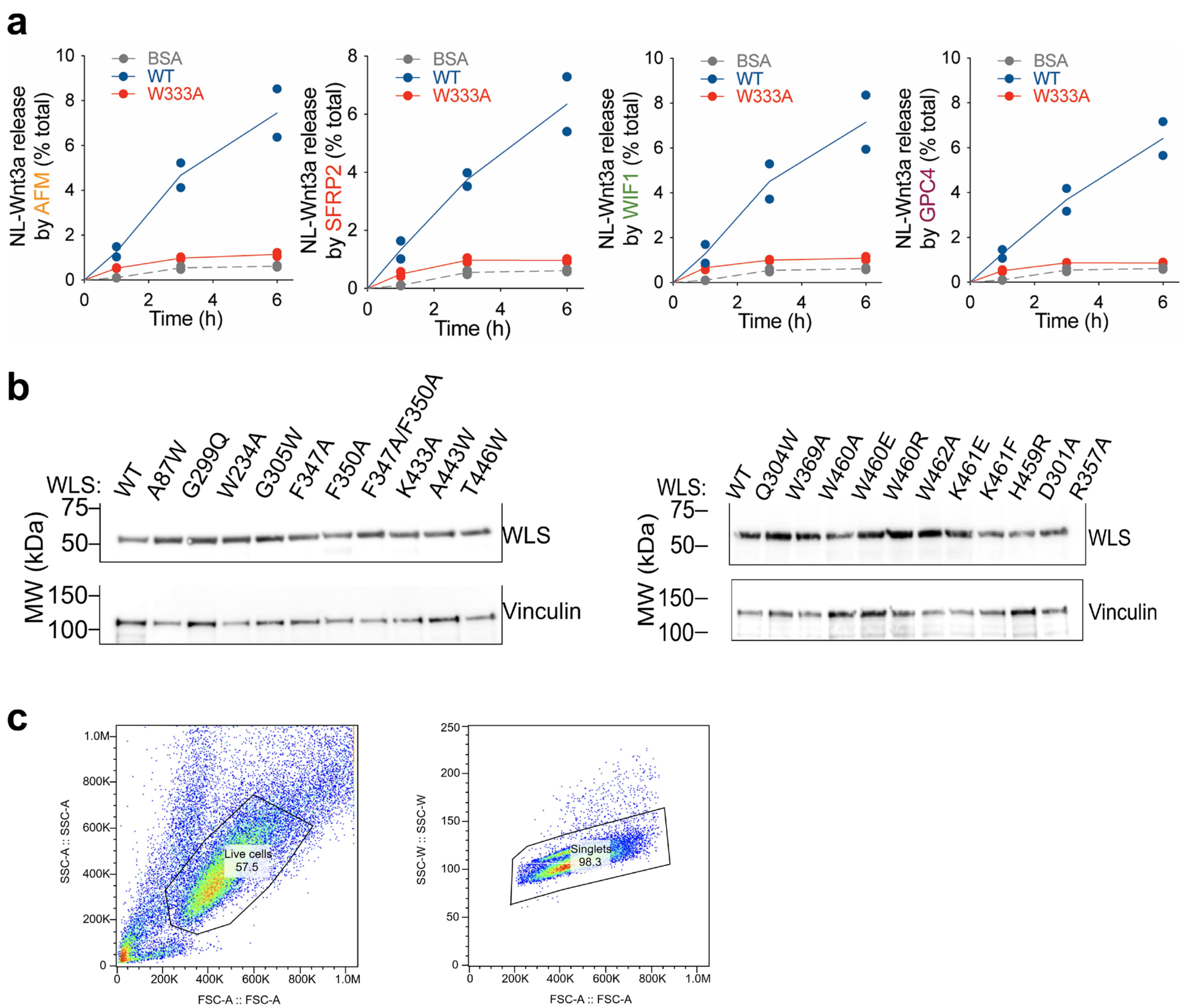

Supplementary Fig. 13: Additional carrier-mediated mWnt3a secretion assays

(a) NanoLuc luciferase (NL)-mWnt3a release assay. HEK293 cells stably expressing NL–mWnt3a WT and the W333A mutant were pre-incubated with cycloheximide (CHX) to inhibit protein synthesis. NL-mWnt3a release was then initiated by adding purified carrier proteins (1 μM) in serum-free medium and measured at various time points by NL luminescence. Data represent the average of two technical replicates (n=2), normalized to total NL–mWnt3a in cell lysates, for a representative experiment. Each experiment has been performed at least twice using distinct biological samples.

(b) WLS mutants exhibited expression levels comparable to the WT, as shown by immunoblotting.

(c) Representative FACS gating strategy used in all studies presented in this work.

a

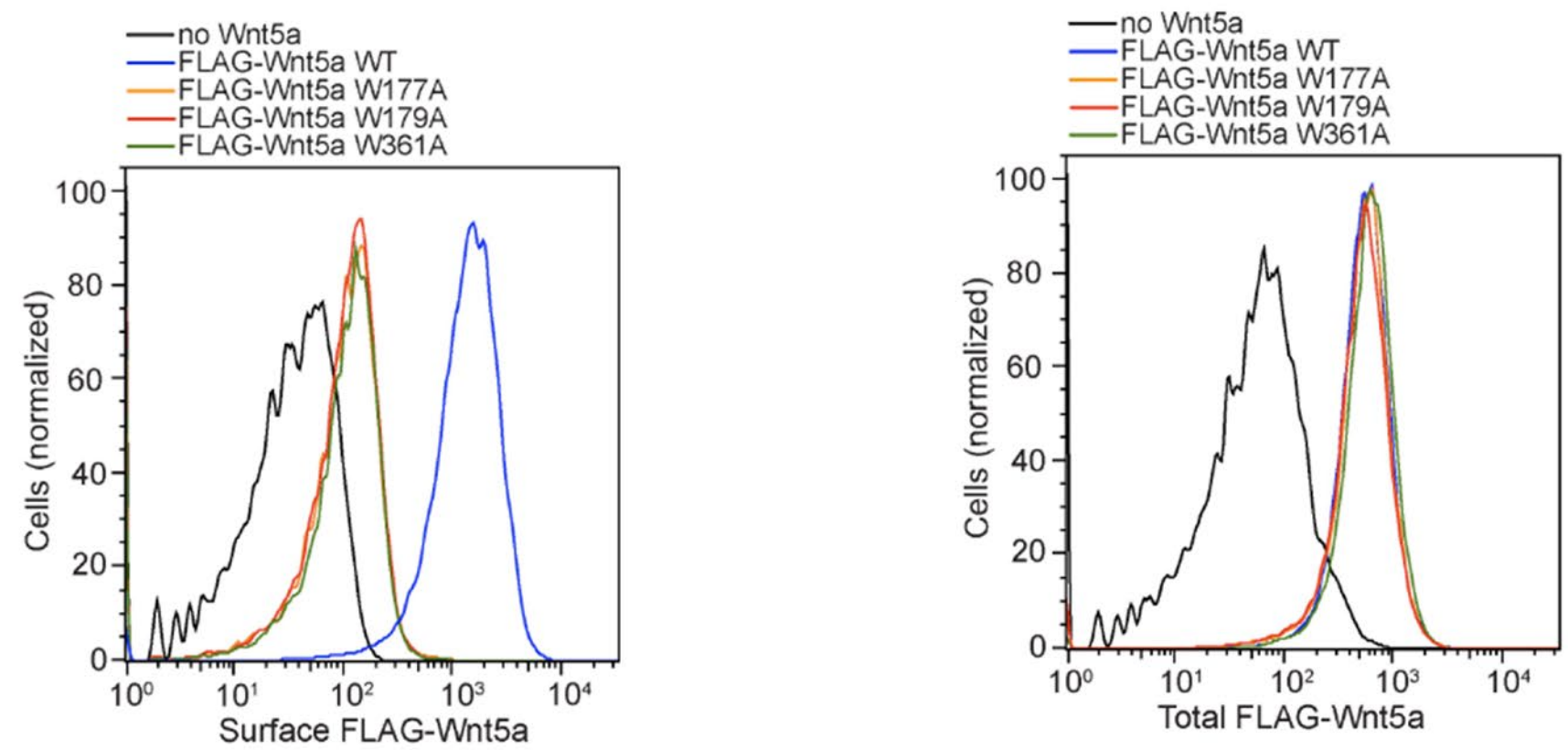

no Wnt5a
FLAG-Wnt5a WT
FLAG-Wnt5a W177A
FLAG-Wnt5a W179A
FLAG-Wnt5a W361A
Cells (normalized)
Surface FLAG-Wnt5a
Total FLAG-Wnt5a

b

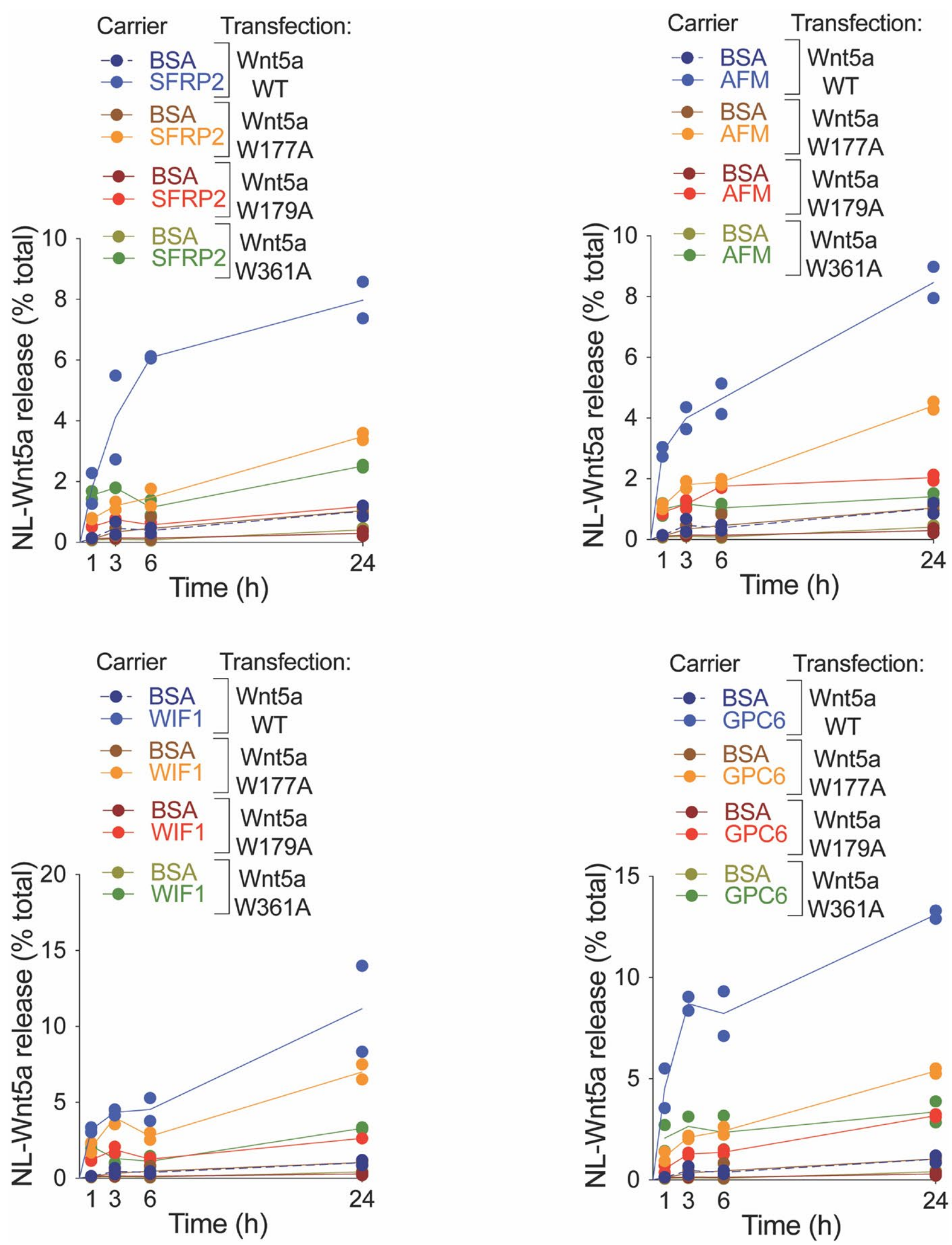

Carrier
Transfection:
BSA
SFRP2
AFM
WIF1
GPC6
Wnt5a WT
Wnt5a W177A
Wnt5a W179A
Wnt5a W361A
NL-Wnt5a release (% total)
Time (h)

Supplementary Fig. 14: Requirement of Wnt5a hairpin 1 and hairpin 3 in WLS-mediated transport and release by carriers

(a) Surface (left, without permeabilization) and total (right, with detergent permeabilization) levels of FLAG-Wnt5a constructs, stained with AlexaFluor647-conjugated anti-FLAG-M1 antibody and analyzed by FACS.

(b) NanoLuc luciferase (NL)–Wnt5a release assay. For HEK293 cells stably expressing NL–Wnt5a constructs, NL–Wnt5a release was initiated by adding the purified carrier proteins (1 μM) in serum-free medium and was measured at various times by NL luminescence. Data represent the average of two technical replicates (n=2), normalized to total NL–Wnt5a in cell lysates, for a representative experiment. Each experiment has been performed at least twice using distinct biological samples.

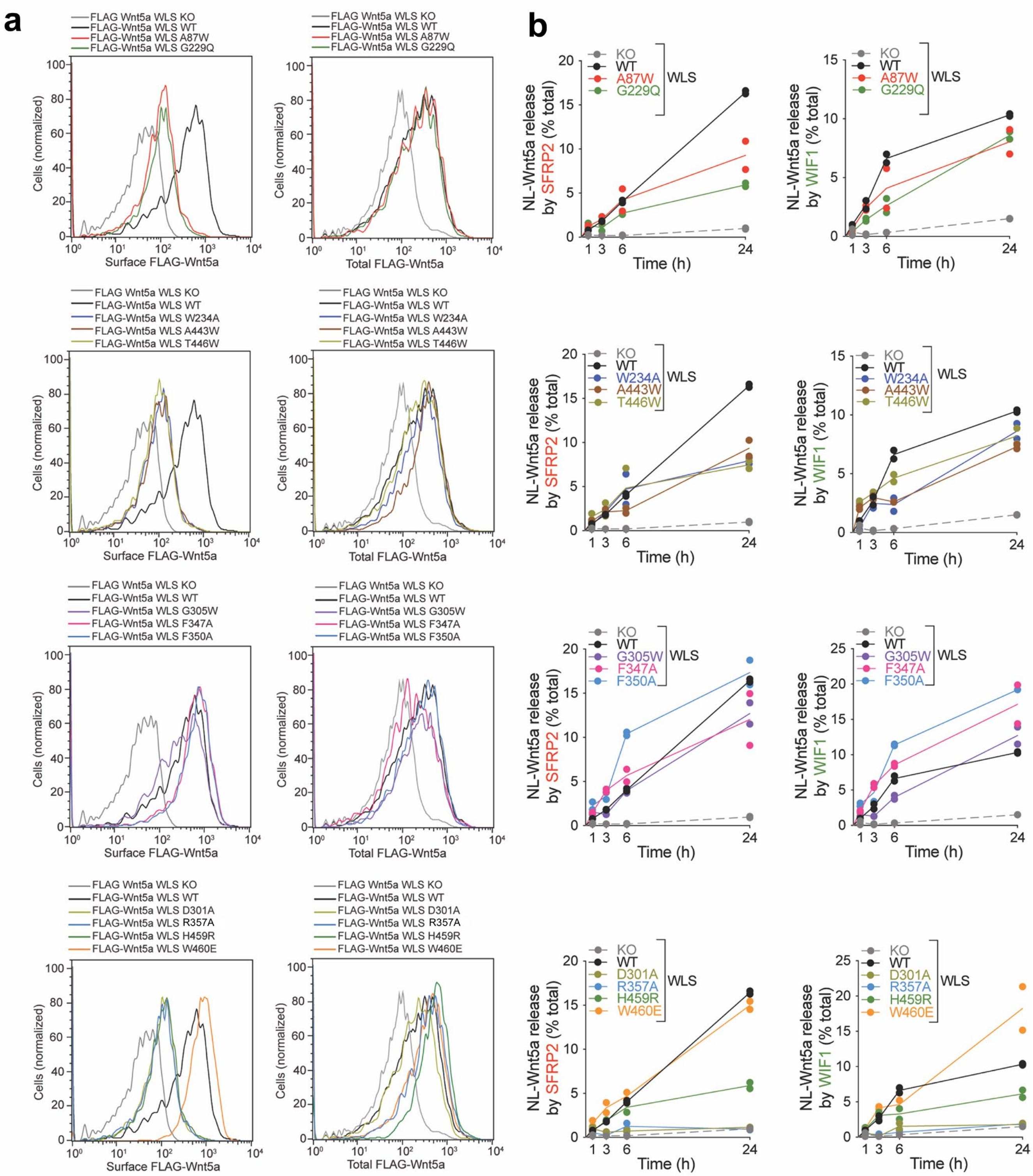
a
FLAG Wnt5a WLS KO
FLAG-Wnt5a WLS WT
FLAG-Wnt5a WLS A87W
FLAG-Wnt5a WLS G229Q
FLAG-Wnt5a WLS W234A
FLAG-Wnt5a WLS A443W
FLAG-Wnt5a WLS T446W
FLAG-Wnt5a WLS G305W
FLAG-Wnt5a WLS F347A
FLAG-Wnt5a WLS F350A
FLAG-Wnt5a WLS D301A
FLAG-Wnt5a WLS R357A
FLAG-Wnt5a WLS H459R
FLAG-Wnt5a WLS W460E
Cells (normalized)
Surface FLAG-Wnt5a
Total FLAG-Wnt5a
b
KO
WT
A87W
G229Q
W234A
A443W
T446W
G305W
F347A
F350A
D301A
R357A
H459R
W460E
WLS
NL-Wnt5a release by SFRP2 (% total)
NL-Wnt5a release by WIF1 (% total)
Time (h)

Supplementary Fig. 15: Key WLS residues involved in Wnt5a transport and release by carriers
(a) Surface (left) and total (right) levels of FLAG-Wnt5a in WLS-KO cells stably expressing the indicated WLS constructs. Cells were stained with anti-FLAG-M1-AlexaFluor647 and were analyzed by FACS.
(b) For WLS-KO cells stably expressing NL–Wnt5a together with the indicated WLS constructs, NL–Wnt5a release was initiated by adding purified SFRP2 or WIF1 (1 μM) in serum-free medium, and was measured at various times by NL luminescence. Data represent the average of two technical replicates (n=2), normalized to total NL–Wnt5a in cell lysates, for a representative experiment. Each experiment has been performed at least twice using distinct biological samples.

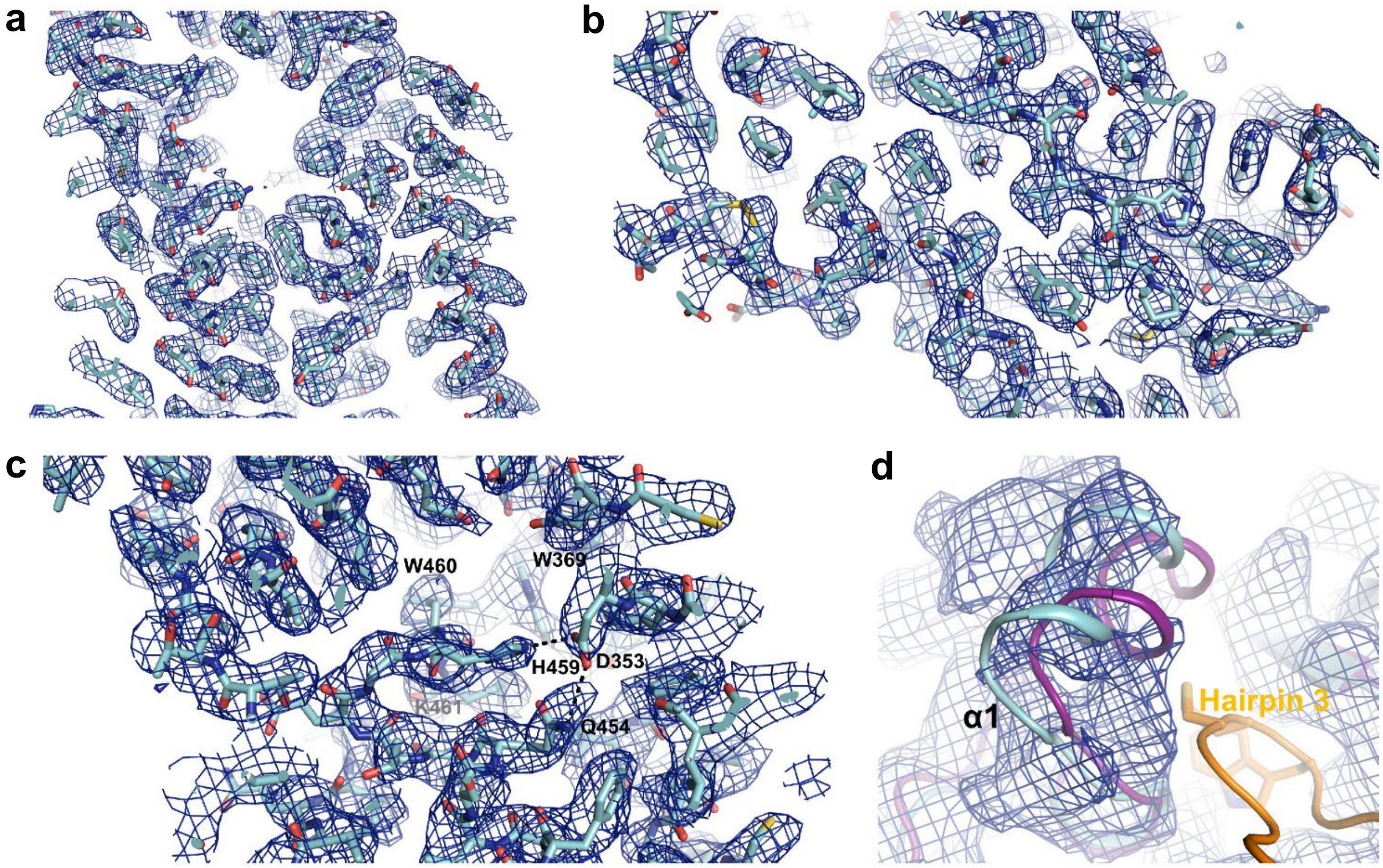

Supplementary Fig. 16: Density maps for representative features in apo WLS structure

(a) Density map contoured at 10 σ, showing the transmembrane region of WLS.

(b) As in (a), but showing the luminal region.

(c) Density map contoured at 8 σ, highlighting ECL4 that occupies the cavity vacated following Wnt release. Key residues are labeled, with dashed lines indicating hydrogen bonds.

(d) Unsharpened density map contoured at 5σ, surrounding the α1 helix of the WLS luminal domain, which forms the binding site for Wnt5a hairpin 3 (orange). Apo WLS and Wnt5a-bound conformations are shown in cyan and deep purple, respectively.

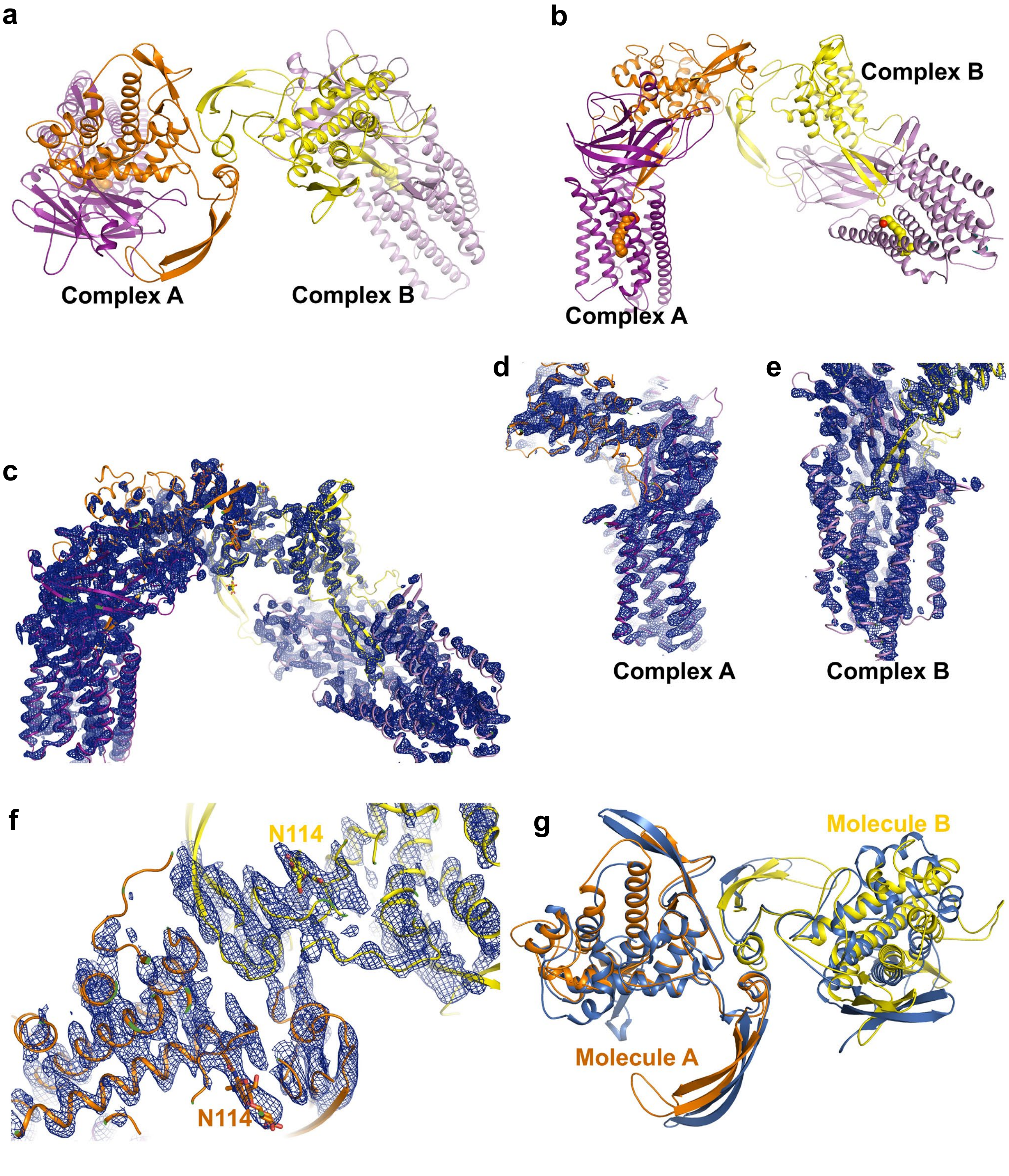
a
Complex A
Complex B
b
Complex B
Complex A
c
d
e
Complex A
Complex B
f
N114
N114
g
Molecule B
Molecule A

Supplementary Fig. 17: Cryo-EM structure of the WLS-Wnt5a 2:2 complex

(a) Same view as in Fig. 6d, with WLS included.

(b) Same view as in Fig. 6e, with WLS included.

(c) Composite density map contoured at 9 σ, covering the entire 2:2 complex.

(d) Composite density map contoured at 9 σ, covering Complex A.

(e) Composite density map contoured at 9 σ, covering Complex B.

(f) Composite density map contoured at 9 σ, highlighting the dimerization interface. The glycosylated N114 residues are labeled.

(g) Comparison between the Wnt5a dimer in the 2:2 Wnt5a–WLS complex and the Wnt3a dimer (blue) in the Wnt3a–FZD8–LRP6 extracellular complex (PDB ID: 21KT). The Wnt3a dimer is aligned onto molecule A of the Wnt5a dimer.

Supplementary Table 1: Cryo-EM data collection, refinement and validation statistics

	Wnt5a-WLS 1:1 complex (EMDB: 73803) (PDB: 9Z4H)	apo WLS in nanodiscs (EMDB: 73810) (PDB: 9Z4O)	Wnt5a-WLS 2:2 complex (EMDB: 73838) (PDB: 9Z67)
**Data collection and processing**			
Magnification	105,000×	105,000×	105,000×
Voltage (kV)	300	300	300
Electron exposure (e–/Å^2)	50	50	50
Defocus range (μm)	-0.8 to -2.4	–0.8 to –2.4	–0.8 to –2.4
Pixel size (Å)	0.83	0.82	0.82
Symmetry imposed	C1	C1	C1
Initial particle images (no.)	5,253,505	15,802,189	11,618,191
Final particle images (no.)	104,884	86,035	174,062
Map resolution (Å)	2.64	2.63	3.32
FSC threshold	0.143	0.143	0.143
Map resolution range (Å)			
**Refinement**			
Model composition			
Non-hydrogen atoms	6,913	3,920	13,366
Protein residues	815	481	1,630
Ligands	PCW: 5, LPE: 1<br>PAM: 1, CLR: 1<br>NAG: 4		PCW: 2, PAM: 2<br>CLR: 2, NAG: 8
Water	0	0	0
R.m.s. deviations			
Bond lengths (Å)	0.003	0.003	0.003
Bond angles (°)	0.617	0.624	0.617
Validation			
MolProbity score	1.67	1.69	1.87
Clashscore	7.93	7.51	13.23
Poor rotamers (%)	0.99	1.65	0.99
Ramachandran plot			
Favored (%)	96.42	97.46	96.42
Allowed (%)	3.58	2.54	3.58
Disallowed (%)	0	0	0
