# Supplementary material for: Conformational cycling of the Wntless transporter drives trafficking and secretion of Wnt morphogens
Source: Nat Commun. 2026 Jun 19;17:7737. doi: 10.1038/s41467-026-74521-y (PMC13434646; doi:10.1038/s41467-026-74521-y)
Supplement: Supplementary file 2 [file 41467_2026_74521_MOESM2_ESM.pdf]

Title: Supplementary Video 1
Description: Conformational changes in WLS upon Wnt5a release

Title: Supplementary Video 2
Description: Conformational changes in the glucagon receptor upon activation
