# Supplementary material for: Conformational cycling of the Wntless transporter drives trafficking and secretion of Wnt morphogens
Source: Nat Commun. 2026 Jun 19;17:7737. doi: 10.1038/s41467-026-74521-y (PMC13434646; doi:10.1038/s41467-026-74521-y)
Supplement: Supplementary file 3 [file 41467_2026_74521_MOESM5_ESM.pdf]

nature portfolio

Corresponding author(s): Yunhui Ge, Taciani de Almeida Magalhaes, Jiansen Jiang, Adrian Salic, Pengxiang Huang

Last updated by author(s): May 27, 2026

Reporting Summary

Nature Portfolio wishes to improve the reproducibility of the work that we publish. This form provides structure for consistency and transparency in reporting. For further information on Nature Portfolio policies, see our Editorial Policies and the Editorial Policy Checklist.

Statistics

For all statistical analyses, confirm that the following items are present in the figure legend, table legend, main text, or Methods section.

n/a	Confirmed	
☐	☒	The exact sample size (*n*) for each experimental group/condition, given as a discrete number and unit of measurement
☐	☒	A statement on whether measurements were taken from distinct samples or whether the same sample was measured repeatedly
☒	☐	The statistical test(s) used AND whether they are one- or two-sided<br>*Only common tests should be described solely by name; describe more complex techniques in the Methods section.*
☒	☐	A description of all covariates tested
☒	☐	A description of any assumptions or corrections, such as tests of normality and adjustment for multiple comparisons
☒	☐	A full description of the statistical parameters including central tendency (e.g. means) or other basic estimates (e.g. regression coefficient) AND variation (e.g. standard deviation) or associated estimates of uncertainty (e.g. confidence intervals)
☒	☐	For null hypothesis testing, the test statistic (e.g. *F*, *t*, *r*) with confidence intervals, effect sizes, degrees of freedom and *P* value noted<br>*Give P values as exact values whenever suitable.*
☒	☐	For Bayesian analysis, information on the choice of priors and Markov chain Monte Carlo settings
☒	☐	For hierarchical and complex designs, identification of the appropriate level for tests and full reporting of outcomes
☒	☐	Estimates of effect sizes (e.g. Cohen's *d*, Pearson's *r*), indicating how they were calculated

Our web collection on statistics for biologists contains articles on many of the points above.

Software and code

Policy information about availability of computer code

Data collection	Leginon 3.5
Data analysis	RELION 4.0, cryoSPARC v3.2, MotionCor2 1.4.2, CTFFIND 4.1.14, and Topaz v0.2.4

For manuscripts utilizing custom algorithms or software that are central to the research but not yet described in published literature, software must be made available to editors and reviewers. We strongly encourage code deposition in a community repository (e.g. GitHub). See the Nature Portfolio guidelines for submitting code & software for further information.

Data

Policy information about availability of data

All manuscripts must include a data availability statement. This statement should provide the following information, where applicable:

- Accession codes, unique identifiers, or web links for publicly available datasets
- A description of any restrictions on data availability
- For clinical datasets or third party data, please ensure that the statement adheres to our policy

The cryo-EM density maps of apo WLS, the Wnt5a-WLS 1:1 complex, and the composite map of the Wnt5a-WLS 2:2 complex have been deposited in the Electron Microscopy Data Bank (EMDB) under accession codes EMD-73810, EMD-73803 and EMD-73838, respectively. For the Wnt5a-WLS 2:2 complex, additional focused maps for Complex A, Complex B, and the consensus map have been deposited under accession codes EMD-73835, EMD-73836 and EMD-73837, respectively. The corresponding atomic coordinates of these structures have been deposited in the Protein Data Bank (PDB) under accession codes 9Z4O for apo WLS, 9Z4H for the Wnt5a-WLS 1:1 complex, and 9Z67 for the Wnt5a-WLS 2:2 complex, respectively. Mass spectrometry data generated in this study have been deposited in MassIVE

under accession number MSV000101445. All other data are available in the main text or the supplementary materials. Source data are provided with this paper.

Research involving human participants, their data, or biological material

Policy information about studies with human participants or human data. See also policy information about sex, gender (identity/presentation), and sexual orientation and race, ethnicity and racism.

Reporting on sex and gender	This study does not involve human participants, their data, or biological material.
Reporting on race, ethnicity, or other socially relevant groupings	This study does not involve human participants, their data, or biological material.
Population characteristics	This study does not involve human participants, their data, or biological material.
Recruitment	This study does not involve human participants, their data, or biological material.
Ethics oversight	This study does not involve human participants, their data, or biological material.

Note that full information on the approval of the study protocol must also be provided in the manuscript.

Field-specific reporting

Please select the one below that is the best fit for your research. If you are not sure, read the appropriate sections before making your selection.

[x] Life sciences [ ] Behavioural & social sciences [ ] Ecological, evolutionary & environmental sciences

For a reference copy of the document with all sections, see nature.com/documents/nr-reporting-summary-flat.pdf

Life sciences study design

All studies must disclose on these points even when the disclosure is negative.

Sample size	Sample sizes for cryo-EM (numbers of particles or micrographs) were chosen to assure reaching the highest possible resolution with a consideration of the performance of the electron microscope and the detector.
Data exclusions	Cryo-EM micrographs with a resolution worse than 4 angstroms as reported by CTFFIND4 were excluded. Cryo-EM images of protein particles that belong to poor 2D classes or 3D classes were excluded during data processing using RELION4.
Replication	Protein purification and cryo-EM sample preparation were repeated >=2 times for each of the reported cryo-EM structures. For functional assays, as stated in the respective figure legends, data represent the average from two technical replicates (n = 2), normalized to the corresponding control, from a representative experiment. Each experiment was performed at least twice using distinct biological samples.
Randomization	Randomization was done automatically by the cryo-EM data processing software for 3D refinement to calculate gold-standard Fourier shell correlation.
Blinding	The initial model of the cryo-EM structure was generated directly from the cryo-EM images without prior knowledge of the structure or any human supervision.

Reporting for specific materials, systems and methods

We require information from authors about some types of materials, experimental systems and methods used in many studies. Here, indicate whether each material, system or method listed is relevant to your study. If you are not sure if a list item applies to your research, read the appropriate section before selecting a response.

Materials & experimental systems

n/a	Involved in the study	
[ ]	[x]	Antibodies
[ ]	[x]	Eukaryotic cell lines
[x]	[ ]	Palaeontology and archaeology
[x]	[ ]	Animals and other organisms
[x]	[ ]	Clinical data
[x]	[ ]	Dual use research of concern
[x]	[ ]	Plants

Methods

n/a	Involved in the study	
[x]	[ ]	ChIP-seq
[ ]	[x]	Flow cytometry
[x]	[ ]	MRI-based neuroimaging

Antibodies

Antibodies used	The following antibodies were obtained commercially: rabbit monoclonals against Wnt3a and Vinculin (Cell Signaling Technology); mouse monoclonal anti-WLS (BioLegend); rabbit polyclonal anti-PORCN (Novus Biologicals); and mouse monoclonal anti-!-tubulin (Santa Cruz Biotechnology). Mouse anti-FLAG-M1 and anti-HPC monoclonal antibodies were kindly provided by Andrew C. Kruse (Harvard Medical School). For immunoblotting, primary antibodies were diluted to 1–2 µg/mL in TBST [10 mM Tris-HCl (pH 7.6), 150 mM NaCl, 0.2% Triton X-100 (v/v)] with 5% (w/v) non-fat dry milk. For immunoblots using anti-FLAG-M1 and anti-HPC antibodies, all buffers were supplemented 2 mM CaCl2. HRP-conjugated secondary antibodies were used at 0.2 µg/mL and were sheep antimouse IgG–HRP (Jackson ImmunoResearch) and donkey anti-rabbit IgG–HRP (GE Healthcare). For flow cytometry, AlexaFluor647-conjugated anti-FLAG-M1 antibody was used at 1 µg/mL in PBS supplemented with 2% FBS, 2 mM CaCl2 and 10 mM HEPES.
Validation	These are commercially available antibodies and widely accepted by the scientific community.

Eukaryotic cell lines

Policy information about cell lines and Sex and Gender in Research

Cell line source(s)	Human embryonic kidney (HEK293) cells were purchased from ATCC, while FreeStyle™ 293F and Expi293 cells were purchased from Thermo Fisher Scientific. All mammalian cell lines were cultured under standard conditions (37°C, humidified atmosphere with 5% CO2) in DMEM (Corning) supplemented with 10% fetal bovine serum (VWR) and penicillin-streptomycin mix (Corning). For protein expression, FreeStyle™ 293F and Expi293 cells were grown in suspension in FreeStyle medium (Thermo Fisher Scientific) at 37°C and 8% CO2, with agitation at 125 rpm in vented-cap plastic flasks (Corning). Spodoptera frugiperda Sf9 cells (Expression Systems) used to generate BacMam viruses were grown in ESF 921 insect cell medium (Expression Systems), in a shaking incubator at 27°C.
Authentication	These commercially available cell lines were authenticated by their respective vendors.
Mycoplasma contamination	No mycoplasma contamination was detected.
Commonly misidentified lines (See ICLAC register)	To our best knowledge, there are no commonly misidentified lines.

Plants

Seed stocks	This study does not involve plants.
Novel plant genotypes	This study does not involve plants.
Authentication	This study does not involve plants.

Flow Cytometry

Plots

Confirm that:

- [x] The axis labels state the marker and fluorochrome used (e.g. CD4-FITC).
- [x] The axis scales are clearly visible. Include numbers along axes only for bottom left plot of group (a 'group' is an analysis of identical markers).
- [ ] All plots are contour plots with outliers or pseudocolor plots.
- [x] A numerical value for number of cells or percentage (with statistics) is provided.

Methodology

Sample preparation	HEK293T cells expressing FLAG-mWnt3a or FLAG-Wnt5a constructs were dissociated using enzyme-free cell dissociation solution (Sigma Aldrich), washed and incubated for 20 min on ice with AlexaFluor647-conjugated anti-FLAG antibody in staining buffer (PBS with 2% FBS, 2mM CaCl2 and 10mM HEPES), with or without Triton X-100 permeabilization, for detecting total and cell surface Wnt levels, respectively. After two washes with staining buffer, cells were analyzed using an Attune flow cytometer (Thermo-Fisher).
Instrument	Thermo Scientific™ Invitrogen™ Attune™ NxT Acoustic Focusing Cytometer, Model AFC2
Software	FlowJo software version 10

Cell population abundance	No cell sorting was performed in this study.
Gating strategy	Live cells were gated using FSC-A/SSC-A, and singlets were gated using FSC-A/SSC-W.

☒ Tick this box to confirm that a figure exemplifying the gating strategy is provided in the Supplementary Information.
