# Supplementary material for: Conformational cycling of the Wntless transporter drives trafficking and secretion of Wnt morphogens
Source: Nat Commun. 2026 Jun 19;17:7737. doi: 10.1038/s41467-026-74521-y (PMC13434646; doi:10.1038/s41467-026-74521-y)
Supplement: Supplementary file 4 [file 41467_2026_74521_MOESM6_ESM.pdf]

nature portfolio

# Peer Review File

# Conformational cycling of the Wntless transporter drives trafficking and secretion of Wnt morphogens

Corresponding Author: Dr Pengxiang Huang

This manuscript has been previously reviewed at another journal. This document only contains information relating to versions considered at Nature Communications.

**This file contains all reviewer reports in order by version, followed by all author rebuttals in order by version.**

Version 0:

Reviewer comments:

Reviewer #1

(Remarks to the Author)
In this study, Ge et al. present cryo-EM structures of the Wnt5a–WLS complex and WLS alone, perform structure-guided mutagenesis and functional analyses, and use structural modeling to propose mechanisms by which WLS transports and presents WNTs to extracellular carriers. The authors further report that WNT5A–WLS can form a 2:2 quaternary complex and speculate that WNT5A may bind Frizzled receptors as a dimer, potentially distinguishing canonical and noncanonical WNT signaling.
However, the structural insights provided by the WNT5A–WLS complex are limited, as the structure is nearly identical to previously reported WLS complexes with Wnt8, Wnt3a, and Wnt7. Moreover, the proposed mechanism that noncanonical WNT5A signals through dimerization is based on limited and preliminary evidence and is not convincingly supported by the current data.

Specific Comments
1. Interpretation of the Wnt3a W150A/W152A mutant secretion defect
In Fig. 2, the authors show that the mWnt3a W150A/W152A mutant is no longer secreted extracellularly in a WLS-dependent manner. However, this phenotype may arise from at least two distinct mechanisms. One possibility is impaired binding to WLS, preventing transport to the plasma membrane. Alternatively, the mutation may disrupt interactions with extracellular Wnt carrier proteins (such as sFRPs or WIF), resulting in reduced retention at the cell surface or in conditioned medium. Notably, the study cited by the authors (PMID: 34315898) employed autocrine signaling assays and demonstrated that Wnt3a W150A/W152A can still activate signaling in producing cells, suggesting that the mutant can still be transported by WLS. These findings imply that the W150A/W152A mutation may primarily affect interactions with extracellular carriers rather than WLS-dependent transport.
The statement "This contrasts with previous reports suggesting that W150A has minimal impact on Wnt secretion and signaling; we speculate this discrepancy might be due to past studies not using physiological carriers to assay Wnt secretion" is therefore not logically consistent with the cited evidence and requires clarification or revision.

2. Potential role of Afamin in Wnt3a mutant secretion
In the referenced study (PMID: 34315898), Wnt3a-conditioned medium was collected without adding exogenous of Wnt carrier proteins; under these conditions, secreted Wnt3a is expected to associate with serum-derived Afamin. The authors should therefore consider examining whether Afamin can facilitate secretion or extracellular stabilization of the Wnt3a W150A/W152A mutant, particularly given that sFRPs and WIF1 may inhibit Wnt–FZD interactions.

3. Quantification of membrane-associated Wnt3a W333A
In Fig. 4B, the authors state that the Wnt3a W333A mutant exhibits reduced membrane-associated protein levels (line 235). However, based on the data presented, the membrane-associated signal of the W333A mutant appears higher than that of wild-type Wnt3a. This apparent discrepancy between the quantitative analysis and the displayed data should be clarified.

4. Generalization from Wnt3a to Wnt5a
Although Wnt3a and Wnt5a share sequence similarity, they exhibit distinct signaling properties and biological functions.

When investigating the role of the hairpin structure in Wnt–WLS interactions and Wnt secretion, it would substantially strengthen the conclusions if comparable analyses were performed using Wnt5a.

5. Interpretation of the Ha helix and RECK interaction
Line 327 states: "Canonical Wnt3a also possesses this Ha helix, which would sterically clash with the Wnt7-specific coreceptor RECK, explaining why Wnt3a does not require RECK for signaling." This interpretation appears imprecise. If correct, it would imply that removal of the Ha helix from Wnt3a could enable RECK binding. The authors should clarify this point or revise the statement.

6. Role of the Wnt N-terminal region in LRP5/6 binding
Line 331 states that "the Wnt N-terminal region is likely dispensable for LRP5/6 binding." However, previous work (PMID: 36994853) demonstrated that Tiki-cleaved Wnt3a (without N-terminus), secreted in complex with FZD-CRD, no longer interacts with LRP6. This discrepancy should be addressed and discussed.

7. Use of N-terminally Flag-tagged Wnt5a
In the first part of the manuscript, the authors used C-terminally FLAG-tagged WNT5A to co-express with WLS and determine the complex structure, which predominantly revealed a 1:1 WNT5A–WLS stoichiometry. However, when proposing the 2:2 quaternary complex, the authors used N-terminally FLAG-tagged WNT5A. The rationale for using different FLAG tag positions should be clearly explained. In addition, the possibility that a FLAG tag interferes with WNT5A behavior or assembly in structural analyses should be carefully considered.

Reviewer #2

(Remarks to the Author)
I co-reviewed this manuscript with one of the reviewers who provided the listed reports. This is part of the Nature Communications initiative to facilitate training in peer review and to provide appropriate recognition for Early Career Researchers who co-review manuscripts.

Reviewer #3

(Remarks to the Author)
In the manuscript entitled "Conformational cycling of the Wntless transporter drives trafficking and secretion of Wnt morphogens", Ge et al. describe cryo-EM structures of the WLS–Wnt5a complex, an improved-resolution apo WLS structure, and a previously unreported 2:2 WLS–Wnt5a assembly.
Although the overall architecture of the WLS–Wnt5a complex closely resembles previously reported WLS–Wnt structures, the study introduces two potentially significant advances. Wnt5a is the first non-canonical Wnt characterized in complex with WLS, and the dimeric interface observed in the 2:2 complex has not been described previously. However, these novel structural observations are not followed by focused functional interrogation. Instead, the majority of mutational and functional analyses are performed on canonical Wnt3a, for which higher-resolution structural information already exists. As a result, the mechanistic insight gained directly from the newly determined Wnt5a structures remains limited.

Nonetheless, the study highlights discrepancies in the requirement for the Wnt hairpin 3 (index finger) interaction with WLS during transport. Furthermore, the higher-resolution apo WLS structure resolves the previously unresolved ECL4 loop, implicating this region in the conformational transitions associated with Wnt binding, trafficking, and release. Taken together, the work adds incremental but valuable structural information and contributes to a more complete model of WLS conformational cycling during Wnt secretion.

---

Minor points
1 Line 282: The phrase "in the absence of Wnt" appears ambiguous. Is "in the absence of WLS" intended here?
2 Line 393: The authors propose that sphingomyelin (SM) can be accommodated within the WLS–Wnt structure, and Extended Data Fig. 8E shows SM docked into density assigned as POPC. Could additional experimental or simulation-based evidence be provided to strengthen this claim? Such data would be highly informative for clarifying the role of lipid composition in facilitating Wnt release.
3 The 1:1 WLS–Wnt5a structure was determined using a C-terminally tagged Wnt5a, whereas the dataset also contains 2:2 classes. What is the rationale for using an N-terminally tagged Wnt5a to determine the 2:2 structure? Do the authors have an estimate of the relative abundance of 2:2 classes in each dataset? Based on sequence comparison, are the residues forming the dimer interface unique to Wnt5a or distinguishable from other Wnts? Given that Wnt release assays are well established in the authors' laboratories, what are the challenges in experimentally validating the dimeric state of secreted Wnt5a?
4 The WLS–Wnt5a complex was determined in GDN detergent, whereas the apo WLS structure was solved in nanodiscs. Is the use of nanodiscs a key factor contributing to the higher resolution achieved for the apo structure?
5 Figure 2D (Lines 908–909): The description "two biological and technical replicates" would be clearer if the number of replicates (n = ?) and details of the statistical analysis were provided.
6 Extended Data Figs. 3 and 5: The particle orientation plots would benefit from being displayed in a format consistent with Extended Data Fig. 7.

Reviewer #4

(Remarks to the Author)

I co-reviewed this manuscript with one of the reviewers who provided the listed reports. This is part of the Nature Communications initiative to facilitate training in peer review and to provide appropriate recognition for Early Career Researchers who co-review manuscripts.

Version 1:

Reviewer comments:

Reviewer #1

(Remarks to the Author)
All my concerns have been fully addressed.

Reviewer #2

(Remarks to the Author)
We are all convinced that the authors have provided a thorough and comprehensive response, which has significantly strengthened the manuscript. We are therefore happy for you to proceed with publication

**Open Access** This Peer Review File is licensed under a Creative Commons Attribution 4.0 International License, which permits use, sharing, adaptation, distribution and reproduction in any medium or format, as long as you give appropriate credit to the original author(s) and the source, provide a link to the Creative Commons license, and indicate if changes were made.
In cases where reviewers are anonymous, credit should be given to 'Anonymous Referee' and the source.
The images or other third party material in this Peer Review File are included in the article’s Creative Commons license, unless indicated otherwise in a credit line to the material. If material is not included in the article’s Creative Commons license and your intended use is not permitted by statutory regulation or exceeds the permitted use, you will need to obtain permission directly from the copyright holder.
To view a copy of this license, visit https://creativecommons.org/licenses/by/4.0/

We sincerely thank the handling editor for providing the reviewers' comments and for inviting us to revise our manuscript. We are pleased that the reviewers recognized the scope and significance of our work. In this revision, we have incorporated extensive new experiments conducted in response to the reviewers' comments, along with changes to the text and figures that clarify issues brought up by the reviewers. These additions substantially strengthen our conclusions and broaden the general applicability of our findings on the mechanism of WLS-dependent secretion of both canonical and non-canonical Wnt ligands. We believe that the revised manuscript adequately addresses all the points raised by the reviewers, so we hope it is now acceptable for publication. Below, we provide a detailed, point-by-point response and sincerely thank the reviewers for their constructive feedback.

***Reviewer #1 (Remarks to the Author):***

*In this study, Ge et al. present cryo-EM structures of the Wnt5a–WLS complex and WLS alone, perform structure-guided mutagenesis and functional analyses, and use structural modeling to propose mechanisms by which WLS transports and presents WNTs to extracellular carriers. The authors further report that WNT5A–WLS can form a 2:2 quaternary complex and speculate that WNT5A may bind Frizzled receptors as a dimer, potentially distinguishing canonical and noncanonical WNT signaling. However, the structural insights provided by the WNT5A–WLS complex are limited, as the structure is nearly identical to previously reported WLS complexes with Wnt8, Wnt3a, and Wnt7. Moreover, the proposed mechanism that noncanonical WNT5A signals through dimerization is based on limited and preliminary evidence and is not convincingly supported by the current data.*

**<u>Response:</u>** We believe that several elements support the importance and novelty of our work. We provide the first systematic and quantitative dissection of Wnt transport and secretion, by incorporating Wnt carriers with high-resolution cryo-EM structural characterization, to elucidate the mechanism of WLS conformational cycling in these processes. We show that all three Wnt–WLS interfaces are essential for Wnt transport to the cell surface and for secretion by carriers, in contrast to earlier reports (PMID: 33357447; PMID: 34315898). By determining the high-resolution structure of apo WLS, we show that WLS conformational changes during Wnt release are similar to those of GPCRs upon activation, pointing to a role of the seven-transmembrane fold beyond signal transduction. Importantly, our apo WLS structure now resolves the ECL4 loop, and, together with functional experiments, demonstrate that this region regulates Wnt binding, transport and release, leading to a more complete model of WLS conformational cycling. We have also performed several new experiments which demonstrate that our findings apply to both canonical and non-canonical Wnts. Additional simulation analyses further strengthen our conclusions, arguing against the phosphatidylcholine gradient-driven Wnt release model (PMID: 37852257), instead supporting a mechanism in which WLS conformational changes act synergistically with transient carrier binding to mediate Wnt secretion. Our WLS–Wnt5a structure is the first for a non-canonical Wnt, and reveals an unexpected dimerization potential; although beyond the scope of the present study, this finding offers a framework for future investigation of non-canonical Wnt5a–ROR signaling. Finally, we determined the structure of apo WLS at 2.6 Å, despite its small size (~56 kDa) and dynamic nature; the data processing strategy we developed represents a significant advance in cryo-EM analysis of small membrane proteins, enabling unprecedented mechanistic insights.

*Specific Comments*

*1. Interpretation of the Wnt3a W150A/W152A mutant secretion defect*

*In Fig. 2, the authors show that the mWnt3a W150A/W152A mutant is no longer secreted extracellularly in a WLS-dependent manner. However, this phenotype may arise from at least two distinct mechanisms. One possibility is impaired binding to WLS, preventing transport to the plasma membrane. Alternatively, the mutation may disrupt interactions with extracellular Wnt carrier proteins (such as sFRPs or WIF), resulting in reduced retention at the cell surface or in conditioned medium.*

*Notably, the study cited by the authors (PMID: 34315898) employed autocrine signaling assays and demonstrated that Wnt3a W150A/W152A can still activate signaling in producing cells, suggesting*

*that the mutant can still be transported by WLS. These findings imply that the W150A/W152A mutation may primarily affect interactions with extracellular carriers rather than WLS-dependent transport.*
*The statement "This contrasts with previous reports suggesting that W150A has minimal impact on Wnt secretion and signaling; we speculate this discrepancy might be due to past studies not using physiological carriers to assay Wnt secretion" is therefore not logically consistent with the cited evidence and requires clarification or revision.*

**Response:** These are important points to clarify, and we thank the reviewer for raising them. The W150A and W152A mutants have greatly reduced cell surface localization and secretion by carriers; however, W150A was shown to be secreted and to signal in autocrine assays. Possible explanations for this discrepancy are the involvement of an unknown serum component in secretion, or that autocrine signaling arises from direct transfer of Wnt from WLS to Frizzled receptors. We have clarified these issues in the revised manuscript, as requested by the reviewer (see highlighted lines 207-216).

*2. Potential role of Afamin in Wnt3a mutant secretion*

*In the referenced study (PMID: 34315898), Wnt3a-conditioned medium was collected without adding exogenous of Wnt carrier proteins; under these conditions, secreted Wnt3a is expected to associate with serum-derived Afamin. The authors should therefore consider examining whether Afamin can facilitate secretion or extracellular stabilization of the Wnt3a W150A/W152A mutant, particularly given that sFRPs and WIF1 may inhibit Wnt–FZD interactions.*

**Response:** We thank the reviewer for suggesting this experiment. In response, we performed carrier-mediated secretion assays with purified AFM. As shown in Fig. 2C of the revised manuscript, the W150A and W152A mutations in Wnt3a abolish AFM-mediated secretion, similar to secretion by SFRP2, WIF1 and glypicans.

*3. Quantification of membrane-associated Wnt3a W333A*

*In Fig. 4B, the authors state that the Wnt3a W333A mutant exhibits reduced membrane-associated protein levels (line 235). However, based on the data presented, the membrane-associated signal of the W333A mutant appears higher than that of wild-type Wnt3a. This apparent discrepancy between the quantitative analysis and the displayed data should be clarified.*

**Response:** We thank the reviewer for identifying this labeling error, for which we apologize; we have corrected it in the revised Fig. 4B (WT: blue; W333A: red). As described in the manuscript, the Wnt3a W333A mutant shows reduced surface localization compared to WT.

*4. Generalization from Wnt3a to Wnt5a*

*Although Wnt3a and Wnt5a share sequence similarity, they exhibit distinct signaling properties and biological functions. When investigating the role of the hairpin structure in Wnt–WLS interactions and Wnt secretion, it would substantially strengthen the conclusions if comparable analyses were performed using Wnt5a.*

**Response:** We agree with the reviewer about the importance of comparable analyses using Wnt5a. In response, we performed a new set of experiments on Wnt5a, which strengthen our conclusions on the role of Wnt hairpins in WLS interaction and carrier-mediated secretion. Briefly, we demonstrate that Wnt5a mutations affecting hairpins 1 and 3 disrupt Wnt5a localization to the cell surface and secretion by the carriers SFRP2, AFM, WIF1 and glypicans (Extended Data Fig. 16 of the revised manuscript). Accordingly, we observe the same phenotype for WLS mutations that disrupt interactions with Wnt5a hairpins (A87W and G229Q; Extended Data Fig. 17, top row). Together, these results indicate that all three WLS–Wnt interfaces are required for cell surface trafficking and carrier-mediated secretion of both canonical and non-canonical Wnts, given that lipidated Hairpin 2 is already known to be essential across Wnts (see highlighted lines 247-255). Furthermore, we have now tested the effect of all WLS mutations (Fig. 6) on both Wnt3a and Wnt5a. As shown in Extended Fig. 17 of the revised manuscript, WLS mutations have similar effects on both ligands, indicating that release of canonical and non-canonical

Wnts is driven by conformational changes in the WLS transmembrane domain, together with previously unrecognized extracellular rearrangements of WLS, particularly involving ECL4 (see highlighted lines 325-328).

*5. Interpretation of the Ha helix and RECK interaction*

*Line 327 states: "Canonical Wnt3a also possesses this Ha helix, which would sterically clash with the Wnt7-specific coreceptor RECK, explaining why Wnt3a does not require RECK for signaling." This interpretation appears imprecise. If correct, it would imply that removal of the Ha helix from Wnt3a could enable RECK binding. The authors should clarify this point or revise the statement.*

**Response:** We apologize for the confusion. As requested by the reviewer, in the revised manuscript we clarify that we are speculating how the Ha helix might prevent Wnt3a binding to RECK, due to steric hindrance (see highlighted lines 337-338).

*6. Role of the Wnt N-terminal region in LRP5/6 binding*

*Line 331 states that "the Wnt N-terminal region is likely dispensable for LRP5/6 binding." However, previous work (PMID: 36994853) demonstrated that Tiki-cleaved Wnt3a (without N-terminus), secreted in complex with FZD-CRD, no longer interacts with LRP6. This discrepancy should be addressed and discussed.*

**Response:** We thank the reviewer for bringing up this important issue, which needs clarification. In the revised manuscript, we removed this sentence and we now discuss Tiki as follows (see highlighted lines 346-350): "… previous work has shown that the Wnt inhibitory protease Tiki cleaves the N-terminus of Wnt3a (PMID: 22726442). Although the resulting truncated Wnt3a remains capable of binding FZD-CRD, it fails to recruit the LRP6 co-receptor (PMID: 36994853). One possible explanation is that, while the N-terminus of canonical Wnts can adopt two opposite orientations, both may still permit binding to LRP5/6, an interaction required for full activation of the Wnt signaling pathway."

*7. Use of N-terminally Flag-tagged Wnt5a*

*In the first part of the manuscript, the authors used C-terminally FLAG-tagged WNT5A to co-express with WLS and determine the complex structure, which predominantly revealed a 1:1 WNT5A–WLS stoichiometry. However, when proposing the 2:2 quaternary complex, the authors used N-terminally FLAG-tagged WNT5A. The rationale for using different FLAG tag positions should be clearly explained. In addition, the possibility that a FLAG tag interferes with WNT5A behavior or assembly in structural analyses should be carefully considered.*

**Response:** We thank the reviewer for raising this issue, and we apologize for the confusion. In our original manuscript, we inadvertently omitted to mention that the N-terminal FLAG tag was removed with enterokinase, yielding native Wnt5a and thus avoiding artifacts that might have been caused by tagging. We have now added this explanation in the revised manuscript (highlighted lines 356-358), updated the Methods section, and we also include the SDS–PAGE and size-exclusion chromatogram of the final protein sample used for structure determination of the 2:2 Wnt5a–WLS complex (Extended Data Fig. 1). Importantly, the structure suggests that neither N- nor C-terminal tags would interfere with dimer formation. Although the reason is unclear, we observed more dimeric particles in the N-FLAG-removed sample, even though the size-exclusion profile is similar to that of the C-terminally FLAG-tagged sample (Extended Data Fig. 1), and both cryo-EM samples were of similar concentration.

***Reviewer #3 (Remarks to the Author):***

*In the manuscript entitled "Conformational cycling of the Wntless transporter drives trafficking and secretion of Wnt morphogens", Ge et al. describe cryo-EM structures of the WLS–Wnt5a complex, an improved-resolution apo WLS structure, and a previously unreported 2:2 WLS–Wnt5a assembly. Although the overall architecture of the WLS–Wnt5a complex closely resembles previously reported WLS–Wnt structures, the study introduces two potentially significant advances. Wnt5a is the first non-*

*canonical Wnt characterized in complex with WLS, and the dimeric interface observed in the 2:2 complex has not been described previously. However, these novel structural observations are not followed by focused functional interrogation. Instead, the majority of mutational and functional analyses are performed on canonical Wnt3a, for which higher-resolution structural information already exists. As a result, the mechanistic insight gained directly from the newly determined Wnt5a structures remains limited.*

*Nonetheless, the study highlights discrepancies in the requirement for the Wnt hairpin 3 (index finger) interaction with WLS during transport. Furthermore, the higher-resolution apo WLS structure resolves the previously unresolved ECL4 loop, implicating this region in the conformational transitions associated with Wnt binding, trafficking, and release. Taken together, the work adds incremental but valuable structural information and contributes to a more complete model of WLS conformational cycling during Wnt secretion.*

**Response:** We are glad that the reviewer appreciates the novelty and importance of our work. In response to the issues raised by the reviewer, we performed a new set of experiments on Wnt5a, which strengthen our conclusions on the role of Wnt hairpins in WLS interaction and carrier-mediated secretion. Briefly, we demonstrate that Wnt5a mutations affecting hairpins 1 and 3 disrupt Wnt5a localization on the cell surface and secretion by the carriers SFRP2, AFM, WIF1 and glypicans (Extended Data Fig. 16 of the revised manuscript). Conversely, we observe the same phenotype for WLS mutations that disrupt interactions with these Wnt5a hairpins (A87W and G229Q; Extended Data Fig. 17, top row). Together, these results indicate that all three WLS–Wnt interfaces are required for cell surface transport and carrier-mediated secretion of both canonical and non-canonical Wnts, given that lipidated hairpin 2 is already known to be essential across Wnts (see highlighted lines 247-255). Furthermore, we now tested the effect of all WLS mutations (Fig. 6) on both Wnt3a and Wnt5a. As shown in Extended Fig. 17 of the revised manuscript, WLS mutations have similar effects on both ligands, indicating that release of canonical and non-canonical Wnts is driven by conformational changes in the WLS transmembrane domain, together with previously unrecognized extracellular rearrangements of WLS, particularly involving ECL4 (see highlighted lines 325-328).

*Minor points*

*1 Line 282: The phrase “in the absence of Wnt” appears ambiguous. Is “in the absence of WLS” intended here?*

**Response:** We apologize for the confusion; we intended to convey that, in apo WLS, the Wnt hairpin 3–binding site at the top of the WLS luminal domain is dynamic and exhibits weak electron density. We have clarified this point in the revised manuscript (see highlighted lines 282-285).

*2 Line 393: The authors propose that sphingomyelin (SM) can be accommodated within the WLS–Wnt structure, and Extended Data Fig. 8E shows SM docked into density assigned as POPC. Could additional experimental or simulation-based evidence be provided to strengthen this claim? Such data would be highly informative for clarifying the role of lipid composition in facilitating Wnt release.*

**Response:** This is an important question. In response, we performed molecular docking and free energy calculations for DOPC and sphingomyelin (SM) binding to the observed cavity in the WLS–Wnt complex. Both phospholipids show high affinity for the binding site, with excellent docking scores of −14.714 and −14.140, and MM-GBSA $\Delta G_{bind}$ values of −125.62 kcal/mol and −112.10 kcal/mol, respectively. These values suggest that there is no clear preference for one or the other phospholipid; we believe this is not surprising, given the identical head groups of DOPC and SM, as well as the WLS cavity being able to accommodate the fatty acid chains. These results indicate a structural role for the phospholipid in WLS-mediated Wnt release (see highlighted lines 408-409). We show these data in Extended Data Fig. 8D-H, while the computational procedures are described in the Methods section of the revised manuscript. We thank the reviewer for this suggestion.

*3 The 1:1 WLS–Wnt5a structure was determined using a C-terminally tagged Wnt5a, whereas the dataset also contains 2:2 classes. What is the rationale for using an N-terminally tagged Wnt5a to determine the 2:2 structure? Do the authors have an estimate of the relative abundance of 2:2 classes in each dataset? Based on sequence comparison, are the residues forming the dimer interface unique to Wnt5a or distinguishable from other Wnts? Given that Wnt release assays are well established in the authors' laboratories, what are the challenges in experimentally validating the dimeric state of secreted Wnt5a?*

**Response:** We thank the reviewer for raising this issue, and we apologize for the confusion. In our original manuscript, we inadvertently omitted to mention that the N-terminal FLAG tag was removed with enterokinase, yielding native Wnt5a and thus avoiding artifacts that could have been caused by tagging. We have now added this explanation in the revised manuscript (highlighted lines 356-358), updated the Methods section, and we also include the SDS–PAGE and size-exclusion chromatogram of the final protein sample used for structure determination of the 2:2 Wnt5a–WLS complex (Extended Data Fig. 1). Importantly, the structure suggests that neither N- nor C-terminal tags would interfere with dimer formation. Although the reason is unclear, we observed more dimeric particles in the N-FLAG-removed sample, even though the size-exclusion profile is similar to that of the C-terminally FLAG-tagged sample (Extended Data Fig. 1), and both cryo-EM samples were of similar concentration. By quantifying the number of particles in the "good" 2D classes with clearly resolved structural features (see screenshots below, red boxes indicate 1:1 complexes and green boxes indicate 2:2 complexes), we found that 2:2 complexes account for ~4% of the sample with C-terminal FLAG-tagged Wnt5a and ~72% of the untagged sample.

2D class averages of the sample using C-terminally FLAG-tagged Wnt5a

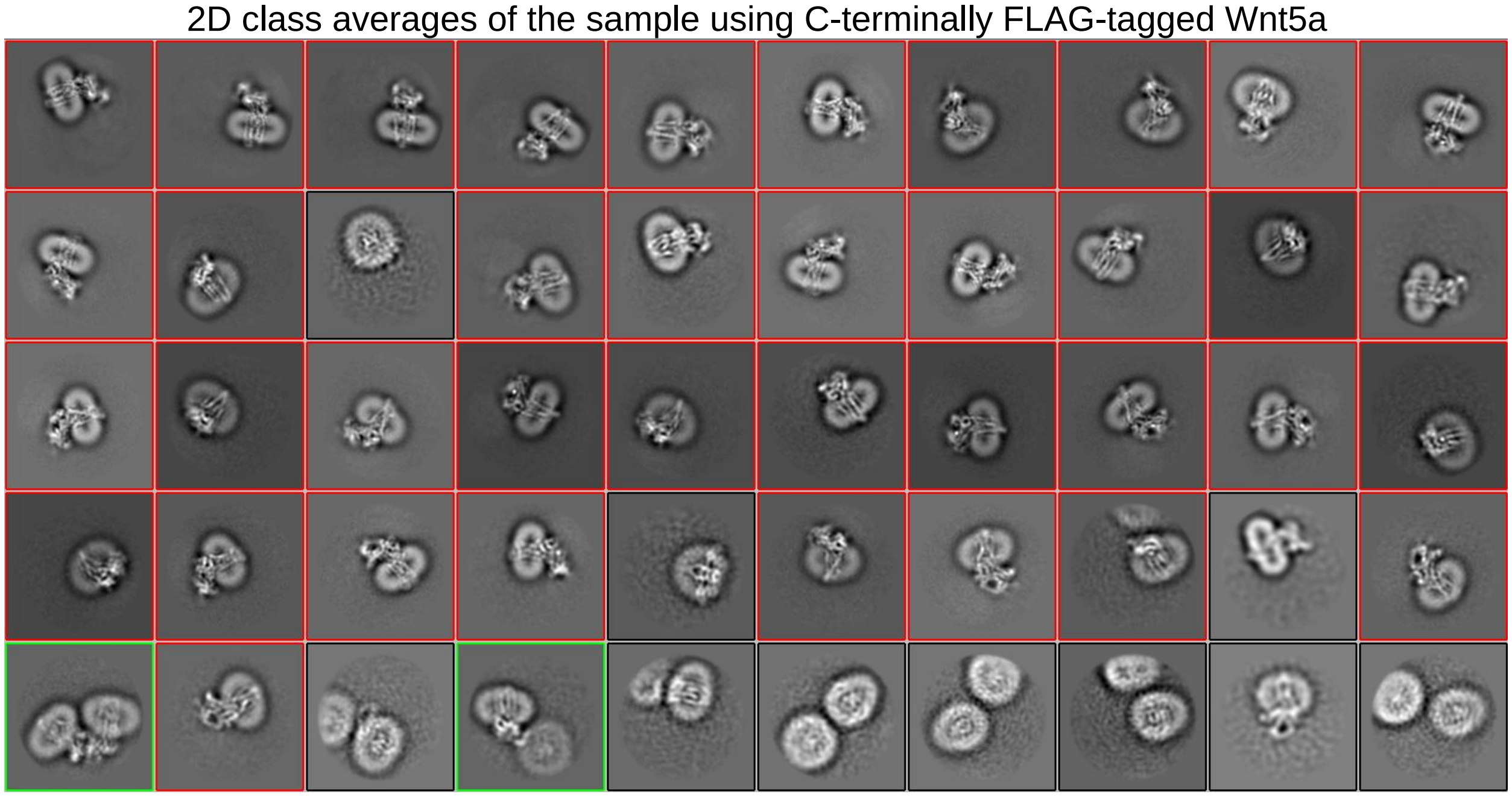

2D class averages of the sample with FLAG tag removed

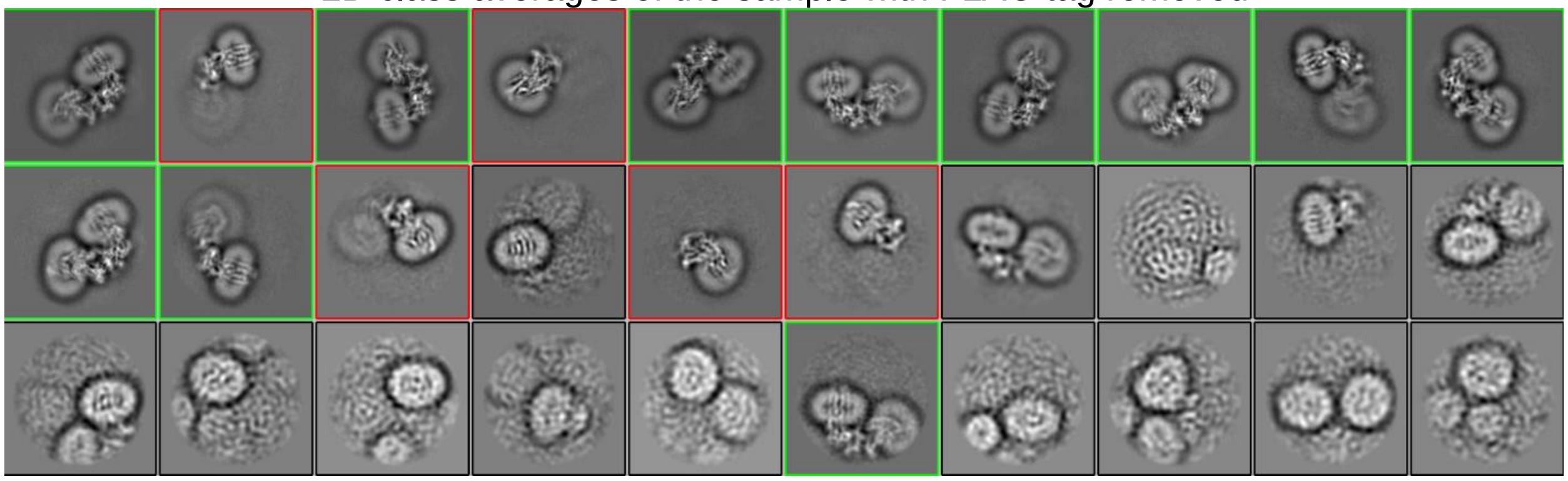

The dimer interface involves both conserved and variable residues, including the variable linker region between Wnt NTD and CTD (Extended Data Fig. 11). As the Wnt5a–WLS complex elutes as a 1:1 species in size-exclusion chromatography (Extended Data Fig. 1), we infer that dimerization likely forms at high concentration, such as used for cryo-EM grid preparation (5–10 mg/mL). It is possible that, upon Wnt5a binding to cell-surface FZD receptors and ROR1/2 co-receptors, the local Wnt5a concentration leads to its dimerization, forming higher-order signaling complexes. Validating this dimerization model in the context of non-canonical Wnt signaling is an important future direction for us, but is beyond the scope of the present study.

*4 The WLS–Wnt5a complex was determined in GDN detergent, whereas the apo WLS structure was solved in nanodiscs. Is the use of nanodiscs a key factor contributing to the higher resolution achieved for the apo structure?*

**Response:** We first tried to solve the structure of apo WLS in GDN, but we were unsuccessful; we speculate this is due to the small size of apo WLS and its dynamic nature in GDN micelles. We succeeded in solving a high-resolution structure of apo WLS in lipid nanodiscs, which was greatly facilitated by a cryo-EM data processing strategy we developed, which employs composite masks during 3D classification and refinement (Extended Data Fig. 4; see Methods). In brief, this approach separates the protein from the detergent micelle or lipid nanodisc, preserving their low-resolution features to aid reliable particle orientation determination while low-pass filtering their signals to suppress high-resolution overfitting, thereby leaving the protein signal unmodified.

*5 Figure 2D (Lines 908–909): The description "two biological and technical replicates" would be clearer if the number of replicates (n = ?) and details of the statistical analysis were provided.*

**Response:** We apologize for the confusion. All reported experiments were performed at least twice, and each experimental condition had two biological replicates. We are making this clear in the revised manuscript.

*6 Extended Data Figs. 3 and 5: The particle orientation plots would benefit from being displayed in a format consistent with Extended Data Fig. 7.*

**Response:** We apologize for the confusion. The final 3D refinements were performed using different software packages (cryoSPARC for Extended Data Figs. 3 and 5; RELION for Extended Data Fig. 7), which generate different formats for particle orientation plots.
